# Effects of biochar from algae (*Sargassum* spp*.)* on the fertility of two chlordecone contaminated West Indies soil

**DOI:** 10.1371/journal.pone.0338385

**Published:** 2025-12-30

**Authors:** Perrine Stephan, Pierre Leglize, Loïc Garcia, Lucas Charrois, Paméla Hartmeyer, Aude Fauvet, Séverine Piutti

**Affiliations:** 1 Université de Lorraine, INRAE, L2A, Nancy, France; 2 Université de Lorraine, INRAE, LSE, Nancy, France; 3 Université de Lorraine, INRAE, LAE, Nancy, France; University of Westminster - Regent Street Campus: University of Westminster, UNITED KINGDOM OF GREAT BRITAIN AND NORTHERN IRELAND

## Abstract

The unique properties of biochar (BC) have led to its increasing use as a soil management tool that improves soil fertility and enables pollutant sequestration. In the French West Indies, BC offers a potential solution for mitigating chlordecone (CLD) contamination. This study aims to comprehensively assess the impact of a *Sargassum*-derived BC (BCS), previously validated to sequester CLD, on soil fertility in the region. A one-year incubation experiment was conducted using two Caribbean soils (Andosol and Nitisol), testing amendments with BCS and activated carbon DARCO® (ACD) at 2% w/w. Fourteen indicators of chemical, biological, and physical fertility and CLD environmental availability were measured throughout the study. The results revealed that the carbonaceous amendments significantly affected soil fertility, in terms of soil and matrix properties. BCS had no effect on physical fertility but increased the availability of sodium (by a factor of 2–4 in both soils) and magnesium (by a factor of 1.5 in Andosol). BCS did not alter the activities of enzymes involved in the carbon cycle, although the activity of arylsulfatase in Andosol increased by 60% in the short and medium-term. However, BCS had a negative effect on leucine-aminopeptidase activity, raising concerns about its potential influence on the nitrogen cycle in soils.

## Introduction

In-depth studies of black soils in the Amazon, Germany and Australia have led to renewed interest in the application of charcoal to soils [1]. Indeed, various studies have demonstrated the central role that charcoal continues to play in improving and maintaining the fertility of these soils, because of its high porosity and high carbon and nutrient contents [2]. Thus, for several years, biochar (BC), which is composed of recalcitrant aromatic molecules, has been seen as an agronomic lever for carbon sequestration in soil r to mitigate climate change and improve soil fertility. However, even if historical data suggest that BCs are beneficial to soils, their effects on the three components of soil fertility (physical, chemical and biological) are variable, and the underlying mechanisms are complex.

BC has shown overall positive effects on the physical component of soil fertility. Most studies have demonstrated that the application of BCs to soils results in an average 10% reduction in bulk density [3,4], therefore improving water retention and facilitating root development [5]. BCs also appear to increase soil aggregate stability [3,4], thereby improving the circulation of water and nutrient availability for organisms and plants. However, other studies suggest that fine, dense soils are less responsive to BC applications. Indeed, improvements in aggregation, soil stability and water retention due to BC application are more pronounced in light-textured soils, such as sandy or sandy-loamy soils, and are less pronounced or even absent in clay soils [5,6].

The effects of BCs on chemical fertility are more variable. BC amendments in soils increase the dissolution of soluble organic matter, and the dissolved organic carbon, cation, and anion contents in the soil water solution, increasing both soil conductivity and pH [7]. BCs can also modulate biogeochemical cycles. With respect to the nitrogen cycle, BC amendments increase nitrification and mineralization in forest soils, although no similar effect has been observed in agricultural soils or grasslands [8,9]. Instead, an average 10% decrease in NH₄⁺ and NO₃^-^ availability has been recorded in agricultural soils after BC application, probably due to ion adsorption, which reduces leaching and maintains nitrogen availability [10,11]. The variability of the results obtained is strongly related to soil chemistry: BCs stimulate nitrification in acidic, phenol-rich soils with low initial nitrification activity but have a minimal effect on soils with initially high nitrification activity [8]. BCs significantly increase the availability of phosphorus (P), with a meta-analysis by Gao et al., (2019) reporting an average increase of 45%. This increase is related to the high content of metals (Al3 ⁺ , Fe3⁺and Fe2⁺) in BC, which complex phosphorus and reduce leaching.

With respect to biological fertility components, the effects of BCs on soil fauna, microbial diversity and enzyme activities are complex and variable. The porous structures of BCs provides habitats for microbial colonization and supports growth by storing labile carbon and water in their pores [12–14]. In some cases, BCs may not significantly affect microbial biomass abundance [15,16] but can alter microbial community structure, with an increase in the abundance of the phylum Gemmatimonadetes (+19.8%) and a decrease in the abundance of the phylum Acidobacteria (−14.6%) [17]. BCs have variable effects on alpha and beta microbial diversity [17,18]. Recent meta-analyses have highlighted changes in soil microbial communities in response to BCs that occur according to the BC properties and soil type [17,19,20]. The ability of microorganisms to synthesize intracellular and extracellular enzymes can also be modulated by BCs, thus influencing the decomposition and mineralization of soil organic matter (Alisson et Vitousek, 2005). These effects can be direct, through enzyme adsorption on BC surfaces, or indirect, through the modification of soil properties that regulate enzyme production [21]. Foster et al., (2018) reported that the surface area of BC can adsorb and protect enzymes, thereby modifying their activity. Despite mixed results [22,23], meta-analyses have demonstrated a reduction in the activity of enzymes involved in the carbon cycle, and an increase in the activity of enzymes involved in the nitrogen and phosphorus cycles [24].

In addition to their beneficial effects on soil fertility, BCs have a highly porous and reactive structure with a variety of functional groups that enable them to sequester organic and inorganic pollutants in soil. This property has made BCs increasingly popular for soil remediation since the 2000s. Studies have demonstrated promising results for the sequestration of various herbicides, such as atrazine [25] and simazine [26]; insecticides, such as carbofuran and chlorpyrifos [27]; and even some polycyclic aromatic hydrocarbons (PAHs) [28]. The application of BCs could offer numerous advantages, particularly in regions such as the French West Indies, where the mitigation of pollution and the enhancement of soil fertility represent significant soil management challenges. However, to optimize the use of BCs, it is essential to clearly define the main objective –pollution reduction or fertility improvement – to adapt the technology to the specific context. Indeed, the conditions under which BCs are applied to increase fertility are generally different from those for contaminant sequestration. For instance, higher doses of BCs generally promote pollutant sequestration [26] but can affect soil fertility, with potentially negative effects as the doses increase [11,24].

In Guadeloupe and Martinique, soils are heavily contaminated by chlordecone (CLD), a persistent pesticide affecting not only soil but also agricultural products and local populations [29–31]. One proven strategy to mitigate CLD contamination is the use of BC from *Sargassum* spp. (BCS), an invasive algae impacting the French West Indies. Previous studies of BCS have shown promising results for CLD sequestration [32,33], but these studies focused solely on remediation and did not assess potential impacts on soil fertility. With approximately 20% of Guadeloupe’s farmland and 40% of Martinique’s soil being contaminated [34], understanding and thoroughly assessing the impact of BCS on West Indies soils is crucial, especially given the specific characteristics of the raw material (*Sargassum* spp) used. Algae are a well-known source of biomass for agricultural fertilization [35]. Brown, green and red microalgae and macroalgae are widely used as biofertilizers because of their high protein, mineral and pigment contents. However, they are generally used as direct inputs, such as compost, powders (produced by drying and grinding), extracts of molecules of interest, or soluble formulations [36]. The production of BC from algae remains underdeveloped, and studies on its effects on soil fertility are limited. However, some previous studies have tended to demonstrate the benefits of using algae as a raw material for biochar production. In particular, these studies highlight the interesting physical properties of these biochar, characterized by a generally high specific surface area and a micro- and mesoporous distribution, which is conducive to the development of microorganisms. Furthermore, wing to the nature of algae, the predominant functional groups present on BC are phenolic, carboxylic and hydroxyl groups, which are conducive to nutrient retention [37]. However, specific studies on the potential of algae in the form of BC as a biofertilizer, the specific characteristics of the type of algae used (micro, macro, brown, green or red), and the different responses of various soil types are lacking [37]. Thus, it is feasible to use algae (*Sargassum* spp*.)* to produce BC in the French West Indies, with the goal of improving soil health by combining remediation and improved fertility.

The volcanic soils of the French West Indies are also very different from those in mainland France: they are often rich in organic matter and clay, but poor in phosphorus, which is essential for plant growth, and their acidity can increase the risk of aluminium toxicity [38,39]. These unique soil properties, combined with specific climatic conditions, contribute to initial soil fertility that remains underexplored.

The objective of this study is to assess the impact of BCS on the fertility of Caribbean soils under conditions representative of a first remediation application (manufacture and implementation). On the basis of the literature and considering the particular nature of the raw material used (*Sargassum* spp.), we hypothesize that the biochar produced from these algae (BCS) will affect the overall fertility of Caribbean soils in the context of remediation. More specifically, we hypothesize that this biochar will not significantly affect the physical properties of the soils, because of their clayey texture but will increase the availability of major nutrients (P, K, Ca and Na) and trace elements (Zn, Fe, Cu, As and Pb). Finally, we hypothesize that BCS will stimulate microbial biomass, although the presence of certain trace metals (As and Pb) may also disrupt the activity of certain enzymes.

## Materials and methods

### West Indies soils

Two representative West Indies soils, a Nitisol (halloysite clay) and an Andosol (allophane clay), were sampled from field plots in areas described as heavily contaminated with CLD in the South Basse-Terre, Guadeloupe [40]. These plots were identified in Karugeo (https://www.karugeo.fr/accueil), a public geographic information platform. Andosols were sampled at the “Bois Boulé” and “Concession” locations [32]. The Nitisols were sampled at “Trois Rivières” and at “La petite savane”. For both soils, the topsoil (0–15 cm) was sampled for experimentation to ensure a high CLD concentration. The different samples of the soils were then combined to generate a composite sample. The soils were sun-dried in a greenhouse (to a water content of 18 ± 3%) and sieved at 2 cm before being sent to Vandoeuvre-lès-Nancy (France). Once received, the soils were sieved to 5 mm for homogenization and to remove residues and stone. They were stored at 4°C before use in the incubation experiments. The total CLD concentrations were measured (Labocea, Ploufragan, France), and physicochemical analyses were performed (Celesta-lab, Mauguio, France). The main physicochemical properties and CLD concentrations are summarized in **Table 1**.

**Table 1:** Physicochemical properties and CLD concentrations of the soils used in the experiment.

							*Exchangeable cations*				
	Water field capacity (%)	Organic matter (%)	pH	CEC_metson_ (cmol^ + ^.kg^-1^)	C/N	Olsen P_2_0_5_ (mg.kg^-1^)	K_2_O (cmol^ + ^.kg^-1^)	MgO (cmol^ + ^.kg^-1^)	CaO (cmol^ + ^.kg^-1^)	Na_2_O (cmol^ + ^.kg^-1^)	CLD (mg.kg^-1^)
Andosol	46.85	8.87 ± 0.71	7.3	44.8 ± 2.6	10.8	94.5 ± 9.2	2.13 ± 0.08	1.66 ± 0.10	37.2 ± 2.78	0.17 ± 0.02	1.85 ± 0.74
Nitisol	35.65	7.45 ± 0.62	6.3	39 ± 2.4	9.0	347 ± 27	3.72 ± 0.12	5.66 ± 0.31	23.8 ± 1.86	0.54 ± 0.04	7.99 ± 3.19

When available, measurement uncertainties were provided (mean ± SE).

### Biochar origin and characterization

The biochar used in this study comes from *Sargassum* spp. (BCS) and was produced by the COVACHIM-M2E laboratory (Guadeloupe, France). This biochar has already been evaluated for its ability to sequester CLD [32]. In addition, a DARCO® activated carbon (ACD), recognized for its high sequestration capacity but rarely studied for its agronomic impacts, was also tested. The matrix properties were characterized by several laboratories: LIEC (Vandoeuvre-lès-Nancy, France) for textural characteristics; COVACHIM-M2E (Guadeloupe, France) for surface chemical properties; and WESSLING (Lyon, France), for elemental characterization. These analyses revealed that the BCS has a BET surface area of 855.2 ± 9.6 m^2^.g-¹, a mesoporous volume of 0.44 cm^3^.g-¹, a microporous volume of 0.21 cm^3^.g-¹ and a pH at the zero-charge point (pH_zcp_ of 6.5. Its elemental composition is 80.2% C, 16.7% O, 2.0% S, 0.6% Mg, 0.6% Ca and 0.06% Al. The concentrations of sulfates, chlorides and nitrates are 17,000, 4,000 and <200 mg.kg-¹ dry matter, respectively, while the total amounts of sodium, phosphorus, calcium and potassium are 6120, 6490, 17500 and 7700 mg.kg-¹ raw material, respectively. With respect to ACD, it has a BET surface area of 793.8 ± 14.5 m^2^.g-¹, a mesoporous volume of 0.30 cm^3^.g-¹, a microporous volume of 0.39 cm^3^.g-¹ and a pH_zcp_ of 6.98. Its elemental composition is limited to 95.8% C and 4.2% O, with sulfate, chloride and nitrate concentrations below 200 mg.kg-¹ dry matter and sodium, phosphorus, calcium and potassium concentrations of 696, 83, 2010 and 332 mg.kg-¹ raw matter, respectively.

### Experimental design

**Fig 1:**
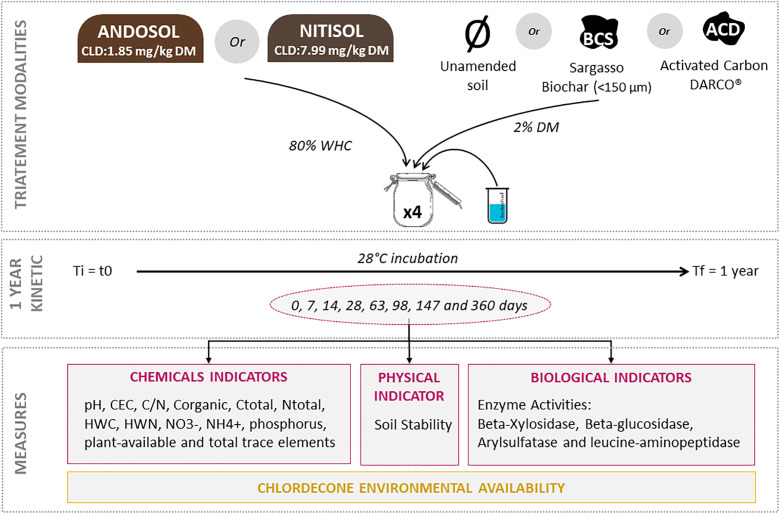
Experimental design.

The experiment involved three soil modalities, with four replicates per modality, in the following combinations: UA (control without carbon amendments), BCS (2% w/w *Sargassum* spp. BC amendment, amendment rate previously determined [33]) and ACD (2% w/w AC DARCO® amendment). Incubation was conducted in mesocosms over a one-year period with regular sampling dates (0, 7, 14, 28, 63, 98, 147 and 360 days), which varied according to the fertility indicator tested (S1 Table). The soil was moistened to 80% of its water holding capacity (WHC) using distilled water. For each sample, 25–50 g of soil was placed in closed 0.5 L glass jars and incubated at 28 ± 1 °C. Each jar represents one of the four replicates per modality and sampling date, for a total of 96 jars per soil studied. During the incubation period, the jars were regularly aerated to prevent the development of anaerobic conditions. Fourteen indicators were measured in the three main fertility groups (chemical, biological and physical) and grand the environmental availability of CLD was monitored over the incubation period (**Fig 1**).

### Assessment of the environmental availability of chlordecone

As the matrices tested in this study are intended primarily to reduce the risk of CLD contamination in soil, the environmental availability of this molecule in the presence of the tested matrices was monitored in addition to fertility measurements. These measurements were carried out according to an adaptation of the ISO/DIS 16 751 test (method A) previously used and described by Stephan et al., (2023).

### Soil sample conditioning

In accordance with a specific protocol (S2 Table), measurements on moist soil were carried out within 24–48 hours (after the soil was stored at 4 °C). The remaining soil was dried (40 °C for 72h h), stored at 20 ± 4 °C in the dark and ground(150µm) using a ball mill for 1 min at a frequency of 30 Hz according to the ISO 11464:2006 standard.

### Fertility indicators

Fourteen fertility indicators were evaluated during the one-year period. Most of the measurements were carried out according to the associated ISO protocols (S2 Table). However, three internal protocols from the LAE laboratory (Vandoeuvre-lès-Nancy, France) were applied for the measurements of soil available nitrate and ammonium, hot water carbon (HWC) and nitrogen (HWN) extraction and enzyme activity.

**Soil available nitrate and ammonium.** Five grams of fresh soil was mixed with 50 mL of 0.016M KH_2_PO_4_ solution. After stirring for 30 min, the suspension was filtered through Whatman 42 filter paper and then through a 0.2 μm Sartorius filter. The nitrate and ammonium contents were then analysed by using ion chromatography [41].

**Hot water carbon (HWC) and nitrogen (HWN) extraction** [42]. Six grams of fresh soil was mixed with 30 mL of ultrapure water. After 18 h incubation at 70 °C, the suspension was filtered through Whatman 42 filter paper and then analysed with a TOC-V CSH analyser (by SHIMADZU).

**Enzyme activities.** The activities of four enzymes, β-glucosidase, β-xylosidase, arylsulfatase and leucine-aminopeptidase, were determined using colorimetric methods [43–45]. The ISO 22939 fluorometric method was not applied here, as pretesting showed that the data was significantly altered because of the presence of BC or activated carbon (AC) in the soil samples. For β-glucosidase, β-xylosidase and arylsulfatase activity determination, 5 g of fresh soil was mixed with 0.5 mL of toluene, and five minutes later, 20 mL of sodium acetate buffer (0.5 M, pH 5.8) was added. The mixture was stirred for 10 min after which2x2 mL was taken (test and blank). Next, 0.5 mL of appropriate substrate (S3 Table) was added to the test samples, and the blank and test samples were incubated at 37 °C for 1 or 3 hours, depending on the enzyme (S3 Table). To stop the reaction, 0.5 mL of CaCl_2_ (0.5 M) and 2 mL of NaOH (0.5 M) were added to all the samples (blank + tests) at the end of the incubation period. Afterward, 0.5 mL of specific substrate was added to the blank. The test and blank mixtures were filtered through Whatman 2V filter paper, 0.2 ml of the filtrate was placed in a microplate, and the absorbance was measured at 405 nm (Synergy HT, BioTek). For leucine-aminopeptidase, 5 g of fresh soil was mixed with 20 mL of Tris-HCl (0.1 M at pH 8) and stirred for 10 min. Next, 2x4 mL was taken (test and blank), and 1 mL of the appropriate substrate (S3) was added to the test samples. Afterwards, all the samples were incubated at 37 °C for 3 hours. To stop the reaction, 4 mL of acetic acid was added. One milliliter of substrate was then added to the blank samples, and all samples were filtered through Whatman 2V filter paper. For the other enzymes, 0.2 mL of filtrate was placed in a microplate, and the absorbance was measured at 405 nm (Synergy HT, BioTek).

Enzyme activities were determined using calibration curves in which the absorbance versus the p-nitrophenol concentration for β-glucosidase, β-xylosidase and arylsulfatase or p-nitroaniline for leucine-aminopeptidase was plotted. All activities are expressed as mg of p-nitrophenol or p-nitroaniline g^-1^ dry soil h^-1^.

**Enzyme Activity correction.** As with the fluorometric method, the colorimetric method showed a bias in the presence of sequestration matrices. Following verification, it was determined that during the incubation period, BCS and ACD were able to absorb a fraction of the p-nitrophenol or p-nitroaniline liberated after the substrate had been hydrolyze, thereby altering the results. Thus, correction factors specific to each enzyme, matrix and soil were determined and applied to our data. To this end, an experiment was conducted with the studied soil samples (UA, BCS and ACD) incubated at 28 °C. The same protocols were used for the enzyme activity measurements, with the only difference being that the substrate was not added to the soil; instead solutions of the enzyme product (p-nitroaniline or p-nitrophenol) was added at three different concentrations, 2.5, 10 and 20 μg.mL^-1^. In this way, determining the relationship between the measured concentrations of these products in the BCS and ACD modalities, and the fractions they represent in relation to the theoretical concentrations was possible.

### Data preparation

All the compiled data were subjected to preprocessing, resulting in a dataset without anomalies that was suitable for subsequent statistical processing and analysis. First, with the support of laboratory notebooks, data resulting from errors in laboratory handling were removed (replaced by *NA* in the database). Afterwards, abnormal values were identified among the 4 repetitions carried out for each indicator, date and modality and tested via the *grubbs.test* function. All data with a *p-value* less than 1% were considered aberrant and therefore removed from the dataset.

### Relative environmental availability factor calculation

The relative environmental availability factor (REA) as the relative bioavailability factor (RBA) [46] is determined as the ratio between the concentration of CLD in the chosen modality and the concentration of CLD in the control (set to 100% as a reference).


$${\text{REA}}_{\text{time},\text{modality}}\;=\frac{[\text{CLD}]time;modality}{[\text{CLD}]time;control}\times \text{1}\text{0}\text{0}$$

*Modality:* soil amended with BCS or ACD.

*Time:* 2, 14, 63, 98, 147 or 360 days.

*Control:* unamended soil.

### Statistical analysis

All statistical analyses for this study were carried out using R studio (version 4.4.0). First, principal component analysis (PCA) and hierarchical ascending classifications (HAC) were performed to describe the dataset, using the *prcomp*, *PCAtools* and *FactoMiner* packages. The *missMDA* function was used to replace missing data (NA) in the database by determining the most likely value on the basis of all the coordinates of the remaining values, thus avoiding the creation of biased values. A comparative approach between modalities at each sampling time point and by indicators was applied. For this, one-factor ANOVAs were used, with the fertility indicators as the quantitative factor and the modality as the qualitative factor. Finally, on the basis of the results of the previously performed PCA and research into the mechanisms underlying the effects of BC on soils [7], the measurement dates in this study were grouped into three periods: short-term, medium-term and long-term, depending on the soils tested. One-factor ANOVAs were also carried out on the basis of these phases and by fertility parameters. For all ANOVA tests, differences with a *p-value* < 0.05 were considered significant.

## Results

### Effects of the matrices on the environmental availability of chlordecone in soils

First, the initial concentration of CLD in the two soils tested differed: 1.85 ± 0.74 mg.kg^-1^ DM for the andosol and 7.99 ± 3.19 mg.kg^-1^ DM for the Nitisol (**Table 1**). For each matrix tested (BCS and ACD), CLD sequestration was higher in Andosol than in Nitisol (**Table 2**). At the end of the experiment (360 days), the environmental availability of CLD was reduced in the Nitisol by 46% (5.04 ± 0.99 mg CLD.kg^-1^ DM) and by 79% (2.01 ± 0.21 mg CLD.kg^-1^ DM) for BCS and ACD, respectively. In the Andosol, the reductions were approximately 60% (0.82 ± 0.03 mg CLD.kg^-1^ DM) for BCS and 90% (0.14 ± 0.01 mg CLD.kg^-1^ DM) for ACD. These results also highlight statistically significant matrix effect for both soils. From the very first days of incubation, ACD outperformed BCS. After 360 days, REAs of 21.4% (Nitisol) and 6.51% (Andosol) were achieved with ACD, whereas these values were 53.7% (Nitisol) and 38.3% (Andosol) with BCS. With respect to the Andosol, the impact of BCS on the environmental availability of CLD did not change significantly between Day 2 and Day 147. The same observation was made for the Nitisol, but for both matrices tested. In the case of BCS, at least 50% sequestration was reached in the first few months for the Andosol (Days 63 and 98) and after 1 year for the Nitisol.

**Table 2:** Relative environmental availability (REA) of nitisol and andosol over the kinetic.

Time (days)	Modality	Nitisol		Andosol	
		REA (%)	Confidence interval of 95%	REA (%)	Confidence interval of 95%
2	UA	100	[102-98.4]	100	[100-99.8]
	BCS	81.9	[82.1-81.8]	41.6	[42.0-41.3]
	ACD	36.3	[37.5-35.2]	32.7	[33.2-32.3]
14	UA	100	[102-97.8]	100	[100-99.7]
	BCS	79.6	[81.1-78.2]	50.4	[50.6-50.2]
	ACD	28.1	[29.1-27.1]	17.2	[17.4-16.9]
63	UA	100	[102-97.7]	100	[100-99.8]
	BCS	82.0	[82.9-81.1]	52.7	[52.8-52.5]
	ACD	35.9	[38.2-33.7]	14.1	[14.3-14.0]
98	UA	100	[102-98.3]	100	[100-99.8]
	BCS	88.0	[88.8-86.2]	49.8	[49.8-49.7]
	ACD	27.6	[28.6-26.7]	13.6	[13.7-13.4]
147	UA	100	[103-97.5]	100	[100-99.7]
	BCS	83.1	[86.1-80.2]	41.8	[42.1-41.5]
	ACD	30.8	[32.9-28.7]	8.74	[8.77-8.71]
360	UA	100	[105-95.3]	100	[100-99.4]
	BCS	53.7	[56.8-50.5]	38.3	[38.4-38.2]
	ACD	21.4	[22.1-20.7]	6.51	[6.53-6.48]

The REA factor of CLD is expressed as percentage relative to that of unamended soils (Nitisol and Andosol). UA: unamended soil. BCS: Biochar of *Sargassum* spp.. ACD: Activated carbon DARCO®. The values correspond to the mean (n = 4)

### Global analysis of Andosol and Nitisol fertility

A first principal component analysis (PCA1) was carried out to understand the effects of the matrices tested on the fertility of Andosol and Nitisol. The first two dimensions of PCA1, based on the two soils (S1 Fig) explain 58.6% of the observed differences between individual samples. PCA1 revealed that the first component (dim1) explained 46.3% of the total variance and enabled the two groups of individual samples to be strongly discriminated. These two groups were characterized by the type of soil tested: Andosol or Nitisol (S1 Fig). The variables that contributed the most to the structuring of the first dimension were certain TMEs (Zn, Ni, As, Pb, Cd and Fe) and nutrients (P, K, Ca and Na), pH and the activities of two enzyme, arylsulfatase and β-xylosidase (S2 Fig). Compared with the Nitisol samples, the Andosol samples show negative coordinates along dim1 and significantly higher enzyme activities. Specifically, the mean arylsulfatase activity was 478 ± 19.0 µg.g^-1^ DM.h^-1^ for Andosol and 70.3 ± 3.07 µg.g^-1^ DM.h^-1^ for Nitisol. Similarly, the β-xylosidase activity averaged 150 ± 6.95 µg.g^-1^ DM.h^-1^ for Andosol and 79.7 ± 3.32 µg.g^-1^ DM.h^-1^ for Nitisol. The Nitisol samples had positive coordinates along axis 1, with mean values significantly higher than those for Andosol for Zn (mean Nitisol 29 ± 0.67 mg.kg^-1^ DM and mean Andosol 6.1 ± 0.08 mg.kg^-1^ DM), P (mean Nitisol 182 ± 3.65 mg.kg^-1^ DM and mean Andosol 39 ± 0.58 mg.kg^-1^ DM) and Ni (mean Nitisol 213 ± 4.76 µg.kg^-1^ DM and mean Andosol 77 ± 1.7 µg.kg^-1^ DM). A disparity between the individual samples was also noted on the second dimension of PCA1, characterized mainly by carbon and nitrogen contents in their various forms (NH_4_+ , NO_3_^-^, HWC, HWN and C_org_) and by leucine-aminopeptidase activity. However, this disparity remains less pronounced (12.3%) than that along the first dimension, demonstrating the predominance of the soil effect over other possible effects, such as those of the matrices tested, and the need to perform individual analyses for each soil.

### Response of Andosol fertility to biochar and activated carbon amendment

**Fig 2:**
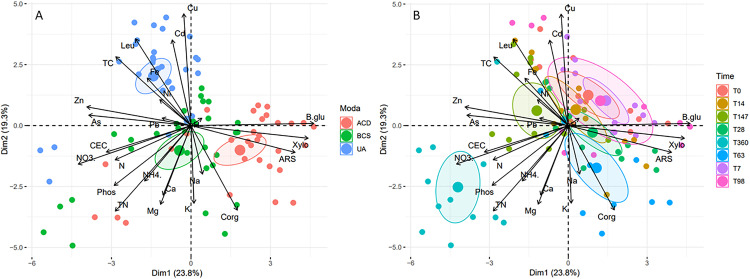
Principal component analysis (PCA2) of the soil parameters measured for (A) each modality and (B) the incubation duration. Points represent the coordinates of each individual sample according to the 2 dimensions of PCA2 (red: ACD; green: BCS; blue: UA or red: t0; violet: t7; orange: t14; green: t28; blue: t63; pink: t98 green-brown: t147 and cyan: t360). TC = HWC, TN = HWN. Confidence ellipses (95%) were plotted for each treatment.

A second principal component analysis (PCA2) was carried out to assess the impact of the carbon matrices tested (BCS and ACD) on the different fertility parameters when only Andosol was considered. Amendment modality and incubation time were used as illustrative variables in **Fig 2A** and **Fig 2B**. The first two dimensions of PCA2 explained 43.1% of the total variance. First, when focusing on the modality (Fig 2A), we observed that the control individual samples (UA) could be discriminated from the BCS and ACD individual samples mainly by dimension 2, with positive coordinates for control soils and negative coordinates for amended soils on this axis. This distinction seemed to be characterized by certain TMEs (Cu and Cd), and nutrients (Mg, K, Ca and P), HWC, HWN, the Corg content and leucine aminopeptidase activity (S3 Fig). However, among these variables, Cu made the greatest contribution (>15%, S3 Fig) to this dimension (mean UA 7.6 ± 0.17 mg.kg^-1^ DM; mean BCS 7.0 ± 0.15 mg.kg^-1^ DM; mean ACD 6.6 ± 0.14 mg.kg^-1^ DM). A single individual between the UA and ACD modalities was also observed along dim 1. This axis also discriminates between the two carbon matrices tested (ACD and BCS) (Fig 2A). β-glucosidase, β-xylosidase and arylsulfatase activities; Zn, NO_3_^-,^ P and total N concentrations; and HWN, HWC and CEC contributed to this first dimension. However, the most discriminant parameters were the activities of carbonrelated enzymes such as β-glucosidase (mean UA 117 ± 7.25 µg.g^-1^ DM.h^-1^; mean BCS 130 ± 12.4 µg.g^-1^ DM.h^-1^; mean ACD 187 ± 15.1 µg.g^-1^ DM.h^-1^) and β-xylosidase (mean UA 100 ± 6.18 µg.g^-1^ DM.h^-1^; mean BCS 112 ± 12.0 µg.g^-1^ DM.h^-1^; mean ACD 210 ± 21.5 µg.g^-1^ DM.h^-1^). The effect of incubation time (**Fig 2B**) revealed the opposite effect on the individual samples at 360 days compared with that the other measurement dates. For the 360-day measurements, although all the modalities (UA, BCS and ACD) differed from the other incubation dates, the three modalities were still distinguished from each other (S4 Fig).

Hierarchical ascending classification (HAC) enabled us to group homogeneous individual samples into four classes (S5 Fig). The first, most distinct group was composed of 11 individual samples from all three modalities at 360-days of incubation. The second group included the remaining 28 UA individual samples. The two remaining classes were distinguished by the two types of carbon matrix used. An enclave of individual samples from the ACD modality at 147 days of incubation was, however, observed in class 4, corresponding to individual samples amended with BCS (S5 Fig). From these groups, it was possible to identify two different temporal phases in the response of the Andosol to BC and AC amendments. The first, denoted short-medium term (SMT), covers Days 0–147, whereas the second, denoted long term (LT) corresponds to Day 360.

### Response of Nitisol fertility to biochar and activated carbon amendment

**Fig 3:**
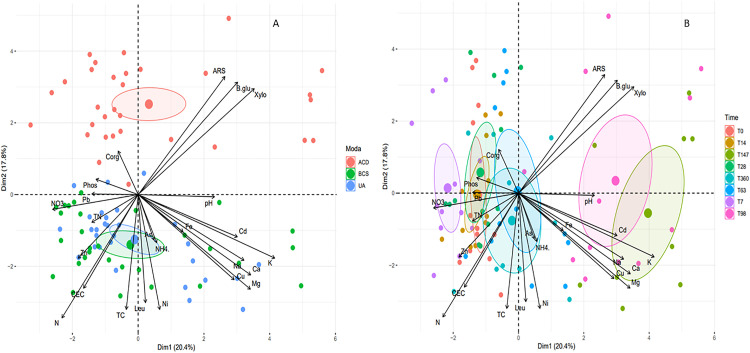
Principal component analysis (PCA3) generated from the soil parameters measured for (A) each modality and (B) the incubation duration. Points represent the coordinates of each individual samples according to the 2 dimensions of PCA2 (red: ACD; green: BCS; blue: UA or red: t0; violet: t7; orange: t14; green: t28; blue: t63; pink: t98; green-brown: t147 and cyan: t360). TC = HWC, TN = HWN. Confidence ellipses (95%) were plotted for each treatment.

A third principal component analysis (PCA3) was carried out to assess the impact of the carbon matrices tested (BCS and ACD) on the different fertility parameters considering Nitisol only. Amendment modality and incubation time were used as illustrative variables for **Fig 3A** and **Fig 3B**. The first two dimensions of PCA3 explained 38.2% of the total variance. The third dimension of PCA3 was also important to consider, since its contribution (17.4%) to individual samples differentiation was similar to that of the second dimension (17.8%) (S6 Fig). For this soil, individual samples from the UA and BCS modalities overlapped, and differed from the individual samples from the ACD. ACD discrimination was due mainly to the second dimension and was largely characterized by the activities of all the enzymes, CEC, HWC, total N content, certain TMEs (Ni and Cu), and Ca and Mg concentrations (S8 Fig). Among these variables, those that contributed the most to the construction of the second dimension (>7.5%) were: β-glucosidase activity (UA mean: 68.2 ± 3.46 µg.g^-1^ DM.h^-1^, BCS mean: 59.4 ± 5.03 µg.g^-1^ DM.h^-1^, ACD mean: 105 ± 6.06 µg.g^-1^ DM.h^-1^), β-xylosidase activity (UA mean: 36.5 ± 1.82 µg.g^-1^ DM.h^-1^, BCS mean: 27.3 ± 2.54 µg.g^-1^ DM.h^-1^, ACD mean: 63.7 ± 5.06 µg.g^-1^ DM.h^-1^), arylsulfatase activity (UA mean: 61.7 ± 1.97 µg.g^-1^ DM.h^-1^, BCS mean: 49.1 ± 4.40 µg.g^-1^ DM.h^-1^, ACD mean: 89.3 ± 5.31 µg.g^-1^ DM.h^-1^), the total N concentration (UA mean: 4.9 ± 0.04 g.kg^-1^ DM, BCS mean: 5.1 ± 0.05 g.kg^-1^ DM, ACD mean: 4.6 ± 0.05 g.kg^-1^ DM), HWC (UA mean: 1084 ± 64.7 µg.g^-1^ DM, BCS mean: 905 ± 46.8 µg.g^-1^ DM, ACD mean: 530 ± 20.3 µg.g^-1^ DM) and the Ni concentration (UA mean: 0.21 ± 0.01 mg.kg^-1^ DM, BCS mean: 0.25 ± 0.01 mg.kg^-1^ DM, ACD mean: 0.19 ± 0.01 mg.kg^-1^ DM). Considering the incubation time as an illustrative variable (**Fig 3B**), individual samples at 98 and 147 days were distinguished from those from the other dates along Axis 1. The main contributors to this dimension include most of the variables listed for dim 2. Indeed, their representations in the variable circle (S8 Fig) were for the most part equidistant from Axes 1 and 2. Nevertheless, the K concentration alone made a strong contribution to this first dimension (S7 Fig). For individual samples at 98 and 147 days, the ACD modality could be discriminated from the other two modalities and from the other measurement dates (S9 Fig).

Four clusters were identified using HAC (S10 Fig). The first, composed of 15 individual samples, grouped the three modalities on Day 98 and for the UA modality on Day 147. The second, composed of 19 individual samples, grouped the three modalities at Day 360 plus BCS on Days 0, 147 and 63 and UA on Days 63 and 360. The third class was made up exclusively of individual samples from modality ACD from 0 to 63 days and on Day 260 (23 individual samples). Finally, the fourth group comprised two subsets: 14 UA individual samples from 0 to 28 days and 19 BCS individual samples from 7 to 28 days. This classification appeared more random than that obtained for Andosol. However, for the Andosol, it was also possible to identify the main response phases; a short-term phase (ST) ranging from 0 to 63 days, a medium-term phase (MT) from Days 98–147 days and a long-term phase (LT) after360 days.

### Influence of biochar and activated carbon on soil fertility parameters

In the following section of this article, we focused on the variables whose combined contributions (to the dimensions retained for the PCA) were predominant for each soil (i.e., those whose contribution exceeded the threshold established if all variables had a contribution equal to this dimension, indicated by a red dotted line in S11 Fig and S12 Fig), as well as on the physical fertility parameter and structural stability, which could not be included in the PCA and CAH analyses.

### Impact of carbonaceous matrices on the physical fertility of West Indies soils

The carbon matrices tested in this study (BCS and ACD) did not affect the structural stability of the Andosol or the Nitisol. In fact, no significant difference was observed in the weighted mean diameter (WMD) obtained between modalities and on different dates (S13 Fig). The values also stabilized over time, with mean values ranging from 2.85 to 3 mm for Andosol and from 2.80 to 2.90 mm for Nitisol.

### Impact of carbonaceous matrices on the chemical fertility of West Indies soils

**Nutrient concentration.** The nutrient concentration in the Andosol remained relatively stable under the control (UA) treatment, with the exception of Na, the content of which decreased threefold after 360 days compared with the average value at the other times points, and P, the content of which increased 1.5-fold at 360 days. Notably the trends observed for the UA modality were also noted in the other two modalities, albeit in different proportions. For all the nutrients listed in **Table 3**, no significant differences were found between the ACD and UA modalities at any time point (S4 Table) or phase. The different results obtained with BCS varied depending on the nutrient considered. For Na and Mg, significant increases were observed throughout the experiment. In the short term (Days 0, 7 and 14) and medium term (Days 28, 63, 98 and 147), increases of 3.8 to 4-fold for Na and 1.4-1.5-fold for Mg were observed compared with UA. In the long term (Day 360), the Mg content increased in similar manner, with a 1.8-fold increase, while Na increased in concentration 8-fold with BCS. For P and K, significant differences appeared at the end of the experiment only, at 147 and 360 days for K and at 360 days for P. Thus, in the long term, a 9% increase in K and a 10% decrease in P were observed. Finally, no significant difference was observed in the Ca content among the three modalities throughout the experiment.

**Table 3:** Impact of *Sargassum* spp. biochar and activated carbon amendment on nutrients and phytoavailable trace elements in Andosol.

Phase	Modality	CEC element				P Olsen	Plant-Available trace elements			
		cmol^ + ^.kg^-1^ dry soil				mg.kg^-1^ dry soil				
		Na	Mg	K	Ca	P	Ni	Cu	Zn	Cd
ST_A_	UA	0.05 ± 0.00 ^b^	1.24 ± 0.03^b^	0.97 ± 0.02^a^	26.1 ± 0.40^a^	36.6 ± 0.88^a^	0.07 ± 0.00 ^b^	7.77 ± 0.09^a^	6.12 ± 0.12^a^	0.139 ± 0.002^a^
	BCS	0.19 ± 0.00 ^a^	1.70 ± 0.03^a^	1.00 ± 0.02^a^	25.2 ± 0.50^a^	35.6 ± 0.53^a^	0.10 ± 0.00 ^a^	7.29 ± 0.11^b^	6.41 ± 0.17^a^	0.132 ± 0.003^ab^
	ACD	0.06 ± 0.00 ^b^	1.27 ± 0.04^b^	1.04 ± 0.04^a^	26.8 ± 0.67^a^	36.6 ± 0.89^a^	0.07 ± 0.00 ^b^	6.90 ± 0.15^b^	5.57 ± 0.09^b^	0.129 ± 0.002^b^
MT_A_	UA	0.06 ± 0.00 ^b^	1.35 ± 0.04^b^	1.12 ± 0.04^a^	27.9 ± 0.56^a^	37.1 ± 0.47^a^	0.08 ± 0.00 ^b^	7.75 ± 0.31^a^	6.03 ± 0.17^a^	0.143 ± 0.004^a^
	BCS	0.24 ± 0.02 ^a^	2.01 ± 0.09^a^	1.23 ± 0.05^a^	28.6 ± 0.57^a^	36.1 ± 0.65^a^	0.09 ± 0.00 ^a^	7.12 ± 0.20^ab^	6.13 ± 0.12^a^	0.132 ± 0.002^a^
	ACD	0.07 ± 0.01 ^b^	1.32 ± 0.05^b^	1.13 ± 0.05^a^	27.2 ± 0.76^a^	38.8 ± 0.54^a^	0.06 ± 0.00 ^c^	6.75 ± 0.20^b^	5.38 ± 0.15^b^	0.138 ± 0.010^a^
LT_A_	UA	0.02 ± 0.00 ^b^	1.35 ± 0.03^b^	1.07 ± 0.03^b^	29.3 ± 1.10^a^	52.5 ± 0.54^a^	0.06 ± 0.00 ^b^	6.81 ± 0.31^a^	7.79 ± 0.49^a^	0.132 ± 0.006^a^
	BCS	0.16 ± 0.01 ^a^	2.42 ± 0.04^a^	1.17 ± 0.00^a^	29.4 ± 0.81^a^	47.0 ± 0.80^b^	0.09 ± 0.00 ^a^	5.50 ± 0.13^b^	7.50 ± 0.36^a^	0.119 ± 0.003^a^
	ACD	0.02 ± 0.00 ^b^	1.41 ± 0.02^b^	1.13 ± 0.01^ab^	28.6 ± 0.47^a^	54.5 ± 0.86^a^	0.06 ± 0.00 ^b^	5.41 ± 0.21^b^	6.18 ± 0.24^a^	0.121 ± 0.004^a^

UA: unamended soil. BCS: Biochar of *Sargassum* spp.. ACD: Activated Carbon DARCO®. ST_A_: Short-term for Andosol (days 0, 7 and 14), MT_A_: Medium-term for Andosol (days 28, 63, 98 and 147), LT_A_: Long term for Andosol (day 360). Values correspond to the mean ± SE (n = 4). Mean values with different superscripted letters for the same phase are statistically different (p-value < 0.05) between modalities (ANOVA test).

In Nitisol, the UA modality resulted in slight increases in nutrient concentration (15–30), a trend also observed for the two other modalities on Days 98 and 147, apart from Na. For this element, the increases were more pronounced, almost doubling at 98 days (S5 Table). The presence of BCS in the soil significantly increased the Na concentration throughout the experiment. A twofold increase was measured in the short term (Days 0, 7, 14, 28 and 63) and medium term (Days 98 and 147) and a 1.4-fold increase was detected in the long term (Table 4.). Similarly, for Mg, significant increases were observed for BCS on every measurement date except at 147 days. Finally, when the data were grouped by phase, the Mg concentration increased by 14% in the short term and by 27% in the long term but not differ in the medium term. BCS did not have an observable impact on K and Ca. ACD, on the other hand, led to reductions of 5–10%, in the short and long term for Mg, in the short and medium term for K and in the medium and long term for Ca.

**Table 4:** Impact of *Sargassum* spp. biochar and activated carbon amendment on nutrients and phytoavailable trace elements in Nitisol.

Phase	Modality	CEC elements				Plant-available trace elements		
		cmol^ + ^.kg^-1^ dry soil				mg.kg^-1^ dry soil		
		Na	Mg	K	Ca	Zn	Pb	As
ST_N_	UA	0.18 ± 0.00^b^	4.43 ± 0.05^b^	1.73 ± 0.02^a^	19.5 ± 0.21^a^	30.0 ± 0.87^ab^	2.39 ± 0.19^a^	0.19 ± 0.01^a^
	BCS	0.37 ± 0.01^a^	5.03 ± 0.08^a^	1.78 ± 0.02^a^	19.7 ± 0.18^a^	32.1 ± 1.00^a^	2.50 ± 0.06^a^	0.19 ± 0.00^a^
	ACD	0.20 ± 0.01^b^	4.22 ± 0.05^c^	1.65 ± 0.02^b^	18.9 ± 0.34^a^	27.6 ± 0.80^b^	2.35 ± 0.05^a^	0.17 ± 0.00^a^
MT_N_	UA	0.42 ± 0.02^b^	5.86 ± 0.19^a^	2.30 ± 0.07^a^	23.4 ± 0.67^a^	22.1 ± 2.58^a^	1.76 ± 0.47^a^	0.18 ± 0.04^a^
	BCS	0.60 ± 0.01^a^	6.35 ± 0.08^a^	2.21 ± 0.03^ab^	22.6 ± 0.21^ab^	26.1 ± 1.69^a^	2.09 ± 0.36^a^	0.21 ± 0.02^a^
	ACD	0.41 ± 0.01^b^	5.27 ± 0.18^b^	2.08 ± 0.07^b^	21.1 ± 0.66^b^	25.1 ± 1.67^a^	2.62 ± 0.13^a^	0.21 ± 0.01^a^
LT_N_	UA	0.21 ± 0.01^b^	4.72 ± 0.06^b^	1.76 ± 0.03^ab^	20.0 ± 0.36^ab^	32.5 ± 6.61^a^	2.25 ± 0.96^a^	0.16 ± 0.04^a^
	BCS	0.43 ± 0.01^a^	5.97 ± 0.14^a^	1.86 ± 0.02^a^	20.8 ± 0.33^a^	30.9 ± 2.41^a^	2.56 ± 0.18^a^	0.21 ± 0.02^a^
	ACD	0.24 ± 0.01^b^	4.52 ± 0.06^b^	1.70 ± 0.03^b^	19.1 ± 0.37^b^	31.1 ± 4.85^a^	2.80 ± 0.29^a^	±0.20 ± 0.01^a^

UA: unamended soil. BCS: Biochar of *Sargassum* spp.. ACD: Activated Carbon DARCO®. ST_N_: Short-term (days 0, 7, 14, 28 and 63), MT_N_: Medium-term (days 98 and 147), LT_N_: Long term (day 360). The values correspond to the mean ± SE (n = 4). Mean values with different superscripted letters for the same phase are statistically different (p-value < 0.05) between modalities (ANOVA test).

**Plant-available trace elements.** For the Andosol, the trace element concentration of As, Cu, Fe and Cd remained stable throughout the experiment kinetics with the UA modality, whereas the Ni concentration decreased after 147 days for all the modalities tested (S4 Table). The effect of the carbon matrix on trace element availability varied over time (S4 Table). However, we found that the presence of BCS in the soil tended to significantly increase Ni availability: in the short term by 37%, in the medium term by 22% and in the long term by 34% (**Table 3**). Significant decreases in Cu availability were also detected in the short term (5%) and long term (19%). BCS had not detectable impact on Zn and Cd availability. However, the significant effects observed with ACD consistently involved reductions in trace element availability. For example, a decrease on the order of 10–20% was observed for Cu, percentages that are statistically similar to those obtained with BCS. Reductions in Zn availability of 11% and 19% in the short term and medium term, respectively, were also measured. The reduction in Ni availability was significant only in the medium term (16%).

For Nitisol, the Cu and Cd concentrations increased by 20–30% on Days 98, 147 and 360, whereas the Zn concentration decreased by 20–30% for the UA modality (S5 Table). BCS had a significant effect on As availability on Day 14 only, with no differences detected on any of the other days or with any of the other phases (Table 4). BCS also had no effect on Pb, Fe or Zn concentrations (S5 Table). For the other trace elements, significant short-term (24%) and medium-term (35%) increases were measured for Ni, but there was no difference in the long term. BCS also reduced the Cu concentration by 13% in the short term (but not the MT or LT) and the Cd concentration in the short term (−14%) and long term (−27%). In the case of ACD, the significant effects observed consistently involved reductions in trace element availability. Decreases in the concentration were observed on Days 0 and 14, for Pb, and on Days 0,14 and 28 for As. However, in the phase analysis, no further significant differences were detected in the short, medium or long term (**Table 4**). As with BCS, ACD had no effect on Zn, Ni or Fe concentrations, except on Day 14 for Fe. Short-term decreases were also detected for Cu (27%) and for Cd (11%) (S5 Table).

**Fig 4:**
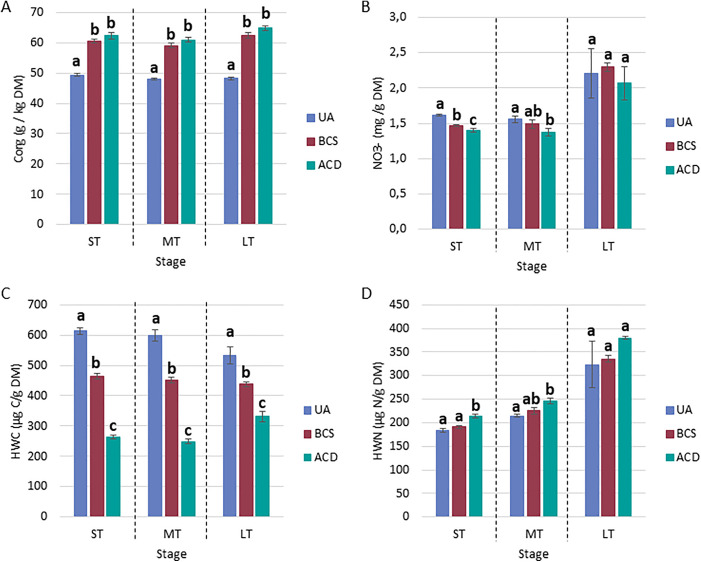
Impact of *Sargassum* spp. biochar and activated carbon amendment on the carbon and nitrogen contents of Andosol. UA: unamended soil. BCS: Biochar of *Sargassum* spp.. ACD: Activated Carbon DARCO®. ST: Short-term (Days 0, 7 and 14), MT: Medium-term (Days 28, 63, 98 and 147), LT: Long term (Day 360). The values correspond to the mean ± SE (n = 4). Mean values with different superscripted letters for the same phase are statistically different (p-value < 0.05) between modalities (ANOVA test).

**Carbon and nitrogen contents.** For the Andosol, the C_org_ and HWC (labile carbon fraction) contents in the control (UA) were stable over time, but both carbon matrices induced significant increases in the soil C_org_ content. These increases (approximately 27%) were similar between BCS and ACD (**Fig 4A**). Moreover, these amendments resulted in decreases in the labile carbon fraction (**Fig 4C**): by 25% (ST and MT) and 20% (LT) for BCS and by 60% (ST and MT) and by 40% (LT) for ACD. With respect to N, at 360 days, the UA NO_3_^-^ concentration increased by 40% (compared with that on the other tested days), and the HWN (labile nitrogen fraction) increased by 57%. These increases were also observed for the two other modalities. No significant effect on the nitrogen labile fraction (HWN) was detected with BCS, but a 15% increase was measured for ACD in the short term and the medium term (**Fig 4D**). There was an effect on nitrates in the short-term with an average reduction of 10% with BCS. For ACD, this effect continued in the medium term, with an average reduction of 12% (**Fig 4B**).

**Fig 5:**
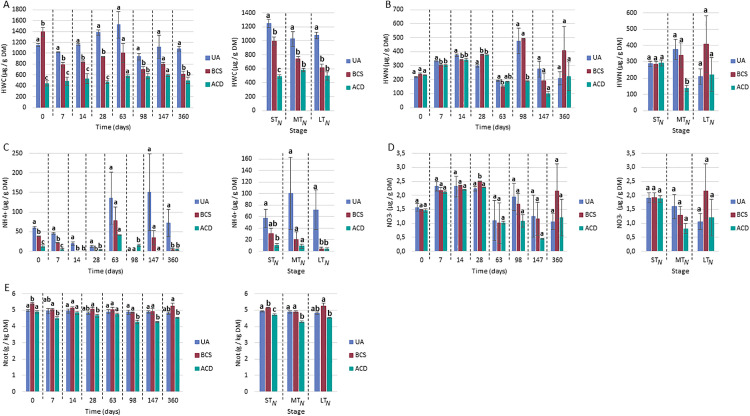
Impact of *Sargassum* spp. biochar and activated carbon amendment on carbon and nitrogen contents in Nitisol. UA: unamended soil. BCS: Biochar of *Sargassum* spp.. ACD: Activated Carbon DARCO®. ST_N_: Short-term (Days 0, 7, 14, 28 and 63), MT_N_: Medium-term (Days 98 and 147), LT_N_: Long term (Day 360). The values correspond to the mean ± SE (n = 4). Mean values with different superscripted letters for the same phase are statistically different (p-value < 0.05) between modalities (ANOVA test).

For Nitisol, in contrast to Andosol, greater variability in the nitrogen profile was observed over time, leading to considerable uncertainty when the data were grouped by time phase (**Fig 5**). UA value monitoring revealed (i) a gradual increase in the nitrogen labile fraction (HWN)from Day 0 (≈230 µg.g^-1^ DM) to Day 28 (≈300 µg.g^-1^ DM), followed by a sharp decrease on Day 63 (150−190 µg.g^-1^ DM) and greater for the remainder of the experiment;(ii) a decrease in the NO_3_^-^ concentration after Day 28 (with high variability in the responses); and (iii) a decrease in the NH_4_^+^ concentration from Day 0–28, followed by peak concentrations on Days 63 and 147 days. With respect to the carbon labile fraction (HWC) and total N content, the UA results were stable over time, at approximately 1200 µg.g^-1^ DM and 4.8–5 g.kg^-1^ DM, respectively.

BCS reduced the labile carbon fraction (HWC) throughout the course of the study by 15%, 28% and 43% in the short, medium and long term, respectively (**Fig 5**). For HWN, differences were observed on Days 7, 14, 28 and 63, with alternating trends between increasing and decreasing. These observations indicate the absence of statistically significant effect in the short, medium and long term. For NH_4_^+^, no significant differences were observed in the short or medium term, because of the high variability of the values. However, significant decreases of up to 100% were observed throughout the study (except on Days 63 and 147). BCS did not seem to have any effect on NO_3_^-^ levels; the only difference observed was on Day 28 (+12%). With respect to the total N concentration, only BCS had a short-term effect (4% increase). Decrease in the HWC concentrations of 58% (short term), 43% (medium term) and 54% (long term) were noted for ACD. With respect to the HWN concentration, only a strong decrease of 64% was observed in the medium term with ACD amendment. NH_4_^+^ and total N concentrations also decreased; approximately90% for NH_4_^+^ and 7% for N_tot_. Finally, no differences in NO_3_^-^ content were observed during the experiment.

**pH**. Among the West Indies soils tested, only Nitisol showed significant decreases in pH (0.12–0.16). These small decreases in pH were observed with the BCS amendment on Days 0, 7 and 28 but did not persist over time and were no longer detectable when analysed by phase (S14 Fig). The same was true for ACD, with which the pH decreased on Days 7 (−0.11) and 28 (−0,15) but increased on Day 14 (+0.14).

### Impact of the carbonaceous matrix on Andosol biological fertility

Significant decreases in enzyme activity were measured in all groups at the end of the experiment (at 147 and 360 days), with an average twofold decrease for β-glucosidase, xylosidase and arylsulfatase. This trend was less pronounced for leucine-aminopeptidase activity. Without considering the data from the last time point, the average enzyme activities of the Andosol UA samples were between 100 and 150 µg.g^-1^ DM.h^-1^ for β-glucosidase and xylosidase, between 300 and 400 µg.g^-1^ DM.h^-1^ for arylsulfatase and between 130 and 180 µg.g^-1^ DM.h^-1^ for leucine-aminopeptidase.

**Fig 6:**
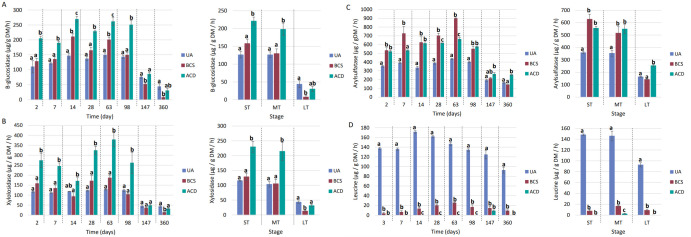
Enzymatic activities of (A) β-glucosidase, (B) β-xylosidase, (C) arylsulfatase and (C) leucine aminopeptidases in Andosol. UA: unamended soil. BCS: Biochar of *Sargassum* spp*.*. ACD: Activated Carbon DARCO®. ST: Short-term (Days 0, 7 and 14), MT: Medium-term (Days 28, 63, 98 and 147), LT: Long term (Day 360). DM: soil dry matter. The values correspond to the mean ± SE (n = 4). Mean values with different superscripted letters for the same day are statistically different (p-value < 0.05) between modalities (ANOVA test).

With respect to β-glucosidase activity (**Fig 6A**), significant differences between the control and BCS were observed only on Days 14, 63,147 and 360. On Days 14 and 63, a 30−40% increase was measured in the presence of BCS. At the end of the experiment, a decrease in activity with BCS measured (−30% on Day 147 and −80% on Day 360). In contrast, in the presence of ACD, there was a significant increase in β-glucosidase activity on every test date except Days 147 and 360, when the activity was equal to that of the control. Upon grouping by study phase, in the short term, no significant difference was observed between the BCS and UA modalities, despite a 25% increase in activity in the presence of the BC. The ACD modality significantly differs from the other two modalities, with a 75% increase in β-glucosidase activity compared with the UA (126 ± 8.92 µg.g^-1^ DM.h-^1^). These same trends were observed in the medium term. In the long term, compared with UA (43.7 ± 9.91 µg.g^-1^ DM.h^-1^), BCS resulted in a significant 80% reduction in enzyme activity.

For xylosidase (**Fig 6B**), no significant difference was measured between UA and BCS, except at 360 days, when the presence of BCS reduced the enzyme activity by approximately 70%. Compared with xylosidase activity for UA individual samples, activity in the presence of ACD was significantly greater, except on Days 14, 147 and 360. On these days, xylosidase activity was similar between the two modalities. In the short and medium term, no significant difference was observed between BCS and UA but compared with UA (ST:116 ± 1.58 µg.g^-1^ DM.h^-1^, MT: 103 ± 8.99 µg.g^-1^ DM.h^-1^), ACD exhibited an order for magnitude (100%) increase in xylosidase activity. In the long term, compared with UA (43.3 ± 3.94 µg.g^-1^ DM.h^-1^), BCS exhibited a significant 27% decrease in activity, but the xylosidase activity did not differ between ACD and UA.

For arylsulfatase (**Fig 6C**), BCS amendment resulted in a significant increase in enzyme activity over time, except at the end of the study (Days 147 and 360). ACD also increased activity, except on Day 7. Among the two carbonaceous matrices, higher activity was observed with BCS on Days 7 (+36%), 28 (+13%) and 63 (+36%). However, at the end of the experiment, ACD showed the highest arylsulfatase activity. These variations become less pronounced when the dates were grouped together by study period. In the short and medium term compared with UA (ST: 359 ± 9.22 µg.g^-1^ DM.h^-1^, MT: 353 ± 24.9 µg.g^-1^ DM.h^-1^), the carbonaceous matrices significantly increased arylsulfatase activity by 60% for BCS and 55% for ACD. In the long term, only the ACD modality was significantly different from BCS and UA (163 ± 4.38 µg.g^-1^ DM.h^-1^), with a 55% increase in enzyme activity.

For leucine-aminopeptidase (**Fig 6D**), compared with the UA modality, the presence of a carbonaceous matrix drastically reduces the activity of the enzyme. ACD had the strongest effect, with no measurable enzyme activity, except on Day 147. In the short and long term, both matrices had equal effects on leucine-aminopeptidase activity, with values of zero or close to zero. In the medium term, there was a significant difference between the two matrices with a measured activity of 16.5 ± 1.55 µg.g^-1^ DM.h^-1^ for BCS and 2.35 ± 1.15 µg.g^-1^ DM.h^-1^ for ACD.

### Impact of the carbonaceous matrix on Nitisol biological fertility

For Nitisol, UA enzyme activities averaged between 40 and 100 µg.g^-1^ DM.h^-1^ for β-glucosidase, 30 and 50 µg.g^-1^ DM.h^-1^ for xylosidase, 50 and 80 µg.g^-1^DM.h^-1^ for arylsulfatase and between 70 and 140 µg.g^-1^ DM.h^-1^ for leucine-aminopeptidase.

**Fig 7:**
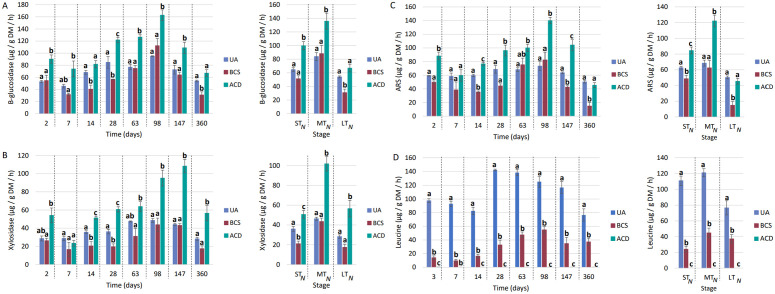
Nitisol enzymatic activities (A)β-glucosidase, (B) β-Xylosidase, (C) Arylsulfatase and (C) Leucine aminopeptidases. UA: unamended soil. BCS: Biochar of *Sargassum* spp.. ACD: Activated Carbon DARCO®. ST_N_: Short-term Nitisol (Days 0, 7, 14, 28 and 63), MT_N_: Medium-term Nitisol (Days 98 and 147), LT_N_: Long term Nitisol (Day 360). DM: soil dry matter. The values correspond to the mean ± SE (n = 4). Mean values with different superscripted letters for the same day are statistically different (p-value < 0.05) between modalities (ANOVA test).

For β-glucosidase activity (**Fig 7A**), significant differences between control and BCS were observed only on Days 14, 28 and 360. On these days, a 30−40% decrease in activity was measured in the presence of BCS. In the presence of ACD, a significant increase in activity was observed on every test date, with the exception of Day 360, when the activity was equals to that of UA. In the short- and medium-term no significant differences were observed between the BCS and UA treatment, but compared with UA (ST: 64.8 ± 2.78 µg.g^-1^ DM.h^-1^, MT: 83.9 ± 5.77 µg.g^-1^ DM.h^-1^), ACD increased the enzyme activity by approximately 55–60.In the long term, compared with UA (43.7 ± 9.91 µg.g^-1^ DM.h^-1^) BCS resulted in a significant 40% reduction in enzyme activity.

For xylosidase activity (**Fig 7B**), no significant difference between UA and BCS was detected, except on Days 14 and 28, when the presence of BCS reduced the enzyme activity by approximately 40%. With ACD, the enzyme activities were significantly higher than those with UA, except on Day 7. In the short term, compared to the UA modality (36.0 ± 2.41 µg.g^-1^ DM.h^-1^), BCS significantly reduced enzyme activity by 40%, whereas ACD increased activity by 40%. In the medium term, no differences between the BCS and UA modalities were visible, and ACD resulted in a 120% increase in xylosidase activity compared with the UA (46.3 ± 1.55 µg.g^-1^ DM.h^-1^). The same trend was observed in the long term, with a 99% increase in enzyme activity detected with ACD compared with the UA (28.3 ± 1.71 µg.g^-1^ DM.h^-1^).

For arylsulfatase activity (**Fig 7C**), no significant difference between UA and BCS was observed, except at Days 14, 147 and 360, on which decreases in enzyme activity of 40% for the first two dates and 70% at 360 days were recorded. For the ACD modality, increases in arylsulfatase activity were measured except for Day 7, when the enzyme activity was similar to that of UA. In the short term, compared to UA (62.7 ± 1.84 µg.g^-1^ DM.h^-1^), BCS induced a significant 22% decrease in enzyme activity. On the over hand, ACD induced a significant 38% increase in activity compared with the UA. In the medium term, the difference between the BCS and UA modalities was no longer significant, but ACD resulted in a 78% increase compared with the UA (68.5 ± 3.81 µg.g^-1^ DM.h^-1^). In the long term, BCS treatment caused a 70% decrease in arylsulfatase activity, and the activity with ACD and UA (50.3 ± 1.85 µg.g^-1^ DM.h^-1^) did not significantly differ.

For leucine-aminopeptidase activity (**Fig 7D**), as was observed for the Andosol, enzyme activity was not detectable with ACD, and leucine-aminopeptidase activity in the presence of BCS was greatly reduced compared with the UA. More precisely, compared with the UA (ST: 110 ± 5.84 µg.g^-1^ DM.h^-1^, MT: 121 ± 5.13 µg.g^-1^ DM.h^-1^, LT: 76.6 ± 8.98 µg.g^-1^ DM.h^-1^), BCS led to decreases of 78%, 63% and 51% in the short, medium and long term, respectively.

### Effects of *Sargassum* spp. biochar and activated carbon on the relationship among soil fertility parameters

In addition to affecting soil fertility parameters (biological, chemical and physical), the addition of an amendment may also affect the relationships among fertility parameters. In this study, the carbon matrices used did indeed modify the existing relationships among the fertility parameters for both soils (S15 Fig). The impact of BCS and ACD ranged from attenuating existing positive correlations to strengthening negative correlations. Although the results obtained for the two soils differed, for numerous correlations, more pronounced effects were observed with ACD than with BCS. For instance, the positive correlations between the activities of leucine aminopeptidase and the other enzymes in the UA modality were weakened in the presence of BCS and became negative in the case of ACD in both soils (S15 Fig). Differences in behaviours of the two soils were also detected, with negative correlations in the Andosol between nutrients (Ca, K, and Mg) and C_org_ or N becoming attenuated with BCS and strongly negative with ACD. Conversely, in Nitisol, these correlations were initially positive, became neutral to negative with BCS, and strongly negative with ACD.

## Discussion

The objective of this study was to assess the effects of BCS and ACD on the fertility of contaminated West Indies soils at the amendment rate recommended for CLD sequestration. This was achieved by investigating the effects of these matrices on soil fertility parameters. The results showed that the application of these carbonaceous matrices significantly affected the fertility of these soils. However, the extent and nature of these effects varied according to the soil properties, matrix properties, and experimental phase.

### Validation of the sequestration properties of the carbonaceous matrices

In this study, the performance of BCS varied according to the type of soil amended. In the Andosol, BCS reduced the environmental availability of CLD by 50–60%, whereas in the Nitisol, the reduction was much lower, reaching only 15–20%. This significant difference is probably associated with the high initial CLD contamination in Nitisol (7.99 mg.kg-¹ of dry soil) compared with that in Andosol (1.85 mg.kg^-1^ of dry soil). At the Nitisol level of contamination, the properties of BCS and the conditions of application (particle size and dose) are not conductive to achieve the extent of sequestration previously reported with this type of BC in other West Indies soils [33,47]. While a 50–60% reduction in the environmental availability of CLD in soils would halve exposure and thus the risk associated with this pesticide, particularly overexposure, the time required to reach this threshold remains very long (between 147 and 360 days). This observation calls into question the effectiveness of this remediation strategy for heavily contaminated soils. To a lesser extent, this difference in performance between the two soils was also observed with ACD. These results highlight the importance of the initial level of contamination in the sequestration capacity of both BCS and ACD, regardless of the specific experimental conditions.

In the case of Andosol, the observed reductions in the environmental availability of CLD are comparable to those obtained in an earlier study using *Sargassum* spp. BC produced by conventional pyrolysis [33]. The differences between these two studies could be associated with the pyrolysis method used [48]. Although this method does not appear to significantly affect the BC specific surface area (BET) or mesopore volume, the micropore volume was halved during microwave pyrolysis. This reduction in microporosity could explain the decrease in sequestration observed in this study. However, given that the pore volume remains high compared with that of the conventional range of BCs [49] and that its cage structure is composed of many chlorine atoms, the size of CLD slightly exceeds that of the micropores (<2 nm), which does not have a drastic effect on its sequestration [50]. It is also possible that microwave pyrolysis, owing to its specific heating properties, altered the porosity distribution in the material [48].

### Carbon matrix-induced changes in West Indies soil parameters depending on soil properties

The PCAs and HAC analyses performed for each soil type yielded contrasting results depending on the soil type. For Andosol, a clear distinction was observed between the control and BCS treatments, whereas for Nitisol, this distinction was much less marked, suggesting that BC had less of an impact on soil fertility. Thus, in contrast to our initial hypothesis, it appears that the effects of BCS under bioremediation conditions depend heavily on the initial properties of each soil. Although the two soils tested in this study have common origins and characteristics (volcanic type, tropical climate, clayey), the PCA results reveal a marked difference in initial fertility between these two soils. In particular, compared with Nitisol, Andosol is characterized by significantly greater biological fertility, as evidenced by higher β-glucosidase, β-xylosidase and arylsulfatase enzyme activities. This contrast between the two soils can be explained by the slightly higher initial C/N ratio, pH and OM content of the Andosol (**Table 1**), which favour microbial activity. In addition, the level of CLD contamination in Nitisol is much higher (4 times), which may also explain the low enzyme activity owing to its possible negative impact on the microbial communities of this soil [51]. However, the chemical fertility of Nitisol is much greater than that of Andosol owing to its higher concentrations of nutrients, notably ammonium and labile carbon, than those measured in the Andosol. Such chemical enrichment is related to the weathering of the clay minerals in the parent rocks, which allows the release of large quantities of nutrients and the presence of halloysite clays with a high cation exchange capacity, which allows for efficient retention of these nutrients [39,52].

PCA revealed differences in the responses to carbon matrices (BCS and ACD), mainly in terms of intensity; however, some indicators showed very different values depending on soil type. For BCS, (i) arylsulfatase activity was stimulated in Andosol but reduced in Nitisol; (ii) the ammonium concentrations increased strongly in the medium and long term in Andosol but decreased in Nitisol; and (iii) the cadmium concentrations decreased only in Nitisol. For ACD, the differences included (i) the magnesium, potassium and calcium contents, which were unaffected in the Andosol but decreased in the Nitisol; (ii) the labile nitrogen content, which increased in the Andosol but decreased in the Nitisol; (iii) the nitrate content, which was strongly reduced in the Nitisol, but remained stable in the Andosol, (iv) the ammonium concentration, which was unaffected in the Andosol but strongly decreased in the Nitisol. These observations highlight the influence of initial soil properties on the effect of carbon matrices on soil fertility. However, these effects were less pronounced those that reported by [53]. This difference between the two studies can be explained by the much less marked differences between the two soils in our study, which are typical West Indies clay soils.

### Carbon matrix-induced changes in West Indies soil parameters depending on BC and AC properties

The PCAs and HAC analyses carried out highlighted a clear distinction between soils amended with BCS and those amended with ACD. These differences may be related to the physical and chemical properties of the carbon matrices, which are strongly influenced by the characteristics of the raw materials used by production conditions such as temperature and duration of pyrolysis, or the presence and type of activation [21,54,55]. These properties are known to modulate their fertilizing potential. In particular, the chemical characteristics of the matrices, combined with their textural properties, seem to play decisive roles in their impact on soil fertility. More specifically, our study highlighted variable responses on different pillars of fertility that are linked to both the properties of the matrices and the characteristics of the soils tested. In terms of physical fertility (aggregate stability and water retention), the porosity and hydrophobicity of the matrices are the main parameters affecting its improvement [3]. However, no effect on physical fertility was observed in this study, because of the high initial stability of the soils (both clayey). From a chemical point of view, the two matrices tested had different effects on the soils. In particular, BCS increased the availability of sodium and magnesium in the soil, whereas ACD reduced their availability or had no effect. These increases with BCS are related to the composition of the raw material. *Sargassum* spp. biomass is rich in nutrients [56,57]. Upon contacting the soil, the sodium and magnesium contained in the BCS are released into the soil solution, resulting in an increase in the concentration of these two elements. Similarly, BCS increased the nickel content in both soils, because of its high concentration in *Sargassum* spp. [58,59]. In contrast, ACD tended to reduce TME availability in both soils by an average of 10–20%. This trend may be due to the activation process, which can modify surface reactivity to improve bonding between the TMEs and BC, but also, to the higher pyrolysis temperature, which may allow greater porosity development than with the BCS [60].

In terms of biological fertility, the two matrices induced contrasting responses to enzymes involved in the carbon cycle. In both soils, BC had a neutral to inhibitory effect on the activities of the enzymes β-glucosidase and β-xylosidase, which is in line with the findings of previous studies [24,61]. These decreases can be attributed to different factors including disruption of enzyme operating conditions (alteration of the enzyme active site), the presence of TMEs in the BCS, which can be inhibitory [62], and the limitation of microbial growth induced by unfavourable stoichiometric conditions (high proportions of aromatic C and high C/N ratios) as well as the sorption of enzymes by BCs [24]. In contrast, ACD significantly increased carbon-related enzyme activity. This result, which is consistent with the work of Ameur et al., (2018), could be explained by a higher and/or more accessible fraction of labile carbon, or by the presence of small molecules released by ACD, that act as allosteric regulators of the enzymes.

### Impact of time on the carbon matrix effect

The PCAs and HAC analyses revealed similar temporal effects among the three modalities studied (UA, BCS and ACD). These dynamics seemed to be related mainly to the natural evolution of soils under laboratory incubation conditions, which caused changes in their physico-chemical and biological properties. For example, a strong decrease in carbon-related enzyme activity was observed (especially in the Andosol) as a result of the reduction in resources during the incubation period. As this experiment was carried out under controlled conditions in a closed microcosm without integrating plants into the system, there was no loss of nutrients through leaching or root uptake, nor was there any input of carbon sources through exudates. Therefore, the temporal effects described by Joseph et al., (2021) related to the interactions among BC, soil and plants in the context of the annual crop cycle were not observed in this study.

The BCS showed overall conservation of the effects observed across the three temporal phases of the study, suggesting a certain stability in West Indies soils. However, lack of plants in the system, and therefore of interactions among roots, BCS and soil that could modify the BC [12,63], represents a potential limitation of this conclusion. On the other hand, for the ACD, a loss of efficiency was observed, especially with regard to the sequestration of TMEs, in the long term in the Andosol and in the medium and long term in the Nitisol. These results support the hypothesis of competition for ACD sorption sites between CLD and soil TMEs.

### Agronomic value of *Sargassum* spp. biochar on West Indies soils

From an agronomic point of view, the application of BCS seemed to improve the chemical fertility of soils, particularly in terms of the some nutrient concentrations (Na and Mg). Although the effects of BCs on soil chemical properties have been shown to be variable and related mainly to indirect changes in soil properties [7], the effects observed with BCS seemed to result primarily from a direct contribution of the matrix to the nutrient stock. This has already been observed with BC derived from animal raw material, which is known to be rich in nutrients (notably P and N) [64] and *Sargassum* spp.-derived BC has high concentrations of macronutrients such as Na, Mg and K. However, the total nutrient content of a BC does not necessarily reflect the actual release of these nutrients once the BC is applied to the soil [65]. Although some total concentrations of nutrients were high in BCS, they did not always have a direct effect on soil chemistry. This suggests that for some elements, such as K, the proportion of readily available (water-extractable and exchangeable) forms in BCS remained small, whereas for others (such as Na and Mg) the opposite was true.

The effects of BCS on biological soil fertility were more variable and require further analysis. The inhibitory effect observed on the enzyme activity of leucine-aminopeptidase raises important questions about the nitrogen cycle in West Indies soils. Indeed, a drastic reduction in leucine aminopeptidase (LAP) activity would have major consequences on the dynamics of organic nitrogen in soils. By severely limiting the depolymerization of proteins and peptides, the release of amino acids would be reduced. As a result, the flow of organic nitrogen in available mineral forms (NH₄⁺ and NO₃⁻) slows, leading to a decrease in the availability of assimilable nitrogen for microorganisms and plants [66–68]. These changes could alter the composition and function of soil microbial communities, favoring organisms capable of exploiting other nitrogen sources at the expense of specialized organisms [69]. Finally, these disturbances could indirectly affect the carbon cycle by creating an imbalance in the stoichiometric ratios of enzymatic activities (C:N:P) and influencing the mineralization of organic matter as a whole [67]. Although this enzyme was strongly affected by the presence of BCS, other enzymes involved in the nitrogen cycle (N-acetyl-β-glucosaminidase, protease and urease) should be studied. This would make it possible to determine whether the overall effect of BCS on the nitrogen cycle is positive, as suggested by various meta-analyses [24,61], or negative, such as the results specific to leucine aminopeptidase. It is also possible that the effect observed on leucine-aminopeptidase was influenced by the analytical method employed. Indeed, interactions between carbonaceous matrices and enzyme substrates or products in current methods can distort results [70]. These interactions differ depending on the method used and the type of enzyme studied [23]. However, if the results obtained in our study do not reflect a methodological limitation related to the presence of biochar but rather enzyme inhibition, it would be possible to combine the use of biochar with that of nitrogen-rich organic fertilizers to stimulate microbial communities and mitigate the effects of BCS [71].

## Conclusion

The initial results confirmed that *Sargassum* spp. biochar (BCS), although initially designed for the bioremediation of CLD-contaminated soils in the West Indies, had a significant effect on the fertility of these soils, with effects that varied according to their specific properties. Although the availability of certain elements, such as sodium and magnesium, increased, others, such as potassium and phosphorus, were not significantly affected, despite their initially high concentrations in BCS. Among the trace elements, the concentration of only nickel significantly increases in both soils. In terms of biological aspects, the results differed. BCS had no detrimental effect on enzymes involved in the carbon cycle and even stimulated enzyme activity in some cases, such as that of arylsulfatase in the Andosol. However, a negative effect of BCS on leucine-aminopeptidase activity was observed, raising questions about its potential impact on the nitrogen cycle in soils. As expected, BCS had no significant effect on the physical fertility of the two soils studied.

Further studies, incorporating the role of plants in the experimental design, are necessary to further assess the impact of BCS on soil fertility. Observing and measuring plant-biochar interactions, in terms of both CLD sequestration efficiency, and on soil properties and the growth of Caribbean plants, would provide a more detailed understanding of its potential for use in the Caribbean. Furthermore, experiments conducted under real-world conditions, on a control plot scale, are essential to consider climate variability, and to assess the spatial distribution of biochar particles in the soil profile and its consequences for fertility.

If these further studies confirm conclusive effects, both in terms of bioremediation and improved soil fertility, biochar derived from *Sargassum* spp. could be used in family gardens and market gardening. Larger-scale use, with an aim towards fertilization through integration into fertilizer mixtures, could also be considered but remains limited given the current BCS production capacity. Indeed, between January and March 2023, 200,000 tonnes of seaweed were collected [72], corresponding to a potential production of approximately 8,000 tonnes.year^-1^ *Sargassum* spp. biochar (BCS), enabling the treatment of approximately 250 ha.year^-1^ (with a 2% amendment rate). While this production remains relatively modest, the steady increase in *Sargassum* spp. strandings each year to year could gradually increase BCS production and, consequently, the area of agricultural land treated.

## Supporting information

S1 Table Measurement dates for various fertility indicators during the incubation period. Dates are in days.
(PDF)

S2 Table Analytical method used for each tested indicator. (PDF)

S3 Table Substrates, reaction buffers and incubation times of the enzymes tested via the colorimetric method. (PDF)

S4 Table Mean values (n = 4) of Andosol plant-available trace elements and CEC elements as a function of time. UA: unamended soil. BCS: Biochar of *Sargassum spp.* ACD: Activated Carbon DARCO®. The values correspond to the mean ± SE (n = 4). Mean values with different superscript letters for the same stage (a, b, c) are statistically different (P < 0.05) between modalities (ANOVA test).
(PDF)

S5 Table Mean values (n = 4) of Nitisol plant-available trace elements and CEC elements as a function of time. UA: unamended soil. BCS: Biochar of *Sargassum spp.* ACD: Activated Carbon DARCO®. The values correspond to the mean ± SE (n = 4). Mean values with different superscript letters for the same day (a, b, c) are statistically different (P < 0.05) between modalities (ANOVA test).
(PDF)

S6 Table Data from the study. Sol: soil type (Nitisol or Andosol), moda: modality (BCS, UA, ACD), rep: duplication, TC = HWC, TN = HWN, DE = chlordecone environmental availability, SS = soil structural stability.
(PDF)

S1 Fig Principal component analysis (PCA1) of the soil parameters measured for each soil. **Points represent the coordinates of each sample according to the 2 dimensions of PCA1**. (red: Andosol and blue: Nitisol). TC = HWC, TN = HWN. Confidence ellipses (95%) were plotted for each treatment.
(TIF)

S2 Fig Contribution levels to the first dimension of the variables tested in PCA1. The red line represents the average contribution threshold if all variables had an equal contribution. TC = HWC, TN = HWN.
(TIF)

S3 Fig Contribution levels to the PCA2 first and the second dimensions of the variables tested. The red line represents the average contribution threshold if all the variables had equal contributions. TC = HWC, TN = HWN.
(TIF)

S4 Fig Principal component analysis (PCA2) of the Andosol parameters measured for each incubation date and modality. Points represent the coordinates of each sample according to the 2 dimensions of PCA2. TC = HWC, TN = HWN. Confidence ellipses (95%) were plotted for each treatment.
(TIF)

S5 Fig Hierarchical ascending classification of Andosol (HAC1). BCS: *Sargassum spp.* Biochar, ACD: Activated carbon DARCO®, UA: Unamended soil. Cluster 1: Day 360 (UA, BCS and ACD), Cluster 2: UA (Days 0, 7, 14, 28, 63, 98 and 147), Cluster 3: ACD (Days 0, 7, 14, 28, 63 and 98), Cluster 4: BCS (Days 0, 7, 14, 28, 63, 98 and147) + ACD (Days 147). Yellow indicates the enclave of ACD individual samples on Day147.
(TIF)

S6 Fig Percent contribution of each dimension to the construction of PCA3. (TIF)

S7 Fig Contribution levels of the variables tested to dimensions 1, 2 and 3 of PCA3. The red line represents the average contribution threshold if all the variables had equal contributions. TC = HWC, TN = HWN.
(TIF)

S8 Fig Variable contributions circle for PCA3 in dimensions 1 and 2. TC = HWC, TN = HWN.
(TIF)

S9 Fig Principal component analysis (PCA3) of the Nitisol parameters measured for each incubation date and modality. Points represent the coordinates of each individual sample according to the 2 dimensions of PCA3. TC = HWC, TN = HWN. Confidence ellipses (95%) were plotted for each treatment.
(TIF)

S10 Fig Hierarchical ascending classification of Nitisol. BCS: *Sargassum spp.* Biochar, ACD: Activated carbon DARCO®, T: Unamended soil (UA). Cluster 1: BCS (Day 98) + UA (Days 98 and 147) + ACD (Days 98 and 147), Cluster 2: BCS (Days 0, 63, 147 and 360) + UA (Days 63 and 360) + ACD (Days 360), Cluster 3: ACD (Days 0, 7, 14, 28, 63, 98 and 360), Cluster 4: BCS (Days 7, 14 and 28) + UA (Days 0,7 14 and 28).
(TIF)

S11 Fig Percent contribution of each dimension to the construction of PCA_2 (A) and percent contribution of each parameter to the construction of PCA_2 considering the first 6 dimensions (B). (TIF)

S12 Fig Percent contribution of each dimension to the construction of PCA_3 (A) and percent contribution of each parameter to the construction of PCA_3 considering the first 6 dimensions (B). TN: Labile nitrogen (HWN), TC: Labile carbon (HWC).
(TIF)

S13 Fig Impact of *Sargassum spp.* biochar and activated carbon amendment on (A) Andosol and (B) Nitisol soil structural stability. UA: unamended soil. BCS: Biochar of *Sargassum spp.* ACD: Activated carbon DARCO®. The values correspond to the mean ± SE (n = 4). Mean values with different superscripted letters for the same stage are statistically different (p-value< 0.05) between modalities (ANOVA test).
(TIF)

S14 Fig Impact of *Sargassum spp.* biochar and activated carbon amendment on Nitisol pH (A) over time and (B) by stage. UA: unamended soil. BCS: Biochar of *Sargassum spp.* ACD: Activated carbon DARCO®. ST_N_: Short-term Nitisol (Days 0, 7, 14, 28 and 63), MT_N_: Medium-term Nitisol (Days 98 and 147), LT_N_: Long term Nitisol (Day 360). The values correspond to the mean ± SE (n = 4). Mean values with different superscripted letters for the same day (a, b, c) are statistically different (P < 0.05) between modalities (according to the ANOVA test).
(TIF)

S15 Fig Correlation matrices between the soil fertility indicators and thetested matrices. **(a) Andosol without amendment, (b) Andosol + BCS, (c) Andosol + ACD, (d) Nitisol without amendment, (e) Nitisol + BCS and (f) Nitisol + ACD.** TN = HWN, TC = HW.
(TIF)
