# Supplementary material for: Effects of biochar from algae (Sargassum spp.) on the fertility of two chlordecone contaminated West Indies soil
Source: PLoS One. 2025 Dec 30;20(12):e0338385. doi: 10.1371/journal.pone.0338385 (PMC12753066; doi:10.1371/journal.pone.0338385)
Supplement: S1 Table — Dates are in days. (PDF) [file pone.0338385.s001.pdf]

SUPPLEMENTARY DATAS

S1 Table. Measurement dates for various fertility indicators during the incubation period.

Indicators	0/2	7	14	28	63	98	147	360
pH	X	X	X	X	X	X	X	X
CEC	X	X	X	X	X	X	X	X
Total trace element	X						X	X
Plant-available trace element	X	X	X	X	X	X	X	X
Phosphorus	X	X	X	X	X	X	X	X
C_{tot} / C_{org}	X	X	X	X	X	X	X	X
N_{tot}	X	X	X	X	X	X	X	X
Nitrate / Ammonium	X	X	X	X	X	X	X	X
HWC / HWN	X	X	X	X	X	X	X	X
Structural stability	X			X	X		X	X
Enzymatic Activities	X	X	X	X	X	X	X	X
CLD environmental availability	X		X		X	X	X	X

Dates are in days.
