# Supplementary material for: Effects of biochar from algae (Sargassum spp.) on the fertility of two chlordecone contaminated West Indies soil
Source: PLoS One. 2025 Dec 30;20(12):e0338385. doi: 10.1371/journal.pone.0338385 (PMC12753066; doi:10.1371/journal.pone.0338385)
Supplement: S2 Table — (PDF) [file pone.0338385.s002.pdf]

SUPPLEMENTARY DATAS

S2 Table. Analytical method used for each tested indicator

Class of fertility	Indicators		Samples	Methodology
Chemical	pH		Moist soil	ISO 10390:2005
	CEC		Dry and ground soil (<150 µm)	ISO 23470:2007
	Total trace element		Dry and ground soil (<150 µm)	ISO 11446
	Plant-available trace element		Dry and ground soil (<150 µm)	ISO 14870
	Phosphorus		Dry and ground soil (<150 µm)	ISO 11263
	Ctot / Corg		Dry and ground soil (<150 µm)	ISO 10694
	Ntot		Dry and ground soil (<150 µm)	ISO 13878
	Nitrate / Ammonium		Moist soil	KH_2PO_4 extraction (Internal process)
	HWC / HWN		Moist soil	Hot Water extraction (Internal process)
Physical	Structural stability		Dry soil	ISO 10930:2012 (Rapid wetting by immersion in water)
Biological	Enzymatic Activities	β-Xylosidase	Moist soil	Colorimetric assay (Internal process)
		β-Glucosidase	Moist soil	
		Arylsulfatase	Moist soil	
		Leucine-aminopeptidase	Moist soil	
