# Supplementary material for: Effects of biochar from algae (Sargassum spp.) on the fertility of two chlordecone contaminated West Indies soil
Source: PLoS One. 2025 Dec 30;20(12):e0338385. doi: 10.1371/journal.pone.0338385 (PMC12753066; doi:10.1371/journal.pone.0338385)
Supplement: S3 Table — (PDF) [file pone.0338385.s003.pdf]

SUPPLEMENTARY DATAS

S3 Table. Substrates, reaction buffers and incubation times of the enzymes tested in colorimetric method

Enzymes	Substrat	Substrat concentration	Buffer solution	Incubation time (h)
**β-glucosidase**	pNP-β-D-glucopyranoside	25mM	Sodium acetate, 0.5M (pH 5.8)	1
**β-xylosidase**	pNP-β-D-xylopyranoside	25mM	Sodium acetate, 0.5M (pH 5.8)	3
**Arylsulfatase**	pNP-sulfate	25mM	Sodium acetate, 0.5M (pH 5.8)	1
**Leucine-aminopeptidase**	Leucine-p-nitroaniline	2mM	Tris-HCL 0.1M (pH 8)	3
