# Supplementary material for: Effects of biochar from algae (Sargassum spp.) on the fertility of two chlordecone contaminated West Indies soil
Source: PLoS One. 2025 Dec 30;20(12):e0338385. doi: 10.1371/journal.pone.0338385 (PMC12753066; doi:10.1371/journal.pone.0338385)
Supplement: S4 Table — UA: unamended soil. BCS: Biochar of Sargassum spp. ACD: Activated Carbon DARCO®. The values correspond to the mean ± SE (n = 4). Mean values with different superscript letters for the same stage (a, b, c) are statistically different (P < 0.05) between modalities (ANOVA test). (PDF) [file pone.0338385.s004.pdf]

S4 Table. Mean values (n=4) of Andosol plant-available trace elements and CEC elements as a function of time.

Days	Modality	Plant-Available trace elements (mg.kg^{-1} dry soil)							CEC elements (cmol^{+}.kg^{-1} dry soil)				P Olsen (mg.kg^{-1} dry soil)
		As	Cd	Pb	Ni	Zn	Cu	Fe	Ca	K	Na	Mg	P
0	UA	0.08	0.136	0.39	0.07	6.36	7.90	107	25.6	0.94	0.05	1.18	35.6
		± 0.01	± 0.004	± 0.00^{a}	± 0.00^{a}	± 0.20^{ab}	± 0.16^{a}	± 2.95	± 0.59	± 0.03	± 0.00^{a}	± 0.04^{a}	± 0.89
	BCS	0.07	0.130	0.44	0.10	6.59	7.59	104	25.7	1.00	0.19	1.65	34.6
		± 0.00	± 0.003	± 0.01^{b}	± 0.00^{b}	± 0.21 ^{a}	± 0.18^{ab}	± 3.55	± 0.93	± 0.05	± 0.01^{b}	± 0.09^{b}	± 0.41
	ACD	0.06	0.126	0.37	0.07	5.81	7.25	102	26.5	0.97	0.05	1.16	36.3
		± 0.00	± 0.002	± 0.00^{a}	± 0.00^{a}	± 0.16 ^{b}	± 0.11^{b}	± 1.68	± 0.21	± 0.03	± 0.00^{a}	± 0.04^{a}	± 0.78
7	UA	0.07	0.134	0.39	0.07	5.80	7.57	89.1	25.2	0.93	0.05	0.93	35.1
		± 0.01	± 0.004	± 0.01	± 0.00	± 0.21	± 0.05	± 1.53	± 0.34	± 0.01	± 0.00^{a}	± 0.02^{a}	± 1.93
	BCS	0.06	0.126	0.39	0.09	6.06	7.17	91	24.9	0.99	0.19	1.69	34.6
		± 0.01	± 0.005	± 0.00	± 0.01	± 0.44	± 0.20	± 2.12	± 1.09	± 0.02	± 0.01^{b}	± 0.03^{b}	± 0.41
	ACD	0.06	0.132	0.54	0 .07	5.55	7.22	86.2	25.4	0.97	0.06	1.22	34.3
		± 0.01	± 0.002	± 0.13	± 0.00	± 0.07	± 0.06	± 3.52	± 0.55	± 0.03	± 0.00^{a}	± 0.05^{a}	± 1.93
14	UA	0.08	0.145	0.43	0.07	6.20	7.85	116	27.6	1.03	0.06	1.03	39.0
		± 0.01	± 0.003^{a}	± 0.02	± 0.00^{a}	± 0.12 ^{a}	± 0.18^{a}	± 4.13 ^{a}	± 0.45	± 0.03	± 0 .00^{a}	± 0.04^{a}	± 1.03
	BCS	0.08	0.141	0.41	0.10	6.60	7.12	117	24.9	1.01	0.20	1.77	37.6
		± 0.00	± 0.003^{ab}	± 0.02	± 0.00^{b}	± 0.14 ^{a}	± 0.15^{b}	± 1.82 ^{a}	± 0.72	± 0.02	± 0.01^{b}	± 0.03^{b}	± 0.84
	ACD	0.07	0.129	0.35	0.06	5.34	6.23	98.8	28.5	1.19	0.07	1.43	39.3

		± 0.00	± 0.004^{b}	± 0.03	± 0.00^{c}	± 0.12^{b}	± 0.15^{c}	± 1.23^{b}	± 1.73	± 0.08	± 0.00^{a}	± 0.05^{a}	± 0.39
28	UA	0.07	0.137	0.37	0.07	5.66	7.21	99.3	30.4	1.26	0.08	1.47	36.5
		± 0.00	± 0.005	± 0.02^{ab}	± 0.01^{a}	± 0.23	± 0.22	± 3.74	± 0.45	± 0.01	± 0.00^{a}	± 0.03^{a}	± 0.45
	BCS	0.07	0.141	0.40	0.09	6.10	7.15	100	29.5	1.35	0.29	2.18	35.7
		± 0.00	± 0.004	± 0.03^{a}	± 0.00^{b}	± 0.32	± 0.28	± 1.16	± 0.56	± 0.04	± 0.02^{b}	± 0.05^{b}	± 0.62
	ACD	0.07	0.136	0.31	0.06	5.25	6.55	100	29.7	1.26	0.09	1.42	36.5
		± 0.00	± 0.002	± 0.01^{b}	± 0.00^{a}	± 0.15	± 0.15	± 2.93	± 1.12	± 0.05	± 0.01^{a}	± 0.06^{a}	± 0.54
63	UA	0.07	0.135	0.34	0.06	5.57	6.42	96	28.9	1.26	0.08	1.48	38.9
		± 0.00	± 0.005	± 0.02^{ab}	± 0.00^{ab}	± 0.23	± 0.39	± 4.85	± 0.19	± 0.02	± 0.01^{a}	± 0.03^{a}	± 1.29
	BCS	0.07	0.132	0.37	0.08	5.95	5.99	95.8	30.6	1.43	0.31	2.49	37.3
		± 0.01	± 0.004	± 0.02^{a}	± 0.01^{a}	± 0.22	± 0.10	± 1.93	± 1.36	± 0.06	± 0.02^{b}	± 0.11^{b}	± 0.94
	ACD	0.07	0.169	0.28	0.05	5.01	5.72	95.1	29.7	1.33	0.09	1.55	40.3
		± 0.01	± 0.041	± 0.01^{b}	± 0.00^{b}	± 0.27	± 0.32	± 4.45	± 0.91	± 0.04	± 0.01^{a}	± 0.05^{a}	± 0.58
98	UA	0.09	0.169	0.63	0.09	7.07	9.31	108	27.1	1.00	0.05	1.26	36.3
		± 0.01^{a}	± 0.004^{a}	± 0.09^{a}	± 0.00^{a}	± 0.11^{a}	± 0.09^{a}	± 0.78^{a}	± 1.08	± 0.02	± 0 .00^{a}	± 0.03^{a}	± 1.29^{ab}
	BCS	0.07	0.125	0.42	0.10	5.86	7.47	88.5	27	1.04	0.19	1.63	35.2
		± 0.00^{ab}	± 0.003^{b}	± 0.02^{b}	± 0.00^{a}	± 0.13^{b}	± 0.11^{b}	± 2.81^{b}	± 0.91	± 0.04	± 0.01^{b}	± 0.05^{b}	± 1.61^{a}
	ACD	0.06	0.119	0.35	0.07	5.18	6.96	81.3	24.2	0.93	0.05	1.11	40.2
		± 0.00^{b}	± 0.003^{b}	± 0.02^{b}	± 0.00^{b}	± 0.20^{c}	± 0.19^{b}	± 2.09^{b}	± 0.83	± 0.04	± 0.00^{a}	± 0.05^{a}	± 1.07^{b}
147	UA	0.08	0.138	0.44	0.14	6.07	8.46	100	25.4	0.95	0.05	1.19	36.8
		± 0.00^{a}	± 0.002	± 0.01	± 0.00^{a}	± 0.09	± 0.14^{a}	± 1.01	± 0.26	± 0.03^{a}	± 0.00^{a}	± 0.03^{a}	± 0.55

	BCS	0.08	0.130	0.44	0.13	6.62	7.85	94.9	27.3	1.08	0.19	1.73	36.2
		± 0.00^{a}	± 0.004	± 0.01	± 0.00^{b}	± 0.12	± 0.17^{ab}	± 3.07	± 0.73	± 0.02^{b}	± 0.00^{b}	± 0.03^{b}	± 1.96
	ACD	0.06	0.128	0.37	0.13	5.99	7.51	91.2	25.3	1.07	0.05	1.21	38.2
		± 0.01^{b}	± 0.004	± 0.03	± 0.00^{b}	± 0.35	± 0.31^{b}	± 2.32	± 0.79	± 0.02^{ab}	± 0.00^{a}	± 0.02^{a}	± 0.93
360	UA	0.09	0.132	0.42	0.06	7.59	6.81	99	29.3	1.07	0.02	1.35	52.5
		± 0.01	± 0.006	± 0.01	± 0.00^{a}	± 0.49	±0.31^{a}	± 2.44	± 1.10	± 0.03^{a}	± 0.00^{a}	± 0.03^{a}	± 0.54^{a}
	BCS	0.09	0.119	0.40	0.09	7.50	5.50	97.1	29.4	1.17	0.16	2.42	47.0
		± 0.00	± 0.003	± 0.02	± 0.00^{b}	± 0.36	± 0.13^{b}	± 3.38	± 0.81	± 0.00^{b}	± 0.01^{b}	± 0.04^{b}	± 0.80^{b}
	ACD	0.09	0.121	0.37	0.06	6.18	5.41	97	28.6	1.13	0.02	1.42	54.5
		± 0.00	± 0.004	± 0.02	± 0.00^{a}	± 0.24	± 0.21^{b}	± 2.16	± 0.47	± 0.01^{ab}	± 0.00^{a}	± 0.02^{a}	± 0.86^{a}

UA: unamended soil. BCS: Biochar of Sargasso. ACD: Activated Carbon DARCO®. Values correspond to the mean ± SE (n = 4). Mean values with different superscript letters for the same stage (a, b, c) are statistically different (P < 0.05) between modalities (according to the ANOVA test).
