# Supplementary material for: Effects of biochar from algae (Sargassum spp.) on the fertility of two chlordecone contaminated West Indies soil
Source: PLoS One. 2025 Dec 30;20(12):e0338385. doi: 10.1371/journal.pone.0338385 (PMC12753066; doi:10.1371/journal.pone.0338385)
Supplement: S5 Table — UA: unamended soil. BCS: Biochar of Sargassum spp. ACD: Activated Carbon DARCO®. The values correspond to the mean ± SE (n = 4). Mean values with different superscript letters for the same day (a, b, c) are statistically different (P < 0.05) between modalities (ANOVA test). (PDF) [file pone.0338385.s005.pdf]

S5 Table. Mean values (n=4) of Nitisol plant-available trace elements and CEC elements as a function of time.

Days	Modality	Plant-Available trace elements (mg.kg^{-1} dry soil)							CEC elements (cmol^{+}.kg^{-1} dry soil)				P Olsen (mg.kg^{-1} dry soil)
		As	Cd	Pb	Ni	Zn	Cu	Fe	Ca	K	Na	Mg	P
0	UA	0.22	0.265	2.66	0.20	32.2	6.83	161	19.3	1.71	0.18	4.38	
		±0.01^{a}	±0.008^{a}	±0.08^{a}	±0.01^{a}	±2.16	±0.29^{a}	±5.09	±0.41^{ab}	±0.04^{ab}	±0.01^{a}	±0.07^{a}	
	BCS	0.21	0.220	2.65	0.26	34.9	6.11	158	19.9	1.82	0.42	5.18	
		±0.01^{a}	±0.007^{b}	±0.06^{a}	±0.01^{b}	±1.44	±0.16^{a}	±3.73	±0.23^{a}	±0.04^{a}	±0.02^{b}	±0.11^{b}	
	ACD	0.17	0.226	2.39	0.18	29.3	4.99	143	18.2	1.60	0.20	4.14	
		±0.01^{b}	±0.007^{b}	±0.05^{b}	±0.00^{a}	±0.39	±0.17^{b}	±4.43	±0.55^{b}	±0.06^{b}	±0.01^{a}	±0.13^{a}	
7	UA	0.21	0.246	2.73	0.21	31.8	6.56	171	19.1	1.72	0.16	4.22	
		±0.01	±0.009	±0.19	±0.02^{ab}	±3.27	±0.22^{a}	±6.08	±0.69	±0.05	±0.01^{a}	±0.13^{a}	
	BCS	0.20	0.217	2.65	0.25	33.2	6.06	167	18.8	1.72	0.33	4 .67	
		±0.01	±0.007	±0.09	±0.01^{a}	±2.04	±0.16^{a}	±4.41	±0.38	±0.04	±0.01^{b}	±0.08^{b}	
	ACD	0.18	0.215	2.53	0.17	25.5	5.01	159	19.1	1.60	0.16	3.88	
		±0.01	±0.012	±0.11	±0.01^{b}	±1.24	±0.30^{b}	±7.11	±1.66	±0.03	±0.01^{a}	±0.07^{a}	
14	UA	0.10	0.251	2.68	0.19	28.0	6.86	167	19.6	1.75	0.18	4.40	
		±0.00^{a}	±0.003^{a}	±0.05^{a}	±0.00^{a}	±0.86	±0.14^{a}	±1.98^{a}	±0.60	±0.04	±0.00^{a}	±0.10^{a}	
	BCS	0.18	0.201	2.43	0.24	28.1	5.61	156	19.6	1.76	0.35	4.91	
		±0.00^{b}	±0.003b	±0.07^{ab}	±0.01^{b}	±1.82	±0.18^{b}	±3.06^{ab}	±0.18	±0.02	±0.02^{b}	±0.11^{b}	
	ACD	0.18	0.214	2.25	0.17	25.3	5.25	147	19.6	1.75	0.21	4.36	

		±0.01[b]	±0.007[b]	±0.12[b]	±0.01[a]	±1.20	±0.42[b]	±6.99[b]	±0.10	±0.01	±0.01[a]	±0.04[a]
28	UA	0.21	0.254	2.59	0.19	29.6	6.69	159	19.6	1.69	0.19	4.43
		±0.01[a]	±0.001[a]	±0.05	±0.00[ab]	±0.37	±0.06[a]	±2.20	±0.04	±0.01	±0.00[a]	±0.03[a]
	BCS	0.19	0.195	2.36	0.21	29.4	5.50	149	19.8	1.75	0.34	4.99
		±0.01[ab]	±0.004[b]	±0.04	±0.01[a]	±1.78	±0.26[b]	±3.75	±0.28	±0.06	±0.02[b]	±0.15[b]
	ACD	0.17	0.215	2.34	0.17	26.2	5.10	151	19.5	1.69	0.22	4.37
		±0.01[b]	±0.011[b]	±0.14	±0.01[b]	±1.69	±0.30[b]	±6.67	±0.30	±0.03	±0.01[a]	±0.09[a]
63	UA	0.13	0.227	1.28	0.20	28.1	7.08	202	20.2	1.77	0.21	4.71
		±0.04	±0.013	±0.74	±0.01[a]	±0.86	±0.26[a]	±26.05	±0.43[a]	±0.03[a]	±0.01[a]	±0.07[a]
	BCS	0.19	0.223	2.41	0.25	35 .0	6.28	167	20.5	1.85	0.39	5.39
		±0.01	±0.010	±0.30	±0.02[b]	±2.37	±0.25[a]	±7.20	±0.42[a]	±0 .04[a]	±0.03[b]	±0.16[b]
	ACD	0.17	0.235	2.24	0.17	31.5	4.52	140	18.4	1.63	0.22	4.33
		±0.00	±0.010	±0.05	±0.00[a]	±2.09	±0.12[b]	±2.48	±0.27[b]	±0.02[b]	±0.01[a]	±0.07[a]
98	UA	0.22	0.256	2.14	0.20	20.6	8.12	149	22.2	2.14	0.38	5 .45
		±0.04	±0.038	±0.55	±0.04	±4.88	±0.15[a]	±18.03	±0.41	±0.05	±0.01[b]	±0.12[a]
	BCS	0.23	0.243	2.60	0.26	±25.7	6.73	162	22.9	2.25	0.59	6.31
		±0.01	±0.012	±0.10	±0.01	±1.61	±0.23[ab]	±3.07	±0.32	±0.05	±0.02[a]	±0.15[b]
	ACD	0.18	0.239	2.35	0.19	21.2	5.84	144	20.6	2.05	0.40	5.12
		±0.01	±0.010	±0.10	±0.01	±0.67	±0.17[b]	±4.98	±1.22	±0.12	±0.02[a]	±0.32[a]
147	UA	0.11	0.333	1.38	0.20	23.5	8.03	224	24.7	2.45	0.21	6.28
		±0.07	±0.028	±0.79	±0.01	±2.43	±0.20	±18.03	±0.92[a]	±0.05[a]	±0.03[a]	±0.20[a]

	BCS	0.20	0.328	1.58	0.28	26.5	7.04	162	22.3	2.18	0.62	6.39
		±0.05	±0.032	±0.66	±0.01	±3.27	±0.88	±3.07	±0.19 ^{ab}	±0.03 ^{b}	±0.01 ^{b}	±0.06 ^{a}
	ACD	0.24	0.292	2.88	0.24	28.9	8.34	144	21.6	2.12	0.43	5.42
		±0.01	±0.008	±0.15	±0.01	±1.64	±1.51	±4.98	±0.61 ^{b}	±0.07 ^{b}	±0.01 ^{a}	±0.20 ^{b}
360	UA	0.16	0.303	2.25	0.24	32.5	8.56	224	20.0	1.76	0.21	4.72
		±0.04	±0.018^{a}	±0.96	±0.05	±6.61	±1.19	±23.84	±0.36 ^{ab}	±0.03 ^{ab}	±0.01 ^{a}	±0.06 ^{a}
	BCS	0.21	0.223^{b}	2.56	0.25	±30.9	5.74	230	20.8	1.86	0.43	5.97
		±0.02	±0.011	±0.18	±0.01	±2.41	±0.56	±39.81	±0.33 ^{a}	±0.02 ^{a}	±0.01 ^{b}	±0.14 ^{b}
	ACD	0.20	0.249	2.80	0.22	31.1	5.92	178	19.1	1.70	0.24	4.52
		±0.01	±0.017^{ab}	±0.29	±0.01	±4.85	±0.73	±8.31	±0.37 ^{b}	±0.03 ^{b}	±0.01 ^{a}	±0.06 ^{a}

UA: unamended soil. BCS: Biochar of Sargasso. ACD: Activated Carbon DARCO®. Values correspond to the mean ± SE (n = 4). Mean values with different superscript letters for the same day (a, b, c) are statistically different ($P < 0.05$) between modalities (according to the ANOVA test).
