# Supplementary material for: Effects of biochar from algae (Sargassum spp.) on the fertility of two chlordecone contaminated West Indies soil
Source: PLoS One. 2025 Dec 30;20(12):e0338385. doi: 10.1371/journal.pone.0338385 (PMC12753066; doi:10.1371/journal.pone.0338385)
Supplement: S6 Table — Sol: soil type (Nitisol or Andosol), moda: modality (BCS, UA, ACD), rep: duplication, TC = HWC, TN = HWN, DE = chlordecone environmental availability, SS = soil structural stability. (PDF) [file pone.0338385.s006.pdf]

S6 Table: Data from the study.

	A	B	C	D	E	F	G	H	I	J	K	L	M	N	O	P	Q	R	S
1	**ID**	**sol**	**moda**	**rep**	**tps**	**pH**	**TC**	**TN**	**NH4+**	**NO3-**	**B-glu**	**ARS**	**Xylo**	**Leu**	**DE**	**SS**	**Stot**	**Fetot**	**Pbtot**
2	**E2.A.0.T.1**	Andosol	[illegible]	1	T0	7,29	545,03	169,51	0,00	1,61	50,400085	335,70042	107,83172	133,83562	1,61	2,75	1095,47	95721,31	16,21
3	**E2.A.0.T.2**	Andosol	[illegible]	2	T0	7,34	554,82	187,88	0,00	1,61	126,26907	338,47434	115,69117	144,52055	1,39	2,90	861,65	82389,12	16,93
4	**E2.A.0.T.3**	Andosol	UA	3	T0	7,38	578,36	185,46	0,00	1,58	122,10818	354,56311	124,66019	132,73973	1,39	2,97	1002,20	83830,76	15,40
5	**E2.A.0.T.4**	Andosol	UA	4	T0	7,35	640,51	175,89	0,00	1,63	141,80305	380,638	120,31438	137,39726	1,50	2,98	875,88	87181,07	16,45
6	**E2.A.0.S.1**	Andosol	BCS	1	T0	7,23	464,178	189,31	0,00	1,43	112,07337	512,39476	156,8182	11,384319	0,82	2,90	1442,50	80802,39	15,68
7	**E2.A.0.S.2**	Andosol	BCS	2	T0	7,25	431,73	198,33	0,00	1,47	116,77675	474,52168	126,00645	2,040214	0,64	2,82	1211,59	80824,94	17,66
8	**E2.A.0.S.3**	Andosol	BCS	3	T0	7,27	440,31	189,97	0,00	1,48	125,64958	522,24336	144,73719	0	0,70	2,85	1477,53	78606,83	17,22
9	**E2.A.0.S.4**	Andosol	BCS	4	T0	7,28	446,69	181,94	0,00	1,43	157,57588	621,41136	205,97065	0	0,30	3,00	1340,79	89242,29	16,65
10	**E2.A.0.D.1**	Andosol	ACD	1	T0	7,30	258,15	205,37	2,25	1,28	190,77155	489,86352	192,16769	0	0,88	3,12	854,20	75720,29	15,52
11	**E2.A.0.D.2**	Andosol	ACD	2	T0	7,37	266,40	203,83	5,04	1,36	221,89508	524,46391	243,65438	0	0,37	2,81	823,59	82571,07	17,67
12	**E2.A.0.D.3**	Andosol	ACD	3	T0	7,41	285,98	217,69	3,25	1,39	192,49111	513,48815	360,6073	0	0,25	2,95	880,42	75397,32	17,90
13	**E2.A.0.D.4**	Andosol	ACD	4	T0	7,25	263,98	212,85	0,00	1,39	213,49035	553,01238	298,98868	0	0,43	2,71	960,42	88614,07	19,41
14	**E2.A.7.T.1**	Andosol	UA	1	T7	7,34	635,50	172,21	0,00	1,65	49,759949	388,40499	106,90707	131,91781					
15	**E2.A.7.T.2**	Andosol	UA	2	T7	7,46	599,86	175,84	2,35	1,72	120,44383	399,77809	111,62275	137,94521					
16	**E2.A.7.T.3**	Andosol	UA	3	T7	7,45	672,02	167,70	**NA**	**NA**	117,39251	373,4258	109,95839	141,78082					
17	**E2.A.7.T.4**	Andosol	UA	4	T7	7,45	648,70	174,63	0,00	1,55	125,99168	395,06241	115,8761	131,64384					
18	**E2.A.7.S.1**	Andosol	BCS	1	T7	7,29	485,46	179,03	**NA**	1,48	114,42257	950,50387	107,56077	4,845518					
19	**E2.A.7.S.2**	Andosol	BCS	2	T7	7,34	531,99	174,96	**NA**	**NA**	133,39688	632,09904	108,57441	6,2860839					
20	**E2.A.7.S.3**	Andosol	BCS	3	T7	7,39	461,48	183,21	0,00	1,40	141,80053	737,00802	174,80517	14,536655					
21	**E2.A.7.S.4**	Andosol	BCS	4	T7	7,42	512,96	185,19	0,00	1,46	151,4823	587,24557	147,89159	0					
22	**E2.A.7.D.1**	Andosol	ACD	1	T7	7,47	290,32	199,60	**NA**	**NA**	201,05063	548,10685	210,33613	0					
23	**E2.A.7.D.2**	Andosol	ACD	2	T7	7,51	239,28	210,27	0,00	1,40	186,74926	524,04401	223,30927	0					
24	**E2.A.7.D.3**	Andosol	ACD	3	T7	7,56	295,16	212,91	0,00	1,39	176,34161	540,289	272,02086	0					
25	**E2.A.7.D.4**	Andosol	ACD	4	T7	7,53	275,25	210,82	0,00	1,31	194,20811	516,03183	274,53381	0					
26	**E2.A.14.T.1**	Andosol	UA	1	T14	7,33	623,50	190,67	2,53	1,58	120,44383	295,75589	118,09524	170,31963	1,46				
27	**E2.A.14.T.2**	Andosol	UA	2	T14	7,45	604,91	197,38	1,99	1,58	154,8405	377,0319	116,52335	176,1035	1,42				
28	**E2.A.14.T.3**	Andosol	UA	3	T14	7,39	601,94	201,01	3,71	1,64	150,957	327,65603	121,14656	163,92694	1,51				
29	**E2.A.14.T.4**	Andosol	UA	4	T14	7,36	661,56	205,19	0,00	1,62	160,38835	338,47434	117,81785	174,27702	1,36				
30	**E2.A.14.S.1**	Andosol	BCS	1	T14	7,26	464,66	196,94	0,00	1,53	198,00061	548,03195	119,55856	16,50941	0,59				
31	**E2.A.14.S.2**	Andosol	BCS	2	T14	7,36	456,41	201,67	0,00	1,53	203,712	519,77427	87,471509	6,0207178	0,80				
32	**E2.A.14.S.3**	Andosol	BCS	3	T14	7,27	418,46	202,22	0,00	1,40	199,26737	618,75247	123,69431	20,534179	0,89				
33	**E2.A.14.S.4**	Andosol	BCS	4	T14	7,41	445,19	210,25	0,00	1,50	241,20923	809,16497	41,719372	9,9395533	0,73				
34	**E2.A.14.D.1**	Andosol	ACD	1	T14	7,40	243,12	209,48	2,59	1,43	241,81653	586,27875	206,965	0	0,31				
35	**E2.A.14.D.2**	Andosol	ACD	2	T14	7,43	256,32	216,63	3,32	1,47	280,63214	599,96842	126,72184	0	0,24				
36	**E2.A.14.D.3**	Andosol	ACD	3	T14	7,36	241,80	240,28	2,02	1,56	271,05388	603,45703	171,29365	0	0,45				
37	**E2.A.14.D.4**	Andosol	ACD	4	T14	7,41	249,72	225,43	0,00	1,38	283,80663	665,21834	177,46931	0	0,60				

	A	B	C	D	E	F	G	H	I	J	K	L	M	N	O	P	Q	R	S
1	**ID**	**sol**	**moda**	**rep**	**tps**	**pH**	**TC**	**TN**	**NH4+**	**NO3-**	**B-glu**	**ARS**	**Xylo**	**Leu**	**DE**	**SS**	**Stot**	**Fetot**	**Pbtot**
38	**E2.A.28.T.1**	Andosol	UA	1	T28	7,38	673,74	207,50	0,71	1,69	154,8405	418,64078	130,5779	159,05632		2,87			
39	**E2.A.28.T.2**	Andosol	UA	2	T28	7,40	713,78	210,58	1,78	1,68	137,64216	387,57282	123,92048	166,05784		3,15			
40	**E2.A.28.T.3**	Andosol	UA	3	T28	7,37	720,05	226,86	NA	1,69	130,70735	372,03883	120,22191	158,14307		2,94			
41	**E2.A.28.T.4**	Andosol	UA	4	T28	7,49	704,10	217,84	0,00	1,32	124,88211	371,20666	117,63292	163,92694		2,80			
42	**E2.A.28.S.1**	Andosol	BCS	1	T28	7,35	479,04	216,41	2,87	1,35	150,26752	667,42009	130,75508	24,734307		3,00			
43	**E2.A.28.S.2**	Andosol	BCS	2	T28	7,35	483,88	216,74	0,00	1,54	143,60953	667,42009	165,28972	20,161213		3,01			
44	**E2.A.28.S.3**	Andosol	BCS	3	T28	7,33	494,11	224,00	0,00	1,57	176,67688	686,8193	139,48812	16,868342		2,96			
45	**E2.A.28.S.4**	Andosol	BCS	4	T28	7,42	496,75	220,81	0,00	1,30	187,91649	784,04474	249,78332	18,31782		3,12			
46	**E2.A.28.D.1**	Andosol	ACD	1	T28	7,47	217,57	232,58	0,00	1,49	224,68294	621,84865	271,39641	0		3,02			
47	**E2.A.28.D.2**	Andosol	ACD	2	T28	7,45	250,68	241,71	0,00	1,48	217,98057	618,04653	328,14948	0		2,92			
48	**E2.A.28.D.3**	Andosol	ACD	3	T28	7,42	296,55	256,34	0,00	1,58	232,45277	618,42744	325,09372	0		2,99			
49	**E2.A.28.D.4**	Andosol	ACD	4	T28	7,44	252,22	252,60	0,00	1,27	238,52052	614,61145	371,96104	0		3,08			
50	**E2.A.35.T.1**	Andosol	UA	1	T35		617,14	207,03			141,52566	414,2025	120,86916	152,66362	1,56				
51	**E2.A.35.T.2**	Andosol	UA	2	T35		656,08	210,88			150,67961	423,91123	123,73555	168,49315	1,55				
52	**E2.A.35.T.3**	Andosol	UA	3	T35		691,61	223,86			151,78918	436,3939	126,78687	161,79604	1,67				
53	**E2.A.35.T.4**	Andosol	UA	4	T35		651,68	213,85			158,16921	456,08877	115,96856	155,70776	1,64				
54	**E2.A.35.S.1**	Andosol	BCS	1	T35		477,44	216,49			166,16239	626,74475	107,56077	17,22864	0,87				
55	**E2.A.35.S.2**	Andosol	BCS	2	T35		460,94	224,63			152,09018	618,75247	101,00977	12,306132	0,73				
56	**E2.A.35.S.3**	Andosol	BCS	3	T35		441,80	173,70			163,08831	629,41927	117,72144	NA	0,81				
57	**E2.A.35.S.4**	Andosol	BCS	4	T35		437,84	227,60			153,91579	722,62153	113,06493	13,339305	0,84				
58	**E2.A.35.D.1**	Andosol	ACD	1	T35		228,51	229,14			215,73767	602,29558	206,01095	0	0,24				
59	**E2.A.35.D.2**	Andosol	ACD	2	T35		247,76	235,85			234,66299	608,85835	210,82182	0	0,29				
60	**E2.A.35.D.3**	Andosol	ACD	3	T35		277,68	249,38			222,4532	598,02476	227,45071	0	0,19				
61	**E2.A.35.D.4**	Andosol	ACD	4	T35		262,61	243,88			237,97025	604,23053	303,17252	0	0,23				
62	**E2.A.63.T.1**	Andosol	UA	1	T63	7,24	494,23	216,03	0,00	1,70	149,57004	434,17476	126,78687	139,57382	2,13	2,75			
63	**E2.A.63.T.2**	Andosol	UA	2	T63	7,27	521,07	249,69	0,00	1,43	135,42302	421,13731	122,81091	152,66362	1,99	2,84			
64	**E2.A.63.T.3**	Andosol	UA	3	T63	7,21	612,70	238,69	10,90	1,47	168,15534	450,81831	131,8724	150,53272	2,15	2,82			
65	**E2.A.63.T.4**	Andosol	UA	4	T63	7,21	627,66	233,41	0,00	1,74	144,57698	439,72261	131,50254	142,61796	1,90	2,83			
66	**E2.A.63.S.1**	Andosol	BCS	1	T63	7,13	385,33	213,17	0,00	1,32	208,81263	891,49701	167,8745	27,103345	1,13	2,76			
67	**E2.A.63.S.2**	Andosol	BCS	2	T63	7,12	399,74	225,60	0,00	1,34	177,29841	893,64179	211,32351	19,789687	1,13	2,97			
68	**E2.A.63.S.3**	Andosol	BCS	3	T63	7,09	440,00	240,67	10,25	1,34	216,50595	917,43851	196,98664	26,30736	1,11	2,98			
69	**E2.A.63.S.4**	Andosol	BCS	4	T63	7,14	427,68	247,93	9,95	1,26	199,90127	892,56902	171,03483	28,30936	0,93	2,95			
70	**E2.A.63.D.1**	Andosol	ACD	1	T63	7,25	219,56	243,75	0,00	1,31	225,23969	629,0305	332,01548	0	0,28	2,94			
71	**E2.A.63.D.2**	Andosol	ACD	2	T63	7,27	254,21	252,44	0,00	1,30	282,22052	639,51473	373,7456	0	0,23	2,97			
72	**E2.A.63.D.3**	Andosol	ACD	3	T63	7,25	272,47	253,43	0,00	1,27	257,61275	669,55353	329,68966	0	**NA**	2,78			
73	**E2.A.63.D.4**	Andosol	ACD	4	T63	7,22	292,82	255,52	0,00	1,26	280,10218	710,7722	477,11233	0	0,36	2,80			

	A	B	C	D	E	F	G	H	I	J	K	L	M	N	O	P	Q	R	S
1	**ID**	**sol**	**moda**	**rep**	**tps**	**pH**	**TC**	**TN**	**NH4+**	**NO3-**	**B-glu**	**ARS**	**Xylo**	**Leu**	**DE**	**SS**	**Stot**	**Fetot**	**Pbtot**
74	**E2.A.98.T.1**	Andosol	UA	1	T98	7,38	479,35	199,68	0,00	1,51	156,78225	421,4147	123,55062	131,96347	1,87				
75	**E2.A.98.T.2**	Andosol	UA	2	T98	7,39	521,37	195,28	0,00	1,57	140,41609	390,06935	118,37263	NA	2,16				
76	**E2.A.98.T.3**	Andosol	UA	3	T98	7,47	489,58	202,98	0,00	1,50	145,96394	414,2025	140,47157	131,05023	2,07				
77	**E2.A.98.T.4**	Andosol	UA	4	T98	7,44	514,55	196,49	0,00	1,24	129,32039	377,58669	113,84189	139,26941	1,89				
78	**E2.A.98.S.1**	Andosol	BCS	1	T98	7,31	381,34	196,60	11,59	1,58	136,39096	591,57787	97,553024	17,590314	0,98				
79	**E2.A.98.S.2**	Andosol	BCS	2	T98	7,42	396,96	195,06	12,31	1,38	144,81715	535,49069	101,00977	7,3081309	1,01				
80	**E2.A.98.S.3**	Andosol	BCS	3	T98	7,34	392,23	204,74	0,00	1,40	166,16239	605,53542	112,71373	24,734307	**NA**				
81	**E2.A.98.S.4**	Andosol	BCS	4	T98	7,76	**NA**	**NA**	**NA**	**NA**	NA	466,60453	NA	NA	**NA**				
82	**E2.A.98.D.1**	Andosol	ACD	1	T98	7,43	187,74	203,42	0,00	1,60	225,23969	557,89331	181,68898	0	0,21				
83	**E2.A.98.D.2**	Andosol	ACD	2	T98	7,44	250,77	214,31	0,00	1,06	235,21487	584,70165	NA	0	0,29				
84	**E2.A.98.D.3**	Andosol	ACD	3	T98	7,46	221,07	213,65	0,00	0,96	248,92503	580,74749	239,7751	0	0,22				
85	**E2.A.98.D.4**	Andosol	ACD	4	T98	7,45	213,59	209,14	0,00	1,20	291,70326	NA	362,3268	34,506379	0,37				
86	**E2.A.147.T.1**	Andosol	UA	1	T147	7,07	484,67	239,31	4,77	1,80	69,126214	176,75451	38,206195	127,90462	1,93	2,96	706,41	53342,69	75,70
87	**E2.A.147.T.2**	Andosol	UA	2	T147	7,14	549,35	235,35	**NA**	1,32	78,280166	213,09293	40,98012	147,89447	1,89	2,92	796,23	51093,37	76,84
88	**E2.A.147.T.3**	Andosol	UA	3	T147	7,19	597,09	221,38	2,18	1,42	68,294036	167,04577	37,466482	113,19127	NA	2,93	735,63	48433,31	76,32
89	**E2.A.147.T.4**	Andosol	UA	4	T147	6,93	522,40	227,54	2,08	1,80	84,937587	209,48682	62,061951	109,43683	1,91	2,85	815,43	51671,56	72,81
90	**E2.A.147.S.1**	Andosol	BCS	1	T147	7,13	481,92	247,67	**NA**	1,68	53,804776	233,35159	27,562541	4,4371797	1,16	3,00	1142,38	48210,88	65,63
91	**E2.A.147.S.2**	Andosol	BCS	2	T147	7,03	493,69	269,67	5,46	1,94	62,180877	191,05828	30,530976	16,390069	0,94	3,04	1155,03	41670,31	68,13
92	**E2.A.147.S.3**	Andosol	BCS	3	T147	7,07	423,73	279,35	4,26	1,43	48,810118	236,6192	40,999388	21,53586	0,77	2,93	1147,46	47182,85	70,27
93	**E2.A.147.S.4**	Andosol	BCS	4	T147	7,13	414,05	261,75	3,15	1,90	42,186411	190,42923	42,445142	15,32275	0,91	2,98	1326,59	46178,37	71,49
94	**E2.A.147.D.1**	Andosol	ACD	1	T147	7,2	207,91	295,41	1,97	1,73	69,416166	245,10294	41,567199	7,2428519	0,21	2,96	580,63	48911,06	65,62
95	**E2.A.147.D.2**	Andosol	ACD	2	T147	7,22	274,46	283,75	2,21	1,28	95,816715	279,04149	63,774684	3,9721432	0,17	2,99	683,47	45005,92	66,31
96	**E2.A.147.D.3**	Andosol	ACD	3	T147	7,33	326,05	285,40	2,18	1,40	93,327112	NA	33,696622	13,070669	0,20	2,86	604,43	42583,74	64,50
97	**E2.A.147.D.4**	Andosol	ACD	4	T147	7,28	272,37	290,24	NA	1,74	83,316923	NA	53,193733	10,91556	0,22	2,86	680,19	40340,88	68,87
98	**E2.A.360.T.1**	Andosol	UA	1	T360	7,4	480,21	361,15	0,06	2,30	31,678225	156,78225	49,764216	110,04566	1,49	2,76	707,05	72187,21	7,77
99	**E2.A.360.T.2**	Andosol	UA	2	T360	7,47	525,09	352,46	3,07	2,47	22,801664	168,71012	32,288488	82,343988	1,66	2,70	737,79	64490,10	7,08
100	**E2.A.360.T.3**	Andosol	UA	3	T360	7,64	514,09	402,51	**NA**	2,85	65,242718	172,87101	48,099861	91,476408	1,66	2,61	785,13	78845,83	8,31
101	**E2.A.360.T.4**	Andosol	UA	4	T360	7,59	610,67	177,12	1,24	1,20	55,256588	155,11789	42,829404	86,605784	1,99	2,78	798,97	71201,22	7,50
102	**E2.A.360.S.1**	Andosol	BCS	1	T360	7,34	416,30	327,16	8,02	2,29	16,631108	99,821214	15,860734	6,6621085	0,85	3,15	1323,15	76025,25	7,89
103	**E2.A.360.S.2**	Andosol	BCS	2	T360	7,46	454,14	320,45	4,58	2,15	13,948208	141,80053	10,853913	7,3081309	0,89	2,79	1278,81	73254,73	7,40
104	**E2.A.360.S.3**	Andosol	BCS	3	T360	7,38	441,05	343,55	4,62	2,31	0	141,19818	18,69794	7,3081309	0,82	2,57	1144,70	64352,38	7,45
105	**E2.A.360.S.4**	Andosol	BCS	4	T360	7,35	444,13	350,81	5,23	2,44	0	144,21318	7,09945	14,38411	0,74	2,81	1262,54	64109,47	7,54
106	**E2.A.360.D.1**	Andosol	ACD	1	T360	7,46	313,34	386,01	3,95	2,36	41,779517	261,93152	25,900462	0	0,14	2,82	780,68	73945,46	7,08
107	**E2.A.360.D.2**	Andosol	ACD	2	T360	7,31	300,25	377,43	3,19	2,35	17,491119	240,71892	22,834976	0	0,14	2,71	757,55	61047,69	7,61
108	**E2.A.360.D.3**	Andosol	ACD	3	T360	7,29	335,78	375,78	9,11	2,07	17,491119	269,44946	38,517208	NA	0,15	2,82	816,62	76114,78	7,26
109	**E2.A.360.D.4**	Andosol	ACD	4	T360	7,57	375,49	NA	2,56	1,49	44,376852	242,91306	38,517208	0	0,13	2,71	765,35	69067,41	12,98

	A	T	U	V	W	X	Y	Z	AA	AB	AC	AD	AE	AF	AG	AH	AI
1	**ID**	**Zntot**	**Astot**	**As**	**Cu**	**Fe**	**K**	**Mg**	**Na**	**Ni**	**Pb**	**Zn**	**Phos**	**CEC**	**Corg**	**N**	**C/N**
2	**E2.A.0.T.1**	123,54	1,03	0,10	7,63	105,05	54,12	24,87	43,51	0,07	0,39	6,08	38,21	44,99	49,5	5	9,90
3	**E2.A.0.T.2**	115,20	0,00	0,06	7,65	101,06	52,27	24,79	43,03	0,07	0,39	5,97	34,63	40,16	49,6	5,1	9,73
4	**E2.A.0.T.3**	109,70	0,00	0,07	8,02	108,06	55,89	25,91	45,27	0,07	0,39	6,55	35,28	50,38	50,3	5,1	9,86
5	**E2.A.0.T.4**	118,96	0,96	0,09	8,30	115,03	62,95	29,51	51,89	0,07	0,38	6,83	34,32	45,49	51,1	4,9	10,43
6	**E2.A.0.S.1**	113,57	2,25	0,06	7,58	106,86	68,13	44,37	140,39	0,11	0,46	6,73	34,07	47,53	61,8	5,1	12,12
7	**E2.A.0.S.2**	108,77	1,33	0,07	7,35	97,43	64,05	40,88	127,68	0,10	0,41	6,18	33,85	43,97	56,3	5	11,26
8	**E2.A.0.S.3**	114,76	2,44	0,07	7,34	98,63	64,46	39,11	117,27	0,10	0,45	6,33	34,84	53,53	63,6	5,1	12,47
9	**E2.A.0.S.4**	117,43	2,55	0,08	8,11	112,44	71,16	40,84	122,56	0,11	0,45	7,10	35,64	45,60	57,8	4,9	11,80
10	**E2.A.0.D.1**	115,96	0,00	0,05	7,30	101,12	55,86	25,89	49,88	0,07	0,38	5,92	34,39	44,00	63,9	4,7	13,60
11	**E2.A.0.D.2**	114,04	0,00	0,06	7,32	106,53	61,13	27,39	53,82	0,07	0,37	6,03	35,70	42,80	58,6	4,8	12,21
12	**E2.A.0.D.3**	118,17	0,00	0,06	7,42	100,26	54,65	24,99	48,28	0,07	0,38	5,95	37,46	46,02	61,3	4,9	12,51
13	**E2.A.0.D.4**	114,11	1,36	0,06	6,94	98,83	52,48	24,00	46,63	0,06	0,37	5,34	37,71	31,65	62,1	4,9	12,67
14	**E2.A.7.T.1**			0,06	7,64	84,58	52,00	25,08	48,44	0,06	0,41	5,42	38,05	**NA**	50,4	5,1	9,88
15	**E2.A.7.T.2**			0,06	7,56	89,94	50,99	24,75	47,03	0,07	0,37	6,34	36,49	47,46	49,5	4,9	10,10
16	**E2.A.7.T.3**			0,08	7,65	90,56	56,82	27,21	52,64	0,09	0,41	5,94	29,47	46,20	49,7	4,9	10,14
17	**E2.A.7.T.4**			0,07	7,44	91,31	53,77	25,66	50,93	0,07	0,38	5,51	36,56	43,11	46,6	4,9	9,51
18	**E2.A.7.S.1**			0,08	7,71	96,53	68,83	44,09	140,57	0,11	0,40	7,37	35,27	42,27	62	5,1	12,16
19	**E2.A.7.S.2**			0,06	6,81	91,50	58,79	37,35	119,97	0,08	4,09	5,44	34,98	41,76	59,8	5	11,96
20	**E2.A.7.S.3**			0,06	7,22	89,46	61,30	40,14	127,77	0,09	0,39	5,79	33,43	46,29	58,2	5	11,64
21	**E2.A.7.S.4**			0,05	6,96	86,46	60,19	37,72	122,64	0,08	0,39	5,62	34,71	42,35	61,1	5,3	11,53
22	**E2.A.7.D.1**			0,05	7,10	87,79	58,15	28,04	58,41	0,07	0,39	5,52	33,71	44,23	66,6	4,9	13,59
23	**E2.A.7.D.2**			0,08	7,23	95,50	60,64	28,51	60,74	0,08	0,39	5,76	29,87	41,21	67	4,9	13,67
24	**E2.A.7.D.3**			0,07	7,15	79,76	61,01	29,37	59,06	0,06	0,46	5,43	34,35	46,66	63,6	4,9	12,98
25	**E2.A.7.D.4**			0,06	7,37	81,94	59,29	29,05	57,62	0,07	0,94	5,47	39,26	41,52	60,8	4,9	12,41
26	**E2.A.14.T.1**			0,09	7,78	115,30	57,55	27,86	52,90	0,07	0,44	6,20	38,11	44,02	48,8	4,8	10,17
27	**E2.A.14.T.2**			0,08	7,47	116,54	58,10	27,44	53,84	0,07	0,41	5,98	41,28	47,31	49,7	5	9,94
28	**E2.A.14.T.3**			0,07	7,82	105,11	56,60	26,84	54,74	0,07	0,39	6,11	36,54	47,85	49,9	5,2	9,60
29	**E2.A.14.T.4**			0,10	8,33	125,30	60,98	27,84	58,50	0,08	0,47	6,54	39,90	41,94	47,9	5,1	9,39
30	**E2.A.14.S.1**			0,08	7,25	113,47	65,77	43,37	134,70	0,10	NA	6,51	38,74	47,49	63,6	5,4	11,78
31	**E2.A.14.S.2**			0,08	7,24	117,35	73,89	49,58	156,70	0,10	0,44	6,86	35,14	45,20	64,1	5,2	12,33
32	**E2.A.14.S.3**			0,08	6,66	114,82	70,82	46,19	144,47	0,09	0,39	6,25	38,56	40,55	60,5	5,2	11,63
33	**E2.A.14.S.4**			0,08	7,32	121,73	73,86	47,46	148,28	0,10	0,40	6,76	38,11	43,38	57,8	5	11,56
34	**E2.A.14.D.1**			0,08	6,42	96,64	61,21	28,99	63,34	0,06	0,31	5,38	38,74	44,05	61,7	5	12,34
35	**E2.A.14.D.2**			0,06	6,52	96,66	66,69	31,42	68,32	0,05	0,32	5,55	39,24	44,70	62,4	4,8	13,00
36	**E2.A.14.D.3**			0,07	6,15	100,98	58,98	27,62	59,73	0,06	0,33	5,44	40,44	48,09	65,2	5,1	12,78
37	**E2.A.14.D.4**			0,06	5,84	100,81	53,05	24,69	51,82	0,06	0,44	4,98	38,84	45,40	54,5	4,7	11,60

	A	T	U	V	W	X	Y	Z	AA	AB	AC	AD	AE	AF	AG	AH	AI
1	**ID**	**Zntot**	**Astot**	**As**	**Cu**	**Fe**	**K**	**Mg**	**Na**	**Ni**	**Pb**	**Zn**	**Phos**	**CEC**	**Corg**	**N**	**C/N**
38	**E2.A.28.T.1**			0,07	7,67	103,16	58,00	27,55	61,41	0,08	0,39	5,93	35,57	46,33	47,6	5	9,52
39	**E2.A.28.T.2**			0,06	6,87	88,40	51,52	24,51	56,92	0,05	0,38	5,09	37,13	46,59	48,1	4,8	10,02
40	**E2.A.28.T.3**			0,07	7,51	104,97	62,23	30,03	69,67	0,07	0,32	5,66	37,39	44,58	48,3	4,9	9,86
41	**E2.A.28.T.4**			0,07	6,78	100,68	65,85	46,18	142,16	0,09	0,38	5,98	35,92	47,81	48	4,9	9,80
42	**E2.A.28.S.1**			0,07	6,93	99,13	66,29	45,88	143,74	0,10	0,37	5,93	37,05	45,06	60,7	5	12,14
43	**E2.A.28.S.2**			0,07	6,97	100,81	67,18	44,87	140,10	0,09	0,38	5,99	36,49	46,89	54,4	4,9	11,10
44	**E2.A.28.S.3**			0,08	7,98	103,08	67,25	38,84	166,98	0,10	0,49	6,98	34,64	48,21	59,6	5,2	11,46
45	**E2.A.28.S.4**			0,07	6,71	97,69	63,31	29,81	79,26	0,09	0,38	5,49	34,68	43,98	52,6	4,9	10,73
46	**E2.A.28.D.1**			0,06	6,65	96,61	60,12	29,10	69,67	0,06	0,30	5,15	37,60	36,45	66,5	5,1	13,04
47	**E2.A.28.D.2**			0,07	6,87	104,42	59,56	28,33	71,57	0,06	0,35	5,66	37,28	48,51	55,4	4,8	11,54
48	**E2.A.28.D.3**			0,08	6,52	105,73	59,49	28,62	69,66	0,07	0,29	5,28	35,78	42,51	61,7	5	12,34
49	**E2.A.28.D.4**			0,07	6,16	93,74	56,47	27,05	56,61	0,06	0,31	4,92	35,44	43,32	61,2	5	12,24
50	**E2.A.35.T.1**																
51	**E2.A.35.T.2**																
52	**E2.A.35.T.3**																
53	**E2.A.35.T.4**																
54	**E2.A.35.S.1**																
55	**E2.A.35.S.2**																
56	**E2.A.35.S.3**																
57	**E2.A.35.S.4**																
58	**E2.A.35.D.1**																
59	**E2.A.35.D.2**																
60	**E2.A.35.D.3**																
61	**E2.A.35.D.4**																
62	**E2.A.63.T.1**			0,08	7,36	108,00	60,83	29,24	62,66	0,07	0,37	6,22	41,82	45,77	48,7	5	9,74
63	**E2.A.63.T.2**			0,07	6,54	96,96	57,69	27,37	60,54	0,06	0,34	5,39	35,57	45,55	47,7	5,1	9,35
64	**E2.A.63.T.3**			0,07	6,36	94,94	52,37	25,03	52,25	0,06	0,27	5,13	38,84	47,60	48,7	5,1	9,55
65	**E2.A.63.T.4**			0,08	5,44	84,31	72,36	50,04	NA	0,07	0,37	5,53	39,38	47,75	48,7	5,1	9,55
66	**E2.A.63.S.1**			0,09	6,14	98,63	75,37	55,14	164,98	0,09	0,41	6,22	38,68	46,82	59,2	5,1	11,61
67	**E2.A.63.S.2**			0,08	6,02	96,44	69,68	51,47	155,18	0,09	0,35	6,03	37,46	49,85	60,8	5,3	11,47
68	**E2.A.63.S.3**			0,06	5,69	90,15	64,96	46,82	141,73	0,08	0,36	6,23	34,63	41,59	57,7	5	11,54
69	**E2.A.63.S.4**			0,05	6,11	97,90	59,58	28,62	64,52	0,06	0,35	5,32	38,59	44,71	60,9	5,3	11,49
70	**E2.A.63.D.1**			0,05	5,11	87,85	56,00	27,28	60,60	0,05	0,25	4,54	41,76	44,07	61,1	4,8	12,73
71	**E2.A.63.D.2**			0,08	6,38	105,00	69,18	33,53	77,58	0,06	0,30	5,60	38,95	44,57	64,9	5,1	12,73
72	**E2.A.63.D.3**			0,07	5,66	92,33	56,35	27,33	60,86	0,05	0,29	4,90	40,17	44,64	63	4,9	12,86
73	**E2.A.63.D.4**			NA	NA	NA	NA	**NA**	**NA**	**NA**	**NA**	**NA**	40,49	42,26	57,3	4,8	11,94

	A	T	U	V	W	X	Y	Z	AA	AB	AC	AD	AE	AF	AG	AH	AI
1	ID	Zntot	Astot	As	Cu	Fe	K	Mg	Na	Ni	Pb	Zn	Phos	CEC	Corg	N	C/N
74	E2.A.98.T.1			0,27	7,72	166,09	142,94	162,94	211,46	0,24	2,68	25,54	38,87	40,35	48,3	5,1	9,47
75	E2.A.98.T.2			0,26	7,65	163,54	136,04	155,48	195,84	0,22	2,67	24,82	35,86	42,63	46,3	5	9,26
76	E2.A.98.T.3			NA	NA	NA	NA	NA	NA	NA	NA	NA	36,21	39,79	46,9	5	9,38
77	E2.A.98.T.4			0,25	7,79	172,35	145,36	165,89	207,58	0,26	2,72	26,16	34,39	39,71	50,7	5,3	9,57
78	E2.A.98.S.1			0,26	6,27	162,87	143,09	182,16	275,78	0,25	2,41	22,21	30,43	39,64	58,6	5,3	11,06
79	E2.A.98.S.2			0,22	7,18	165,74	128,27	142,48	248,24	0,28	2,86	30,01	36,49	34,94	58,7	5,3	11,08
80	E2.A.98.S.3			0,20	6,24	154,36	125,96	156,02	239,80	0,26	2,52	25,59	37,21	32,80	60,1	5,3	11,34
81	E2.A.98.S.4			0,24	6,64	168,73	135,40	163,85	253,75	0,27	2,66	25,41	36,85	33,92	59,9	5	11,98
82	E2.A.98.D.1			0,21	5,85	150,51	99,05	95,97	158,53	0,20	2,50	22,76	40,44	38,73	53,8	4,7	11,45
83	E2.A.98.D.2			0,19	5,78	148,97	97,33	96,65	148,47	0,19	2,45	20,84	42,04	32,91	57,2	4,8	11,92
84	E2.A.98.D.3			0,17	5,77	138,45	82,48	79,51	128,01	0,18	2,30	20,22	37,12	43,51	59,9	4,8	12,48
85	E2.A.98.D.4			0,15	5,24	133,72	85,53	82,79	138,33	0,17	2,12	20,72	41,11	35,08	60,6	4,9	12,37
86	E2.A.147.T.1	150,84	2,99	0,26	7,66	181,89	133,89	156,02	211,37	0,23	2,63	29,03	36,06	38,91	48,2	5	9,64
87	E2.A.147.T.2	145,09	2,87	0,03	7,66	264,29	129,14	105,45	181,99	0,18	0,00	19,92	36,88	43,29	47,8	5,1	9,37
88	E2.A.147.T.3	138,93	2,93	0,26	7,52	181,46	122,00	133,05	192,26	0,21	2,85	25,91	35,85	39,03	45,1	4,9	9,20
89	E2.A.147.T.4	139,07	2,78	0,03	8,38	270,96	134,31	107,44	183,63	0,19	0,00	19,38	38,29	42,34	47,3	4,9	9,65
90	E2.A.147.S.1	128,74	3,20	0,25	6,36	167,84	135,57	173,45	282,39	0,28	2,63	28,69	35,63	41,14	60,3	5,3	11,38
91	E2.A.147.S.2	134,67	3,46	0,27	7,48	174,85	155,69	177,07	308,94	0,30	2,70	32,72	31,00	42,37	60,9	5,3	11,49
92	E2.A.147.S.3	137,84	3,32	0,21	8,65	338,96	135,76	160,34	278,16	0,28	0,98	26,71	37,85	40,84	63,2	5,2	12,15
93	E2.A.147.S.4	139,59	3,58	0,07	4,74	237,47	142,41	195,46	321,90	0,26	0,00	17,38	40,17	43,99	58,5	5,2	11,25
94	E2.A.147.D.1	119,72	2,61	0,22	6,54	164,45	90,73	94,90	163,56	0,24	2,82	29,18	39,05	39,51	64	5	12,80
95	E2.A.147.D.2	125,78	2,17	0,24	6,58	178,17	101,12	103,58	180,02	0,23	2,90	29,43	35,57	39,21	63,1	4,8	13,15
96	E2.A.147.D.3	119,83	2,52	0,24	6,74	200,51	108,97	111,07	195,76	0,26	3,25	32,29	38,23	40,35	62,6	4,9	12,78
97	E2.A.147.D.4	123,95	1,96	0,25	NA	167,58	98,83	110,41	177,68	0,21	2,52	24,24	39,83	41,77	63,8	5,1	12,51
98	E2.A.360.T.1	128,99	3,13	0,09	6,34	95,90	136,73	56,40	23,83	0,07	0,43	7,98	53,86	48,95	48,9	5,4	9,06
99	E2.A.360.T.2	119,60	3,02	0,11	6,71	100,18	148,70	59,54	24,57	0,06	0,41	7,14	52,67	50,87	48,3	5,3	9,11
100	E2.A.360.T.3	139,71	3,33	0,09	6,47	94,59	144,66	57,06	25,46	0,06	0,41	6,49	52,23	53,20	47	5,2	9,04
101	E2.A.360.T.4	132,43	3,18	0,08	7,70	105,40	140,92	53,26	23,55	0,07	0,42	8,75	51,26	46,19	48,7	4,8	10,15
102	E2.A.360.S.1	134,36	5,13	0,09	5,16	90,96	152,61	104,85	57,76	0,08	0,38	6,78	46,50	53,27	62,5	5,4	11,57
103	E2.A.360.S.2	126,22	5,05	0,10	5,74	103,18	159,75	106,32	59,64	0,09	0,45	7,22	45,52	51,56	61,2	5	12,24
104	E2.A.360.S.3	120,78	4,79	0,09	5,64	102,40	168,45	111,38	61,60	0,09	0,40	7,48	46,86	50,57	61,3	5,3	11,57
105	E2.A.360.S.4	129,22	5,34	0,09	5,46	91,99	164,69	112,46	65,02	0,09	0,36	8,50	49,29	49,22	64,7	5,5	11,76
106	E2.A.360.D.1	118,66	3,01	0,08	4,99	93,21	136,31	54,22	24,82	0,05	0,32	5,74	55,68	43,88	64,1	5	12,82
107	E2.A.360.D.2	122,86	3,02	0,09	5,48	98,64	140,54	55,57	25,70	0,06	0,40	6,16	53,86	50,28	65,9	5,1	12,92
108	E2.A.360.D.3	122,36	2,97	0,09	5,20	93,91	129,53	52,31	22,22	0,05	0,37	5,96	55,83	42,03	62,7	5	12,54
109	E2.A.360.D.4	117,12	2,80	0,10	5,96	102,44	140,07	54,31	23,57	0,06	NA	6,85	52,72	51,74	66,6	4,9	13,59

	ID	sol	moda	rep	tps	pH	TC	TN	NH4+	NO3-	B-glu	ARS	Xylo	Leu	DE	SS	Stot	Fetot	Pbtot
2	**E2.N.0.T.1**	Nitisol	UA	1	T0	6,40	1,18	0,23	63,74	1,68	50,400085	59,575376	32,518937	94,520548	8,93	2,69	774,72675	58799,333	20,671137
3	**E2.N.0.T.2**	Nitisol	UA	2	T0	6,43	1,08	0,21	51,96	1,86	53,600768	59,788755	NA	91,148577	8,14	2,83	902,92796	67697,611	20,520902
4	**E2.N.0.T.3**	Nitisol	UA	3	T0	6,49	1,17	0,22	61,13	1,55	58,081724	NA	NA	101,68599	8,17	2,68	857,4564	61230,95	20,748555
5	**E2.N.0.T.4**	Nitisol	UA	4	T0	6,47	1,17	0,21	64,90	1,18	49,973328	58,081724	24,766172	102,95047	6,57	2,64	807,97646	57272,166	22,29251
6	**E2.N.0.S.1**	Nitisol	BCS	1	T0	6,27	1,45	0,26	39,46	1,60	36,21201	44,193135	18,565774	20,029913	6,41	3,22	1327,103	58495,982	21,692523
7	**E2.N.0.S.2**	Nitisol	BCS	2	T0	6,35	1,50	0,24	38,23	1,51	70,842435	50,916373	28,313304	16,006397	6,56	2,89	1851,5361	61915,234	23,174355
8	**E2.N.0.S.3**	Nitisol	BCS	3	T0	6,34	1,15	0,24	39,28	1,49	57,471122	52,128262	30,585315	7,6416845	6,66	2,97	1390,129	56670,439	22,510216
9	**E2.N.0.S.4**	Nitisol	BCS	4	T0	6,34	1,47	0,22	39,15	1,55	NA	51,723434	27,719088	12,633892	6,43	2,94	1496,4913	49073,35	22,624177
10	**E2.N.0.D.1**	Nitisol	ACD	1	T0	6,38	0,37	0,23	NA	1,33	88,535969	100,59272	65,06595	0	2,46	3,14	711,28638	53221,965	22,304453
11	**E2.N.0.D.2**	Nitisol	ACD	2	T0	6,40	0,38	0,24	14,33	1,47	105,0488	75,814082	30,356121	0	2,59	2,88	873,12546	59425,814	21,862928
12	**E2.N.0.D.3**	Nitisol	ACD	3	T0	6,42	0,44	0,23	19,22	1,62	72,743282	81,852705	57,613025	0	2,55	2,95	707,87463	48864,117	21,45076
13	**E2.N.0.D.4**	Nitisol	ACD	4	T0	6,41	0,58	0,22	3,63	1,41	94,885855	94,491407	63,494961	0,6010601	3,95	2,93	893,80888	62955,036	22,957911
14	**E2.N.7.T.1**	Nitisol	UA	1	T7	6,30	0,99	0,34	41,48	2,54	49,759949	52,747253	21,849995	91,359326					
15	**E2.N.7.T.2**	Nitisol	UA	2	T7	6,27	1,02	0,34	47,67	2,09	37,810733	70,884455	30,17177	91,148577					
16	**E2.N.7.T.3**	Nitisol	UA	3	T7	6,28	1,03	0,33	47,96	2,07	45,705751	49,119812	31,523169	103,79347					
17	**E2.N.7.T.4**	Nitisol	UA	4	T7	6,25	1,03	0,32	44,11	2,61	48,693055	60,64227	31,380917	85,879874					
18	**E2.N.7.S.1**	Nitisol	BCS	1	T7	6,01	0,84	0,30	22,56	2,26	35,099859	48,913731	37,844025	8,868154					
19	**E2.N.7.S.2**	Nitisol	BCS	2	T7	6,14	0,62	0,31	21,46	2,12	31,079764	27,502041	10,953564	14,95763					
20	**E2.N.7.S.3**	Nitisol	BCS	3	T7	6,13	0,83	0,29	21,93	1,98	26,090948	12,251336	0,8020561	9,36271					
21	**E2.N.7.S.4**	Nitisol	BCS	4	T7	6,20	0,84	0,30	20,52	2,36	34,362213	65,123201	16,516868	4,9923211					
22	**E2.N.7.D.1**	Nitisol	ACD	1	T7	6,15	0,68	0,28	7,25	2,04	83,321879	62,111378	24,457169	0					
23	**E2.N.7.D.2**	Nitisol	ACD	2	T7	6,15	0,54	0,30	NA	NA	103,49764	63,826714	25,09691	0					
24	**E2.N.7.D.3**	Nitisol	ACD	3	T7	6,18	0,36	0,31	0,00	2,12	67,377322	36,21201	14,571879	0					
25	**E2.N.7.D.4**	Nitisol	ACD	4	T7	6,20	0,37	0,31	7,80	2,18	41,924155	76,272235	29,684647	0					
26	**E2.N.14.T.1**	Nitisol	UA	1	T14	6,28	1,16	0,38	15,57	2,78	58,508482	58,081724	33,514705	83,245522	8,65				
27	**E2.N.14.T.2**	Nitisol	UA	2	T14	6,20	1,18	0,38	17,29	2,56	72,378107	63,842953	38,849177	95,187917	9,12				
28	**E2.N.14.T.3**	Nitisol	UA	3	T14	6,24	1,12	0,39	33,42	1,22	71,311213	55,307799	32,376685	68,727315	9,47				
29	**E2.N.14.T.4**	Nitisol	UA	4	T14	6,24	1,18	0,35	14,08	2,70	71,097834	61,069028	37,711156	83,245522	8,86				
30	**E2.N.14.S.1**	Nitisol	BCS	1	T14	6,25	0,87	0,35	0,00	2,43	46,538477	39,969174	33,566211	15,465293	9,49				
31	**E2.N.14.S.2**	Nitisol	BCS	2	T14	6,15	0,86	0,34	0,00	2,27	21,924474	33,994519	16,643127	23,782022	9,38				
32	**E2.N.14.S.3**	Nitisol	BCS	3	T14	6,19	0,87	0,34	7,32	2,29	52,940528	33,627575	19,878237	14,321844	7,64				
33	**E2.N.14.S.4**	Nitisol	BCS	4	T14	6,18	0,79	0,33	0,00	2,36	40,729982	33,627575	11,770378	11,787954	7,47				
34	**E2.N.14.D.1**	Nitisol	ACD	1	T14	6,42	0,40	0,36	0,00	2,23	95,280278	80,911898	50,183519	0	2,21				
35	**E2.N.14.D.2**	Nitisol	ACD	2	T14	6,32	NA	NA	0,00	2,18	76,019839	76,731434	48,198478	0	2,33				
36	**E2.N.14.D.3**	Nitisol	ACD	3	T14	6,29	0,66	0,32	0,00	2,16	73,97426	72,186094	55,503416	0	2,97				
37	**E2.N.14.D.4**	Nitisol	ACD	4	T14	6,49	NA	NA	NA	NA	NA	NA	NA	0	1,72				

	A	B	C	D	E	F	G	H	I	J	K	L	M	N	O	P	Q	R	S
1	**ID**	**sol**	**moda**	**rep**	**tps**	**pH**	**TC**	**TN**	**NH4+**	**NO3-**	**B-glu**	**ARS**	**Xylo**	**Leu**	**DE**	**SS**	**Stot**	**Fetot**	**Pbtot**
38	**E2.N.21.T.1**	Nitisol	UA	1	T21		1,00	0,42	0,00	1,71	93,715993	87,954764	48,380099	124,45849		2,65			
39	**E2.N.21.T.2**	Nitisol	UA	2	T21		0,92	0,41	0,00	2,78	65,976742	73,871759	44,823785	125,39515		2,87			
40	**E2.N.21.T.3**	Nitisol	UA	3	T21		1,02	0,43	0,00	1,77	70,457698	78,779473	41,694228	128,43929		2,72			
41	**E2.N.21.T.4**	Nitisol	UA	4	T21		0,98	0,41	0,00	2,76	83,473808	75,57879	40,982965	93,080436		2,66			
42	**E2.N.21.S.1**	Nitisol	BCS	1	T21		0,81	0,38	8,14	2,50	74,155833	81,930564	41,10526	11,023674		2,99			
43	**E2.N.21.S.2**	Nitisol	BCS	2	T21		0,80	0,38	0,00	1,52	56,190447	71,483865	35,306918	18,189712		2,83			
44	**E2.N.21.S.3**	Nitisol	BCS	3	T21		0,83	0,38	0,00	2,55	57,008334	43,575644	30,237712	22,392002		3,01			
45	**E2.N.21.S.4**	Nitisol	BCS	4	T21		0,85	0,39	0,00	2,52	61,992165	60,73376	39,179911	22,392002		2,84			
46	**E2.N.21.D.1**	Nitisol	ACD	1	T21		0,42	0,39	0,00	1,46	132,49385	145,80981	124,60222	0		3,04			
47	**E2.N.21.D.2**	Nitisol	ACD	2	T21		0,42	0,38	9,39	1,34	113,77565	125,96781	82,47448	0		2,92			
48	**E2.N.21.D.3**	Nitisol	ACD	3	T21		0,44	0,36	0,00	2,07	117,57016	131,79807	86,314063	0		3,01			
49	**E2.N.21.D.4**	Nitisol	ACD	4	T21		0,46	0,37	0,00	1,31	101,06814	113,11774	80,468915	0		2,92			
50	**E2.N.28.T.1**	Nitisol	UA	1	T28	6,43	1,41	0,28	6,64	2,20	72,804865	66,616878	31,949927	140,61585					
51	**E2.N.28.T.2**	Nitisol	UA	2	T28	6,35	1,24	0,32	**NA**	2,48	NA	NA	NA	140,38169					
52	**E2.N.28.T.3**	Nitisol	UA	3	T28	6,46	1,44	0,28	12,01	2,07	74,725275	80,699883	34,866105	145,53331					
53	**E2.N.28.T.4**	Nitisol	UA	4	T28	6,43	1,43	0,31	18,03	2,18	107,15886	58,508482	41,623102	142,02084					
54	**E2.N.28.S.1**	Nitisol	BCS	1	T28	6,24	0,95	0,37	0,00	2,51	32,895926	41,493959	18,565774	42,245709					
55	**E2.N.28.S.2**	Nitisol	BCS	2	T28	6,42	**NA**	**NA**	0,00	2,49	55,811151	39,211518	14,896715	47,29279					
56	**E2.N.28.S.3**	Nitisol	BCS	3	T28	6,23	0,93	0,38	2,34	2,46	57,054777	44,193135	16,643127	19,257217					
57	**E2.N.28.S.4**	Nitisol	BCS	4	T28	6,28	0,94	0,39	0,00	2,55	57,471122	52,533959	28,462614	22,255459					
58	**E2.N.28.D.1**	Nitisol	ACD	1	T28	6,27	0,53	0,38	1,05	2,30	119,56557	79,508799	61,284068	0					
59	**E2.N.28.D.2**	Nitisol	ACD	2	T28	6,23	0,49	0,37	0,00	2,29	111,20913	92,993256	56,132503	0					
60	***E2.N.28.D.3***	Nitisol	ACD	3	T28	6,27	0,44	0,38	4,54	2,30	128,1637	117,76346	56,764832	0					
61	**E2.N.28.D.4**	Nitisol	ACD	4	T28	6,31	0,41	0,37	8,01	2,26	128,9048	94,491407	68,49901	0					
62	**E2.N.35.T.1**	Nitisol	UA	1	T35		1,19	0,50			73,231623	77,4992	40,271702	136,63505	6,48				
63	**E2.N.35.T.2**	Nitisol	UA	2	T35		1,10	0,46			72,804865	84,96746	49,944877	143,65999	7,00				
64	**E2.N.35.T.3**	Nitisol	UA	3	T35		1,21	0,49			64,269711	72,164728	44,183648	131,7176	8,29				
65	**E2.N.35.T.4**	Nitisol	UA	4	T35		1,25	0,47			100,54412	101,61101	46,957573	140,85002	8,10				
66	**E2.N.35.S.1**	Nitisol	BCS	1	T35		0,95	0,42			80,443121	57,471122	24,111572	21,952366	5,87				
67	**E2.N.35.S.2**	Nitisol	BCS	2	T35		0,93	0,42			78,578764	60,411074	18,306265	17,200852	4,65				
68	**E2.N.35.S.3**	Nitisol	BCS	3	T35		0,87	0,40			43,805113	70,396561	30,585315	31,703086	6,20				
69	**E2.N.35.S.4**	Nitisol	BCS	4	T35		0,94	0,39			83,272102	74,445865	35,675438	0	6,55				
70	**E2.N.35.D.1**	Nitisol	ACD	1	T35		0,43	0,38			132,22691	151,0188	64,840329	0	3,75				
71	**E2.N.35.D.2**	Nitisol	ACD	2	T35		0,43	0,39			118,81128	132,59474	76,881682	0	2,26				
72	**E2.N.35.D.3**	Nitisol	ACD	3	T35		0,70	0,41			124,81582	140,26018	76,881682	0	4,16				
73	**E2.N.35.D.4**	Nitisol	ACD	4	T35		0,44	0,39			128,1637	152,43654	85,564027	0	2,63				

	A	B	C	D	E	F	G	H	I	J	K	L	M	N	O	P	Q	R	S
1	**ID**	**sol**	**moda**	**rep**	**tps**	**pH**	**TC**	**TN**	**NH4+**	**NO3-**	**B-glu**	**ARS**	**Xylo**	**Leu**	**DE**	**SS**	**Stot**	**Fetot**	**Pbtot**
74	**E2.N.63.T.1**	Nitisol	UA	1	T63	6,55	1,12	0,19	67,91	1,27	65,336605	68,964046	46,317437	146,70413	6,62	2,84			
75	**E2.N.63.T.2**	Nitisol	UA	2	T63	6,15	1,08	0,99	8,17	3,08	74,938654	67,683773	46,388563	126,80014	7,69	2,64			
76	**E2.N.63.T.3**	Nitisol	UA	3	T63	7,25	1,99	0,20	239,82	0,03	83,900565	74,725275	48,949109	134,76174	8,72	3,00			
77	**E2.N.63.T.4**	Nitisol	UA	4	T63	7,20	1,89	0,18	226,95	0,03	84,113944	61,709165	47,811089	144,59665	8,76	2,98			
78	**E2.N.63.S.1**	Nitisol	BCS	1	T63	6,61	1,06	0,13	74,42	0,47	77,191683	78,578764	16,643127	42,955357	7,94	2,86			
79	**E2.N.63.S.2**	Nitisol	BCS	2	T63	6,08	0,62	0,44	0,00	2,85	60,834751	54,165491	19,74586	39,09777	6,30	2,82			
80	**E2.N.63.S.3**	Nitisol	BCS	3	T63	6,57	1,00	0,16	87,18	0,65	79,508799	68,623043	32,928785	49,514018	6,71	2,73			
81	**E2.N.63.S.4**	Nitisol	BCS	4	T63	6,91	1,35	0,15	149,10	0,05	81,381758	100,07752	54,919737	58,371481	5,90	2,82			
82	**E2.N.63.D.1**	Nitisol	ACD	1	T63	6,31	**NA**	**NA**	**NA**	**NA**	134,0635	112,79602	59,327079	0	6,39	2,88			
83	**E2.N.63.D.2**	Nitisol	ACD	2	T63	6,51	0,61	0,17	43,01	0,96	131,85882	87,105919	74,917923	0	3,70	2,94			
84	**E2.N.63.D.3**	Nitisol	ACD	3	T63	6,47	0,54	0,19	41,51	1,08	123,32104	99,050848	70,133028	0	1,93	2,99			
85	**E2.N.63.D.4**	Nitisol	ACD	4	T63	6,69	**NA**	**NA**	**NA**	**NA**	117,29949	101,62686	51,188539	0	2,66	2,81			
86	**E2.N.98.T.1**	Nitisol	UA	1	T98	6,52	1,11	0,21	19,52	0,78	94,569508	85,607596	52,57655	124,22433	8,37				
87	**E2.N.98.T.2**	Nitisol	UA	2	T98	6,22	0,84	0,54	0,00	1,84	93,289235	76,005548	49,660372	126,33181	8,60				
88	**E2.N.98.T.3**	Nitisol	UA	3	T98	6,18	0,88	0,60	0,00	3,13	96,06316	55,307799	42,476617	140,38169	10,68				
89	**E2.N.98.T.4**	Nitisol	UA	4	T98	6,18	0,90	0,56	**NA**	2,01	96,276539	76,859063	49,589246	108,5353	9,25				
90	**E2.N.98.S.1**	Nitisol	BCS	1	T98	6,13	0,65	0,48	**NA**	1,84	91,505744	74,900901	27,276574	56,006763	7,54				
91	**E2.N.98.S.2**	Nitisol	BCS	2	T98	6,71	0,84	0,18	21,27	0,90	140,65589	113,34246	58,099225	62,810688	8,46				
92	**E2.N.98.S.3**	Nitisol	BCS	3	T98	6,16	0,66	0,50	0,00	1,60	112,25094	82,324742	46,40879	42,600068	7,86				
93	**E2.N.98.S.4**	Nitisol	BCS	4	T98	6,12	0,66	0,49	0,00	2,45	105,28619	59,145592	43,270116	57,97463	8,61				
94	**E2.N.98.D.1**	Nitisol	ACD	1	T98	6,67	0,63	0,19	17,52	1,06	188,30803	149,46074	111,85616	0	2,14				
95	**E2.N.98.D.2**	Nitisol	ACD	2	T98	6,40	0,44	0,33	7,25	1,69	148,88487	132,76705	78,624079	0	2,15				
96	**E2.N.98.D.3**	Nitisol	ACD	3	T98	6,64	0,65	0,18	20,25	0,93	155,26037	133,96331	103,24573	0	3,40				
97	**E2.N.98.D.4**	Nitisol	ACD	4	T98	6,69	0,57	0,18	16,37	0,66	158,76848	143,76279	86,888857	0	2,51				
98	**E2.N.147.T.1**	Nitisol	UA	1	T147	6,24	0,74	0,42	8,06	3,08	92,862477	60,64227	47,59771	129,14179	8,74	2,62	792,75772	78291,197	83,137566
99	**E2.N.147.T.2**	Nitisol	UA	2	T147	7,43	1,36	0,18	271,98	0,03	64,909847	64,909847	42,689996	115,32607	10,57	3,06	885,57376	68720,579	78,708793
100	**E2.N.147.T.3**	Nitisol	UA	3	T147	6,39	0,79	0,29	0,00	1,89	73,231623	66,403499	44,823785	129,14179	**NA**	2,59	719,58086	71861,272	76,416366
101	**E2.N.147.T.4**	Nitisol	UA	4	T147	7,47	1,58	0,20	321,39	0,02	60,002134	62,349301	41,26747	93,782929	10,34	2,79	885,11231	77434,963	87,219551
102	**E2.N.147.S.1**	Nitisol	BCS	1	T147	6,24	0,73	0,32	0,00	2,51	52,533959	36,957257	45,473074	60,372379	8,84	2,49	1373,0035	83573,555	89,685385
103	**E2.N.147.S.2**	Nitisol	BCS	2	T147	6,25	**NA**	**NA**	**NA**	**NA**	62,111378	44,193135	NA	34,678113	4,88	3,10	1365,1431	63254,408	80,842336
104	**E2.N.147.S.3**	Nitisol	BCS	3	T147	6,60	0,80	0,14	56,37	0,73	73,991861	46,538477	45,101756	18,961341	9,81	2,95	1159,8711	66870,843	73,96551
105	**E2.N.147.S.4**	Nitisol	BCS	4	T147	6,85	0,86	0,11	47,23	0,23	67,742223	42,645913	38,523615	25,327331	9,04	2,83	1180,2896	58252,919	77,218875
106	**E2.N.147.D.1**	Nitisol	ACD	1	T147	6,75	0,60	0,08	8,46	0,43	114,64349	105,28619	109,96866	0	3,11	2,80	774,15606	78926,775	83,750287
107	**E2.N.147.D.2**	Nitisol	ACD	2	T147	6,74	0,65	0,09	0,00	0,49	118,43373	119,44437	127,50835	0	4,81	2,85	809,75199	68160,558	85,248543
108	**E2.N.147.D.3**	Nitisol	ACD	3	T147	6,75	0,54	0,08	0,00	0,42	81,303211	94,491407	92,595521	0	2,52	2,95	824,54094	79930,222	91,933196
109	**E2.N.147.D.4**	Nitisol	ACD	4	T147	6,55	0,59	0,15	0,00	1,10	121,07095	98,029113	103,24573	0	1,73	2,95	750,87438	64383,709	78,663567
110	**E2.N.360.T.1**	Nitisol	UA	1	T360	7,33	1,14	0,28	127,34	1,37	54,240905	47,62616	25,690814	64,044023	6,56	3,22	508,16127	38873,138	15,122974
111	**E2.N.360.T.2**	Nitisol	UA	2	T360	7,11	**NA**	0,10	9,27	0,39	51,253601	53,814147	31,523169	94,017094	6,10	2,72	598,29021	38832,446	17,25305
112	**E2.N.360.T.3**	Nitisol	UA	3	T360	6,36	**NA**	**NA**	**NA**	**NA**	NA	NA	NA	NA	9,76	3,22	542,864	43418,192	14,907853
113	**E2.N.360.T.4**	Nitisol	UA	4	T360	6,76	1,01	0,25	79,50	1,43	56,374693	49,333191	27,753476	71,771455	5,12	3,03	648,38562	46373,347	17,509806
114	**E2.N.360.S.1**	Nitisol	BCS	1	T360	6,46	0,50	0,70	9,03	3,80	45,362102	4,0251032	14,528343	31,376683	6,07	2,35	979,24423	38901,256	14,317978
115	**E2.N.360.S.2**	Nitisol	BCS	2	T360	6,22	0,66	0,72	0,00	3,85	21,581715	13,880554	10,953564	51,014863	2,08	3,07	927,5544	36279,578	15,341263
116	**E2.N.360.S.3**	Nitisol	BCS	3	T360	6,51	0,69	0,14	0,00	0,78	27,856583	15,856455	18,95689	34,344192	6,04	2,89	1076,3339	44802,667	16,193863
117	**E2.N.360.S.4**	Nitisol	BCS	4	T360	6,71	**NA**	0,07	8,95	0,18	29,281894	25,739934	25,532995	32,358363	5,98	2,78	975,71585	35384,746	15,436205
118	**E2.N.360.D.1**	Andosol	ACD	1	T360	6,47	0,35	0,44	0,00	2,54	56,911076	NA	NA	0	2,47	2,61	552,52315	42014,849	15,165576
119	**E2.N.360.D.2**	Andosol	ACD	2	T360	6,73	0,63	0,10	7,99	0,47	69,033901	50,514134	72,264602	0	2,19	3,15	570,11627	41543,106	14,921427
120	**E2.N.360.D.3**	Andosol	ACD	3	T360	6,59	0,50	0,29	0,00	1,72	82,111567	38,456992	53,635363	NA	1,87	2,60	578,88852	44848,993	14,777307
121	**E2.N.360.D.4**	Andosol	ACD	4	T360	6,93	0,50	0,06	8,84	0,10	59,861361	47,326877	43,566059	NA	1,51	2,79	599,71927	41307,716	15,162297

	A	T	U	V	W	X	Y	Z	AA	AB	AC	AD	AE	AF	AG	AH	AI
1	**ID**	**Zntot**	**Astot**	**As**	**Cu**	**Fe**	**K**	**Mg**	**Na**	**Ni**	**Pb**	**Zn**	**Phos**	**CEC**	**Corg**	**N**	**C/N**
2	**E2.N.0.T.1**	107,12965	2,0506576	0,2091833	6,1305489	149,30183	195,27394	154,70355	66,095253	0,1804972	2,5600208	26,826962	194,14	44,419599	51,2	5	10,24
3	**E2.N.0.T.2**	116,48881	2,036431	0,2253099	6,682633	155,27714	211,98078	169,02329	70,370799	0,1883694	2,5774521	31,078727	215,21	44,717206	46,9	5	9,38
4	**E2.N.0.T.3**	117,19054	2,2394384	0,2072394	6,9735395	165,39626	216,48378	170,67828	71,175269	0,1979812	2,6211062	33,582282	214,80	41,705911	50,8	5,1	9,96
5	**E2.N.0.T.4**	136,91204	2,2845668	0,2273552	7,5140675	172,10635	234,00696	179,49667	77,333864	0,2248617	2,8904923	37,120434	215,46	42,307889	49,3	4,8	10,27
6	**E2.N.0.S.1**	120,97472	3,9648183	0,2034224	5,7916858	151,14404	223,82385	202,90252	107,18808	0,2380958	2,5464922	30,725136	201,77	43,460131	62,8	5,4	11,63
7	**E2.N.0.S.2**	132,07225	4,2560769	0,1958745	5,8868055	152,32551	246,17069	223,48502	122,14859	0,2588413	2,5328648	37,053052	206,58	44,231988	65,9	5,3	12,43
8	**E2.N.0.S.3**	126,84253	4,5101509	0,2190993	6,3251301	163,0659	261,88051	238,5276	126,59606	0,2713406	2,7778473	35,242313	206,36	44,506388	65,9	5,3	12,43
9	**E2.N.0.S.4**	122,34927	4,441282	0,2259443	6,4202488	165,89898	275,79787	249,67031	133,80673	0,2820536	2,7384175	36,560678	211,29	43,189829	68,7	5,6	12,27
10	**E2.N.0.D.1**	119,91563	1,9344324	0,1466463	4,5327236	132,40654	179,43687	154,94896	70,299192	0,1656884	2,2323467	28,832628	217,89	38,233518	67,1	4,9	13,69
11	**E2.N.0.D.2**	110,28949	1,8427197	0,1689211	4,9423311	140,59623	189,11878	158,344	71,644895	0,1704767	2,4597474	28,64143	211,29	41,380101	64,8	4,8	13,50
12	**E2.N.0.D.3**	115,42526	1,8707578	0,1902483	5,1688991	147,98978	214,14022	180,22601	79,874693	0,1871542	2,4186056	30,36247	222,57	42,33743	71,8	5,1	14,08
13	**E2.N.0.D.4**	110,68974	2,0152358	0,185593	5,2969832	152,67466	209,08633	169,78706	74,830107	0,1803984	2,4325722	29,305282	212,61	37,559566	57,4	4,8	11,96
14	**E2.N.7.T.1**			0,1780424	5,9098001	156,31837	194,5048	159,18515	59,929357	0,2712606	2,3248121	27,157308	183,70	44,091651	46,7	5	9,34
15	**E2.N.7.T.2**			0,2124672	6,7648934	171,67651	227,44323	179,70559	68,248789	0,1922958	2,6919073	41,486255	189,25	44,99821	52,9	5,3	9,98
16	**E2.N.7.T.3**			0,214923	6,6437707	169,43969	236,9782	181,53131	70,54746	0,1840403	2,6821331	28,864768	191,92	42,290519	50,5	5	10,10
17	**E2.N.7.T.4**			0,2207911	6,9163017	186,0301	254,76812	189,74353	75,624222	0,1959975	3,240345	29,749118	181,70	40,776859	45	4,5	10,00
18	**E2.N.7.S.1**			0,202628	6,2315099	170,91888	263,60203	231,78452	110,69171	0,2571499	2,7128897	37,755294	181,25	42,2112	58,6	5	11,72
19	**E2.N.7.S.2**			0,2004581	5,7925603	159,10986	239,06939	214,18309	105,15285	0,2387758	2,4367139	28,629074	189,47	40,05051	55,3	5	11,06
20	**E2.N.7.S.3**			0,2133885	6,4378751	177,04945	275,40696	242,5629	121,18431	0,2713605	2,8514943	35,187787	199,47	45,214352	62,2	5,2	11,96
21	**E2.N.7.S.4**			0,1836991	5,7729741	159,54847	238,83419	211,15145	99,570646	0,2423574	2,6060676	31,061204	202,44	43,26856	56,4	4,9	11,51
22	**E2.N.7.D.1**			0,1784261	5,1840228	158,78175	198,20814	159,79694	64,222394	0,1873868	2,7435891	24,31477	208,41	39,198871	49,1	4,4	11,16
23	**E2.N.7.D.2**			0,2068224	5,6997829	178,68003	225,66709	185,63418	75,720593	0,1933886	2,5880706	29,151043	207,97	45,948556	52,9	4,6	11,50
24	**E2.N.7.D.3**			0,1787899	4,2677759	145,24073	181,73917	150,83425	62,850545	0,1649512	2,5406103	24,69326	203,91	44,72317	65,3	4,7	13,89
25	**E2.N.7.D.4**			0,1650417	4,8704898	153,56135	189,66707	152,76459	64,629289	0,1508081	2,2337758	23,780273	188,36	39,284782	56,4	4,3	13,12
26	**E2.N.14.T.1**			0,2100153	7,207141	167,29536	224,04741	191,16086	72,181375	0,1998173	2,7198321	28,446079	211,87	45,495027	50,5	5,1	9,90
27	**E2.N.14.T.2**			0,2038096	6,6236979	163,28979	215,14922	181,00311	68,542082	0,1872644	2,5936208	27,213236	75,22	43,640092	51,1	5,1	10,02
28	**E2.N.14.T.3**			0,2069132	6,6430937	167,6136	217,58536	181,13815	69,371639	0,1914045	2,6103022	26,218125	66,81	40,575778	46,2	4,6	10,04
29	**E2.N.14.T.4**			0,1931162	6,9687355	167,80428	233,14355	188,27104	72,453706	0,1892224	2,8042531	30,229893	106,18	41,547445	49,7	5	9,94
30	**E2.N.14.S.1**			0,1770056	5,454203	152,46226	205,24937	194,213	89,521185	0,2115168	2,2801282	23,657386	119,76	43,20605	54,7	4,9	11,16
31	**E2.N.14.S.2**			0,1735773	5,3091166	148,77855	224,46827	215,45488	102,691	0,232759	2,3582557	26,527246	91,82	44,506388	58,6	5,3	11,06
32	**E2.N.14.S.3**			0,1862662	5,5314334	161,22494	228,92056	226,10562	108,27184	0,2784467	2,5195896	31,577249	100,85	41,39469	63,7	5,3	12,02
33	**E2.N.14.S.4**			0,1951052	6,1272372	160,51903	237,9715	225,35361	107,97572	0,2450288	2,5568414	30,476011	100,66	42,279243	58,6	5	11,72
34	**E2.N.14.D.1**			0,183558	6,2538757	156,83329	188,87456	157,57164	65,680477	0,1718599	2,4395974	26,806107	145,05	41,004223	58,5	4,7	12,45
35	**E2.N.14.D.2**			0,1926987	5,1985987	160,50517	197,23032	167,48644	69,12383	0,2087163	2,472173	27,798736	138,72	40,441364	55,6	4,8	11,58
36	**E2.N.14.D.3**			*0,17226*	*5,351492*	*137,5992*	*171,2673*	*157,3583*	*64,97751*	*0,16253*	*2,111138*	*23,34048*	*160,15*	41,635742	NA	NA	NA
37	**E2.N.14.D.4**			0,1698323	4,1919414	132,18528	183,0913	165,75866	71,001487	0,1534988	1,9894037	23,085401	*123,06*	38,407977	59,6	5	11,92

	A	T	U	V	W	X	Y	Z	AA	AB	AC	AD	AE	AF	AG	AH	AI
1	**ID**	**Zntot**	**Astot**	**As**	**Cu**	**Fe**	**K**	**Mg**	**Na**	**Ni**	**Pb**	**Zn**	**Phos**	**CEC**	**Corg**	**N**	**C/N**
38	**E2.N.21.T.1**																
39	**E2.N.21.T.2**																
40	**E2.N.21.T.3**																
41	**E2.N.21.T.4**																
42	**E2.N.21.S.1**																
43	**E2.N.21.S.2**																
44	**E2.N.21.S.3**																
45	**E2.N.21.S.4**																
46	**E2.N.21.D.1**																
47	**E2.N.21.D.2**																
48	**E2.N.21.D.3**																
49	**E2.N.21.D.4**																
50	**E2.N.28.T.1**			0,2139104	6,7683392	161,02269	196,73872	175,71333	70,033697	0,19006	2,5947405	30,683733	200,58	45,948556	39,2	5	7,84
51	**E2.N.28.T.2**			0,217082	6,807451	164,16819	203,39983	181,81401	72,239743	0,1926176	2,7909711	29,509989	205,69	41,512518	44,9	4,7	9,55
52	**E2.N.28.T.3**			0,19971	6,6378341	157,40408	195,77385	172,19075	69,583266	0,1831268	2,5355594	29,221761	191,92	44,512664	41,5	4,9	8,47
53	**E2.N.28.T.4**			0,1931929	6,5287102	154,01843	200,44409	177,5453	71,240851	0,1886891	2,4232454	29,027857	203,98	42,959668	41,2	4,7	8,77
54	**E2.N.28.S.1**			0,1938819	5,1343008	144,50058	201,49452	202,9623	93,807156	0,2104866	2,2602201	26,490196	212,54	42,722425	47,8	4,9	9,76
55	**E2.N.28.S.2**			0,1782493	5,9099162	147,32127	209,79322	203,34514	95,326194	0,2078734	2,3636978	34,430313	209,69	40,015583	48,9	4,9	9,98
56	**E2.N.28.S.3**			0,1960279	5,5566525	160,30912	237,40787	241,34345	116,08448	0,2374058	2,4629926	29,361393	199,38	40,664992	47,4	5,3	8,94
57	**E2.N.28.S.4**			0,2016753	5,4059862	144,74844	202,02386	201,35474	92,026002	0,1971944	2,3459035	27,322085	206,36	42,031532	73,4	5,2	14,12
58	**E2.N.28.D.1**			0,1943076	5,8465115	168,11803	222,57961	191,93992	81,529292	0,1890238	2,6472886	29,639517	219,68	42,16971	41,6	4,8	8,67
59	**E2.N.28.D.2**			0,1560361	4,6340808	138,76828	165,92765	154,58871	63,616604	0,1567089	2,08438	22,225825	193,58	41,340639	**75,7**	4,5	16,82
60	***E2.N.28.D.3***			*0,15*	*5,306288*	*155,3457*	*187,1548*	*161,5677*	*64,56237*	*0,174187*	*2,491208*	*28,24754*	*212,13*	40,926103	NA	NA	NA
61	**E2.N.28.D.4**			0,1669681	4,5997881	139,94958	178,48611	163,50489	69,667936	0,1670008	2,117619	24,575999	183,19	35,667344	47	4,7	10,00
62	**E2.N.35.T.1**																
63	**E2.N.35.T.2**																
64	**E2.N.35.T.3**																
65	**E2.N.35.T.4**																
66	**E2.N.35.S.1**																
67	**E2.N.35.S.2**																
68	**E2.N.35.S.3**																
69	**E2.N.35.S.4**																
70	**E2.N.35.D.1**																
71	**E2.N.35.D.2**																
72	**E2.N.35.D.3**																
73	**E2.N.35.D.4**																

	A	T	U	V	W	X	Y	Z	AA	AB	AC	AD	AE	AF	AG	AH	AI
1	**ID**	**Zntot**	**Astot**	**As**	**Cu**	**Fe**	**K**	**Mg**	**Na**	**Ni**	**Pb**	**Zn**	**Phos**	**CEC**	**Corg**	**N**	**C/N**
74	**E2.N.63.T.1**			0,1890311	7,1594374	158,9406	193,15184	163,28295	69,102231	0,1984084	2,6482772	56,694514	179,92208	46,787431	45,7	4,7	9,72
75	**E2.N.63.T.2**			0,2161902	6,7253824	155,46826	221,29892	209,83506	78,022327	0,2007203	2,4802333	28,827268	188,80712	43,389963	42,5	5,1	8,33
76	**E2.N.63.T.3**			0,0520604	6,6353238	242,05827	185,58958	128,4735	58,722921	0,2163188	0	26,336972	153,04483	46,233501	62,1	4,7	13,21
77	**E2.N.63.T.4**			0,0723379	7,7940373	252,20875	191,72298	131,92729	61,940484	0,1677864	0	28,991417	171,48129	47,835033	**24,2**	5,1	4,75
78	**E2.N.63.S.1**			0,176198	6,0496431	153,38024	173,75782	163,08154	82,568864	0,2259078	2,436195	31,456875	199,37818	46,480952	63,7	4,7	13,55
79	**E2.N.63.S.2**			0,1884361	5,7032733	158,14952	242,50907	263,55189	119,22697	0,2585739	2,8094094	35,379299	205,91082	45,006936	57	5,3	10,75
80	**E2.N.63.S.3**			0,2054047	6,6494209	173,13353	242,65655	221,83278	120,42717	0,3055912	2,8421378	41,503745	201,32936	47,806107	64,7	5,2	12,44
81	**E2.N.63.S.4**			0,1743208	6,7304629	184,90124	217,31382	175,61123	98,758679	0,222644	1,5652103	31,513777	190,01142	49,79384	59,5	4,9	12,14
82	**E2.N.63.D.1**			0,1625658	4,2013469	133,11122	168,41127	162,36353	67,663723	0,1634502	2,1649229	26,484154	197,81705	42,471804	63	4,9	12,86
83	**E2.N.63.D.2**			0,171818	4,7275876	144,24518	172,35886	148,80955	68,574223	0,1873969	2,385346	33,866942	187,91862	40,173617	80,6	4,7	17,15
84	**E2.N.63.D.3**			0,169453	4,7051673	142,31331	171,61296	145,98134	67,221029	0,1728717	2,2713042	29,940803	191,69476	40,081538	75	4,5	16,67
85	**E2.N.63.D.4**			0,1694752	4,4502588	141,95648	166,13713	141,52264	70,398574	0,1729725	2,1489303	35,864968	213,24098	44,363823	**15,7**	4,8	3,27
86	**E2.N.98.T.1**			0,0841356	9,1003411	106,25324	62,593219	27,43365	49,796451	0,096037	0,8311307	6,9203528	167,03467	43,391817	47,1	4,6	10,24
87	**E2.N.98.T.2**			0,08079	9,3428506	108,71348	65,097747	29,360486	52,800168	0,0908306	0,5727648	7,2937773	157,51263	45,481518	49,3	5	9,86
88	**E2.N.98.T.3**			0,0912709	8,3139315	95,209659	57,811097	26,955448	45,611672	0,0774779	0,4855135	5,9738262	196,95925	47,273975	46,4	4,7	9,87
89	**E2.N.98.T.4**			0,1152537	9,4260338	107,33864	75,258895	35,306682	63,363117	0,0890139	0,4935316	6,9487831	165,16186	46,848797	48,3	5,2	9,29
90	**E2.N.98.S.1**			0,0842898	7,6865049	93,458462	64,915754	38,544334	120,84368	0,1032762	0,4669573	6,2037786	158,08677	41,876071	55,1	4,9	11,24
91	**E2.N.98.S.2**			0,0694337	7,5816822	92,927644	65,398395	36,875022	115,59131	0,0956704	0,4292975	5,877826	161,87952	48,478769	58,2	4,9	11,88
92	**E2.N.98.S.3**			0,0638326	7,2290051	82,52789	62,397064	37,2846	115,90709	0,1003205	0,4005933	5,7084582	179,58887	49,199446	54,4	4,7	11,57
93	**E2.N.98.S.4**			0,0626168	7,2137155	83,173858	59,926871	34,959317	109,1925	0,0899955	0,3772757	5,5254589	191,50472	44,617076	53,5	4,9	10,92
94	**E2.N.98.D.1**			0,0637287	7,5013506	82,343362	59,810109	26,890009	49,987244	0,0686748	0,4075246	5,7359585	160,24763	37,969141	51	4,2	12,14
95	**E2.N.98.D.2**			0,064123	6,5955699	79,13312	52,473161	23,951909	43,044422	0,0610462	0,3489402	4,8547764	151,00257	43,74273	50,1	4,6	10,89
96	**E2.N.98.D.3**			0,0498536	6,7770525	77,863839	52,400485	24,427299	44,907339	0,0672867	0,3528692	4,8903037	161,0633	41,203701	46	4,2	10,95
97	**E2.N.98.D.4**			0,0569586	7,0680264	87,210644	57,813433	26,732544	49,383403	0,0663779	0,3159788	5,3291111	163,2139	38,257537	55	4,1	13,41
98	**E2.N.147.T.1**	129,57027	1,3463375	0,0666237	8,508063	99,842048	58,031496	26,846251	46,125999	0,0813483	0,4338744	5,9818499	155,35912	51,174667	47,9	5	9,58
99	**E2.N.147.T.2**	124,6015	2,1764631	0,0819796	7,9534891	96,003817	55,00294	25,219152	42,772785	0,073963	0,4025006	5,7650588	145,67126	49,125389	48,1	4,7	10,23
100	**E2.N.147.T.3**	123,61425	1,2418509	0,0758335	8,868759	103,61244	59,253512	27,255136	50,829708	0,0877963	0,465962	6,2405675	169,19557	52,413324	50,6	5	10,12
101	**E2.N.147.T.4**	135,36085	1,6076511	0,0818648	8,4207119	99,866781	59,730298	27,120846	45,492319	0,0705833	0,4453482	6,233563	126,18033	45,710008	51,6	4,8	10,75
102	**E2.N.147.S.1**	138,52568	3,1340991	0,0753293	7,3600871	89,828429	63,388336	37,4909	109,04652	0,0952341	0,4611001	6,9281233	172,35425	45,677554	58,5	4,9	11,94
103	**E2.N.147.S.2**	131,09553	1,6779509	0,0822138	7,9836225	90,428431	69,244572	40,183719	124,95803	0,1035537	0,460666	6,5533816	162,96676	42,008937	60,4	5,4	11,19
104	**E2.N.147.S.3**	122,82808	1,219289	0,0665733	8,0130918	97,703225	68,653627	40,637627	122,49217	0,1048794	0,4298972	6,4065694	150,60051	39,436003	62,5	4,8	13,02
105	**E2.N.147.S.4**	125,11694	1,9020327	0,0882785	8,1494845	102,63248	72,914747	43,236395	129,3849	0,1092176	0,4001942	6,654064	141,71349	43,917005	58,4	4,6	12,70
106	**E2.N.147.D.1**	134,41662	1,0713358	0,0521163	7,8792039	90,92214	67,387532	31,053505	66,077896	0,0746466	0,3968601	6,7235655	160,04843	40,227867	48,8	4,2	11,62
107	**E2.N.147.D.2**	131,22326	1,4752346	0,0726908	7,9174197	93,199407	77,26333	35,607683	68,42279	0,0749771	0,2883084	6,2923007	183,7537	42,049903	58,1	4,2	13,83
108	**E2.N.147.D.3**	143,6203	1,7091478	0,0513652	6,6714283	85,45791	61,286252	28,871517	52,454803	0,0653409	0,3414836	5,1467803	172,46858	41,11235	65,6	4,4	14,91
109	**E2.N.147.D.4**	123,2195	1,2971026	0,0590072	7,693919	96,756505	55,473011	25,432364	45,659481	0,0730381	0,4524147	5,9109154	175,73551	44,119279	54,5	4,3	12,67

	A	T	U	V	W	X	Y	Z	AA	AB	AC	AD	AE	AF	AG	AH	AI
1	**ID**	**Zntot**	**Astot**	**As**	**Cu**	**Fe**	**K**	**Mg**	**Na**	**Ni**	**Pb**	**Zn**	**Phos**	**CEC**	**Corg**	**N**	**C/N**
110	**E2.N.360.T.1**	105,68016	2,9723058	0,0699392	6,7879734	345,22412	293,14274	226,43903	81,861284	0,1428365	0	19,486344	181,06868	47,6145	49,1	4,7	10,45
111	**E2.N.360.T.2**	154,8794	3,5699169	0,1996111	11,993128	333,51752	248,25559	204,46521	78,125686	0,2509886	3,627212	50,731096	206,8491	44,653001	46,4	5,00	9,28
112	**E2.N.360.T.3**	111,29254	3,1838926	0,2430244	7,1429574	160,71498	318,98955	269,84713	89,766845	0,2150594	2,6745636	27,715324	213,83572	43,344228	45,6	4,8	9,50
113	**E2.N.360.T.4**	153,21318	3,6486108	0,1371736	8,3198213	361,92852	269,97583	221,58597	76,37472	0,3676827	0,3996534	31,923415	189,019	44,512664	47,1	4,7	10,02
114	**E2.N.360.S.1**	105,23823	5,0484939	0,2473578	4,8973855	143,89574	358,74592	384,79947	147,78898	0,2684782	2,3073899	28,548994	208,03223	43,26856	68,3	5,5	12,42
115	**E2.N.360.S.2**	114,58147	4,7490005	**NA**	**NA**	**NA**	**NA**	**NA**	**NA**	**NA**	**NA**	**NA**	203,15957	40,245762	59,9	5,6	10,70
116	**E2.N.360.S.3**	125,92465	5,59335	0,1658773	6,792751	324,96142	256,05326	259,46116	113,43338	0,2325081	2,9259647	28,463402	201,74827	46,315068	60,7	4,9	12,39
117	**E2.N.360.S.4**	119,89347	5,135864	0,2181045	5,5434739	154,05601	258,84329	240,06851	111,90171	0,2500439	2,5482068	35,747408	232,6117	47,10891	66,1	5	13,22
118	**E2.N.360.D.1**	109,20976	3,1698036	0,219345	4,6988943	144,21784	274,86352	247,46026	90,965684	0,189496	2,2174353	25,694084	207,73487	40,506099	56,4	4,5	12,53
119	**E2.N.360.D.2**	106,18168	3,3042365	0,1733493	8,0322514	349,19937	216,92775	179,9267	72,243667	0,2338462	3,212357	24,309325	206,48212	41,365942	56,8	3,5	16,23
120	**E2.N.360.D.3**	107,94346	3,0529116	0,2007486	5,2750104	145,48642	253,00758	214,22214	80,135764	0,1976991	2,3867605	29,047255	231,53802	39,913034	57,8	4,6	12,57
121	**E2.N.360.D.4**	123,58835	3,0920172	0,2400492	5,6610031	175,02925	260,79932	208,59398	91,930152	0,244853	3,3846773	45,32558	262,06145	44,355045	72,8	4,5	16,18

Sol: soil type (Nitisol or Andosol), moda: modality (BCS, UA, ACD), rep: duplication, TC= HWC, TN=HWN, DE= chlordecone environmental availability, SS= soil structural stability.
