# Supplementary figures and images for: Effects of biochar from algae (Sargassum spp.) on the fertility of two chlordecone contaminated West Indies soil
Source: PLoS One. 2025 Dec 30;20(12):e0338385. doi: 10.1371/journal.pone.0338385 (PMC12753066; doi:10.1371/journal.pone.0338385)

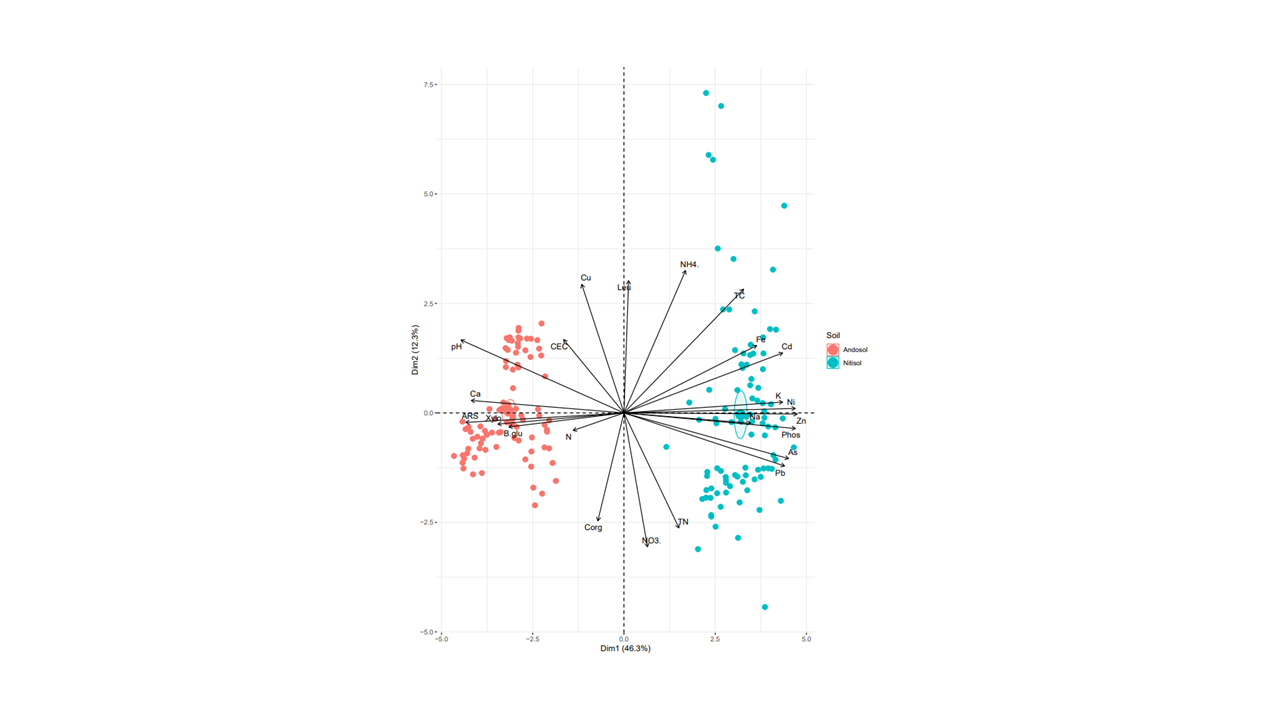

Supplement: S1 Fig — Points represent the coordinates of each sample according to the 2 dimensions of PCA1. (red: Andosol and blue: Nitisol). TC = HWC, TN = HWN. Confidence ellipses (95%) were plotted for each treatment. (TIF) [file pone.0338385.s007.tif]

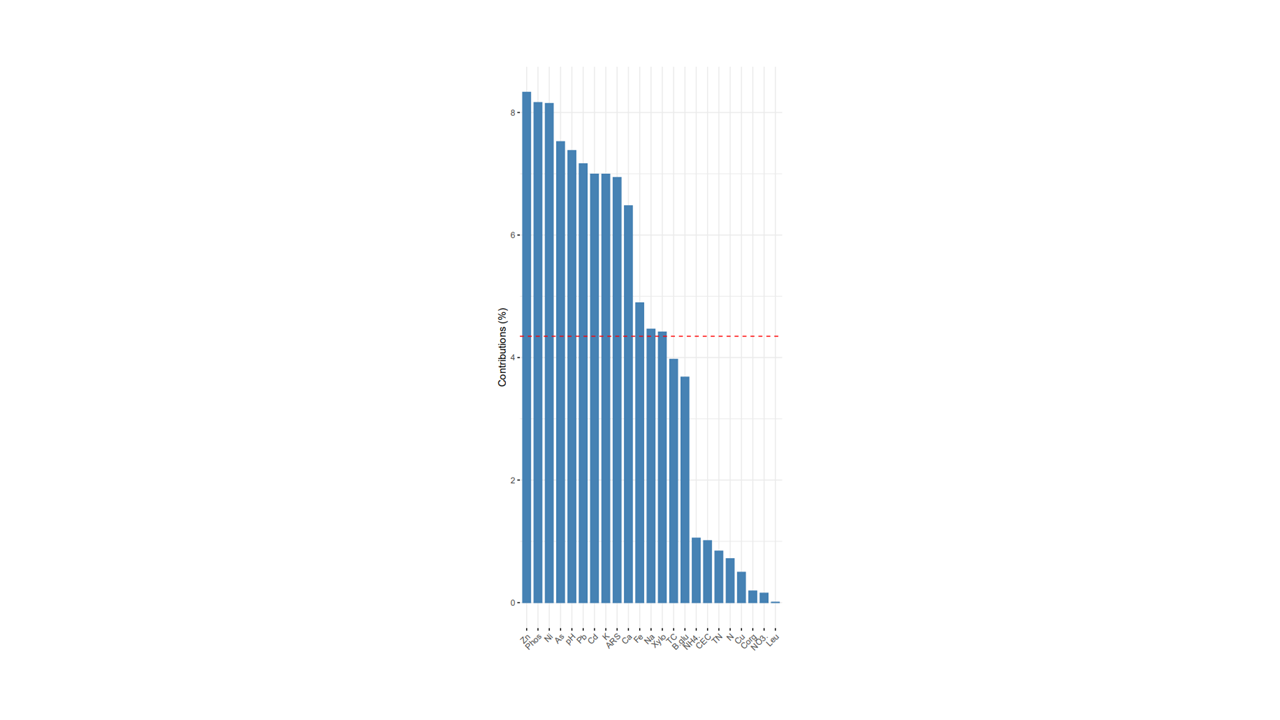

Supplement: S2 Fig — The red line represents the average contribution threshold if all variables had an equal contribution. TC = HWC, TN = HWN. (TIF) [file pone.0338385.s008.tif]

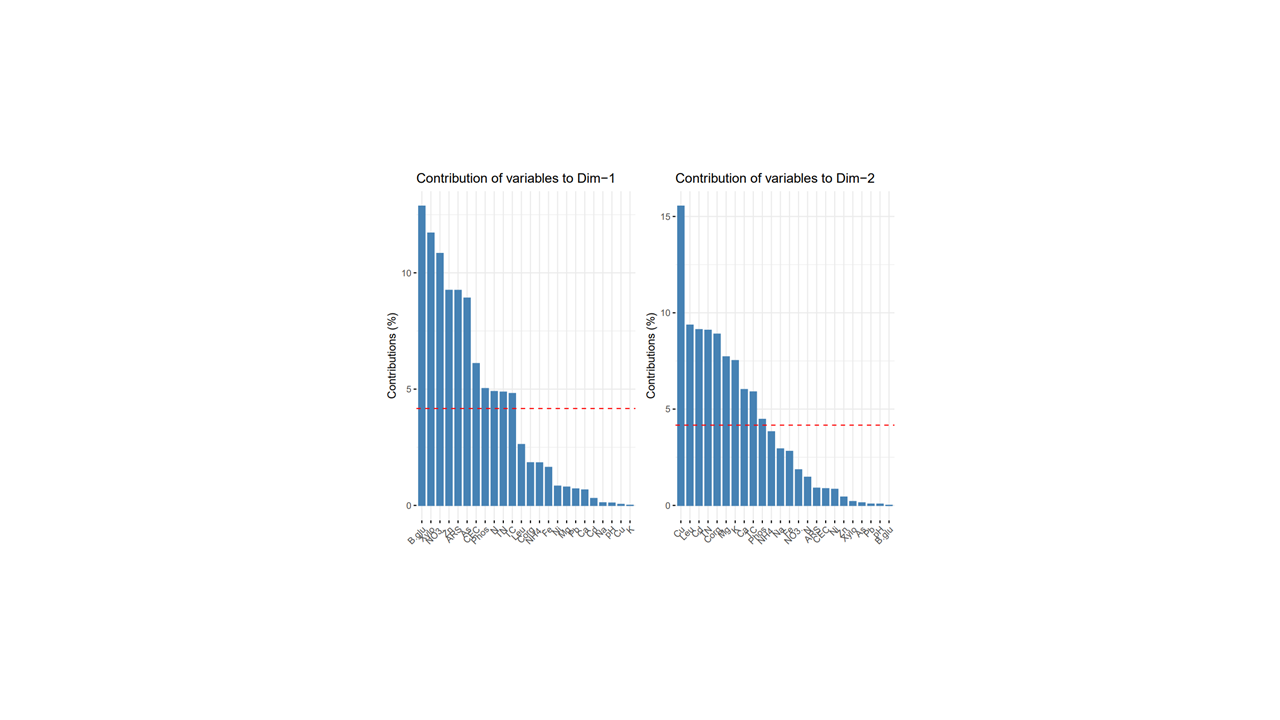

Supplement: S3 Fig — The red line represents the average contribution threshold if all the variables had equal contributions. TC = HWC, TN = HWN. (TIF) [file pone.0338385.s009.tif]

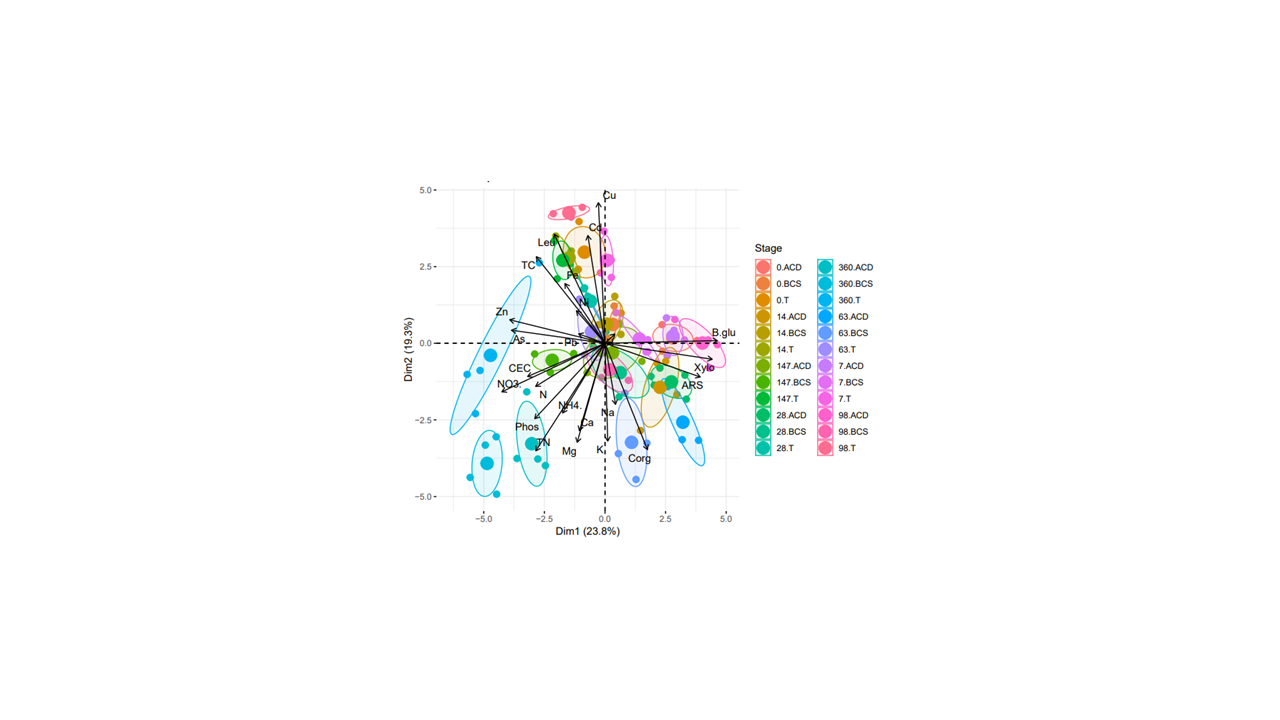

Supplement: S4 Fig — Points represent the coordinates of each sample according to the 2 dimensions of PCA2. TC = HWC, TN = HWN. Confidence ellipses (95%) were plotted for each treatment. (TIF) [file pone.0338385.s010.tif]

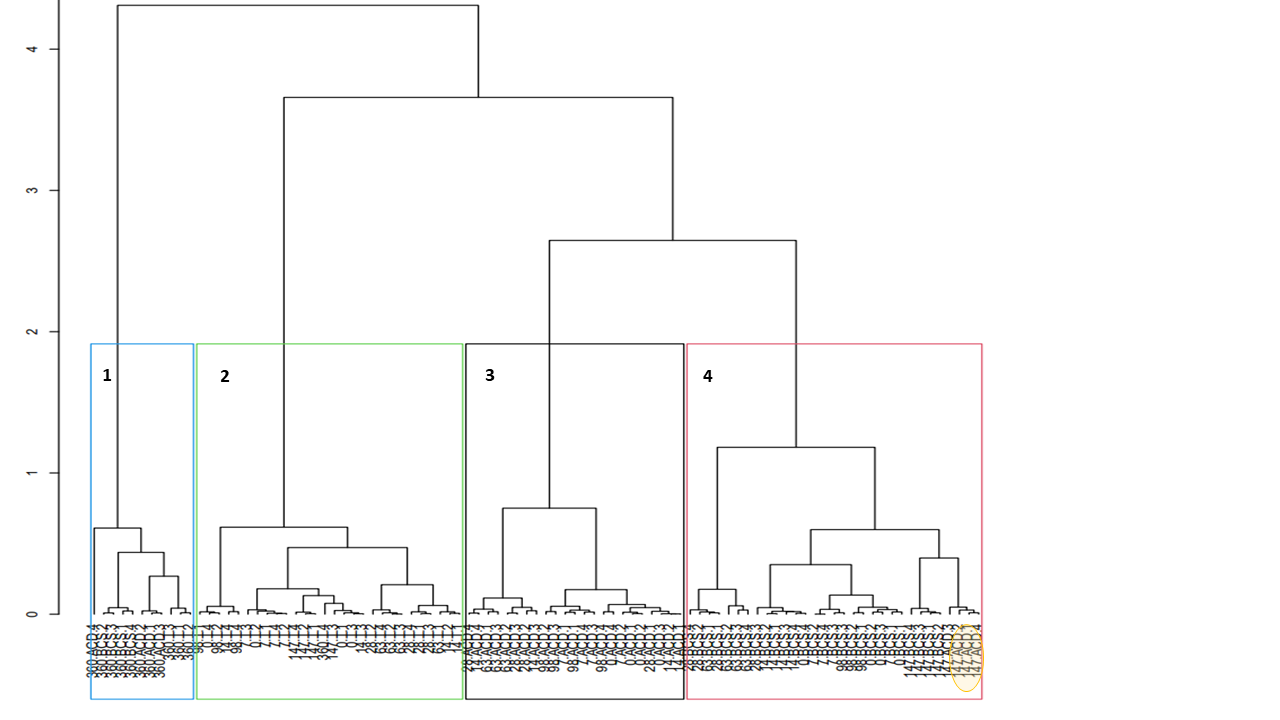

Supplement: S5 Fig — BCS: Sargassum spp. Biochar, ACD: Activated carbon DARCO®, UA: Unamended soil. Cluster 1: Day 360 (UA, BCS and ACD), Cluster 2: UA (Days 0, 7, 14, 28, 63, 98 and 147), Cluster 3: ACD (Days 0, 7, 14, 28, 63 and 98), Cluster 4: BCS (Days 0, 7, 14, 28, 63, 98 and147) + ACD (Days 147). Yellow indicates the enclave of ACD individual samples on Day147. (TIF) [file pone.0338385.s011.tif]

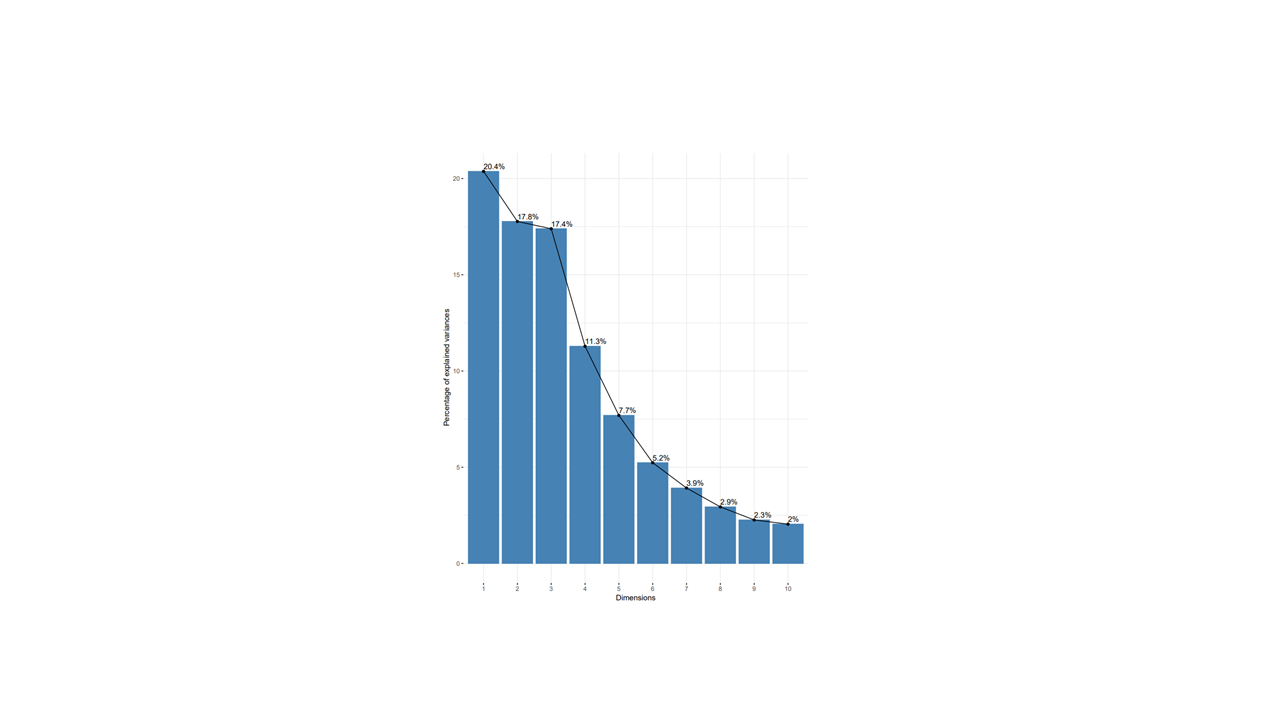

Supplement: S6 Fig — (TIF) [file pone.0338385.s012.tif]

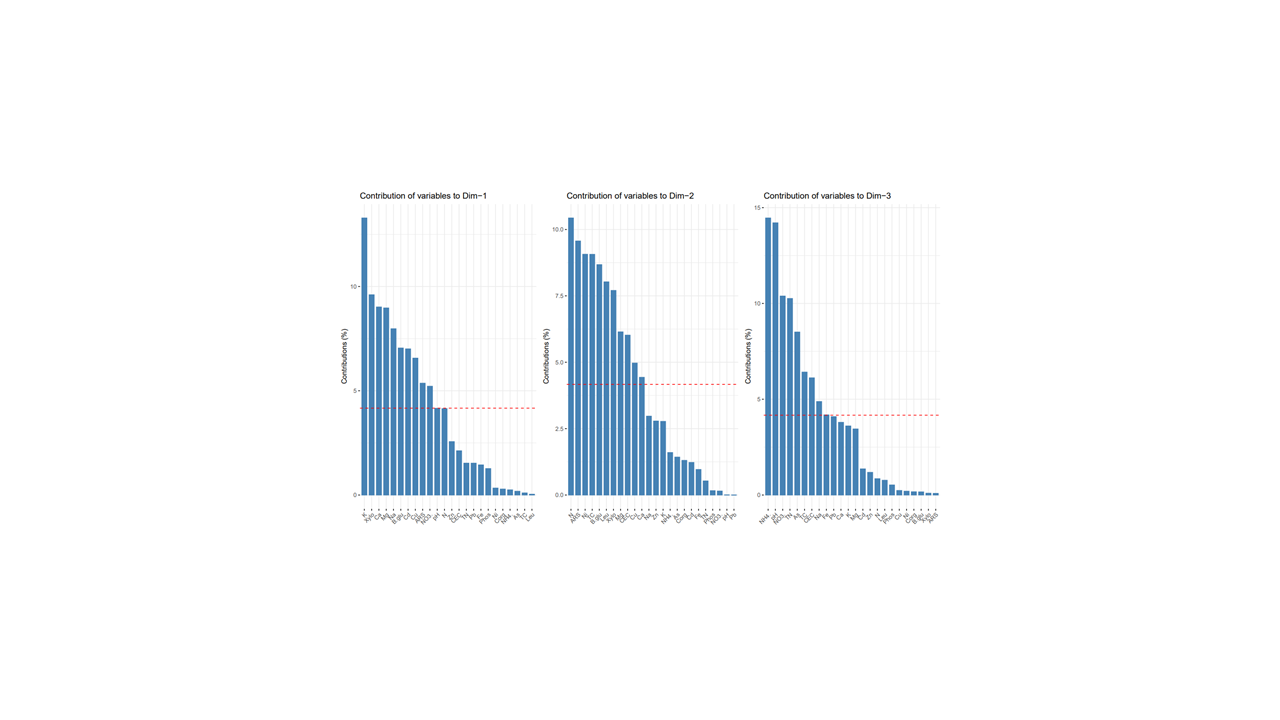

Supplement: S7 Fig — The red line represents the average contribution threshold if all the variables had equal contributions. TC = HWC, TN = HWN. (TIF) [file pone.0338385.s013.tif]

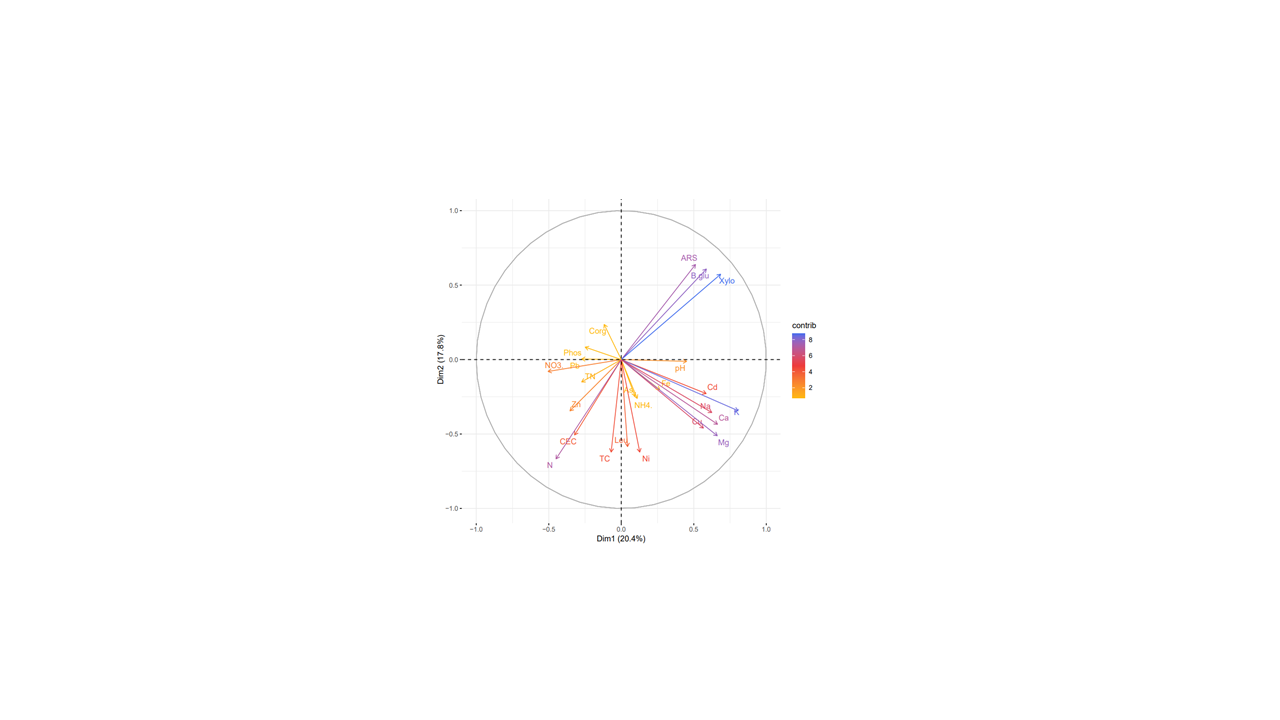

Supplement: S8 Fig — TC = HWC, TN = HWN. (TIF) [file pone.0338385.s014.tif]

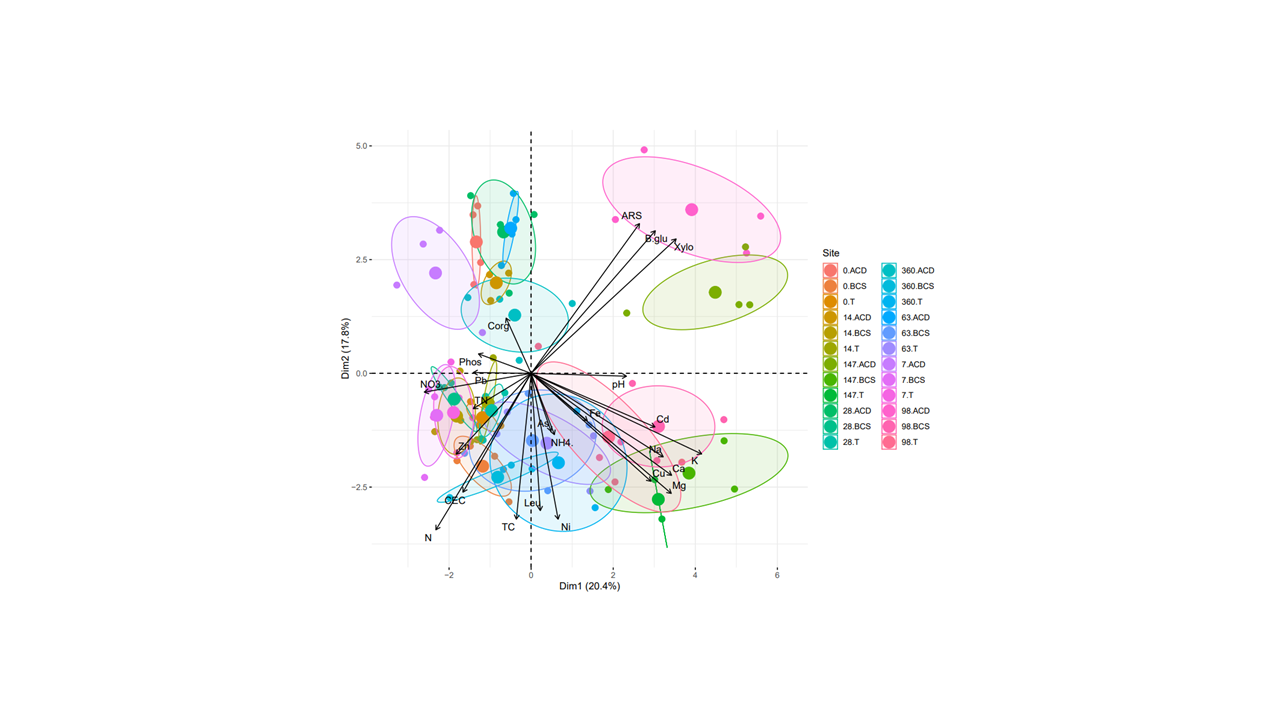

Supplement: S9 Fig — Points represent the coordinates of each individual sample according to the 2 dimensions of PCA3. TC = HWC, TN = HWN. Confidence ellipses (95%) were plotted for each treatment. (TIF) [file pone.0338385.s015.tif]

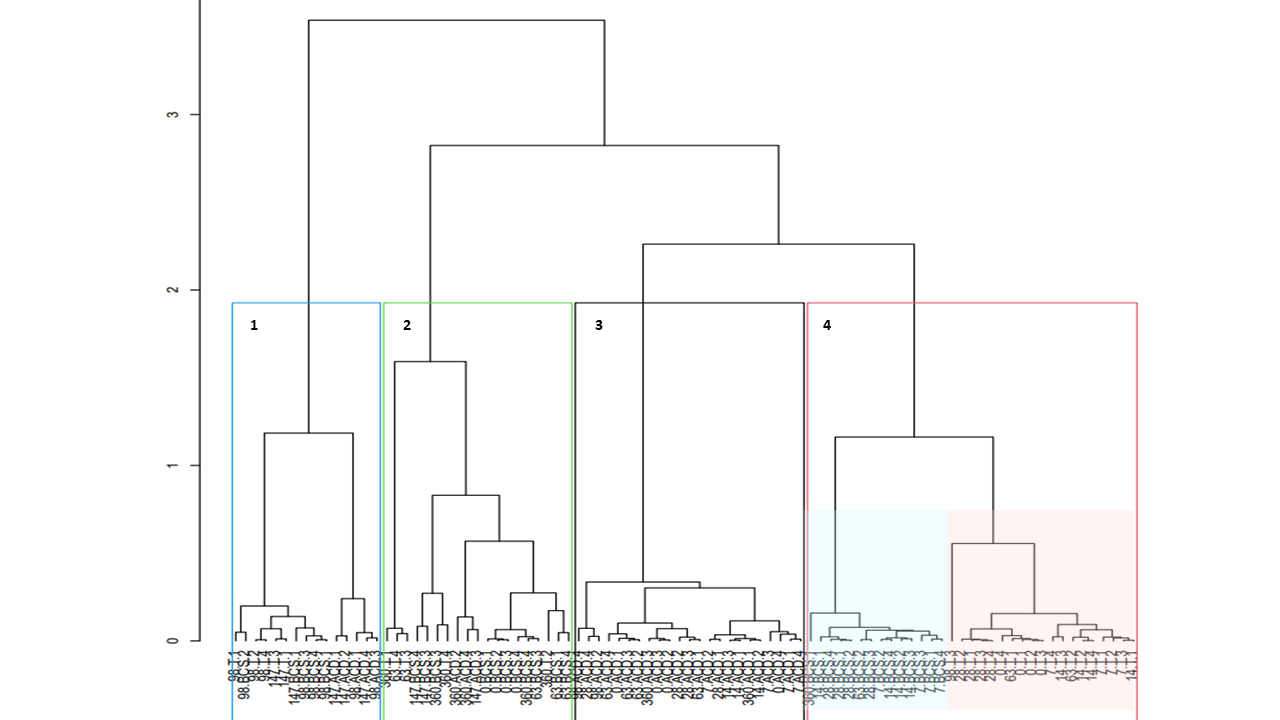

Supplement: S10 Fig — BCS: Sargassum spp. Biochar, ACD: Activated carbon DARCO®, T: Unamended soil (UA). Cluster 1: BCS (Day 98) + UA (Days 98 and 147) + ACD (Days 98 and 147), Cluster 2: BCS (Days 0, 63, 147 and 360) + UA (Days 63 and 360) + ACD (Days 360), Cluster 3: ACD (Days 0, 7, 14, 28, 63, 98 and 360), Cluster 4: BCS (Days 7, 14 and 28) + UA (Days 0,7 14 and 28). (TIF) [file pone.0338385.s016.tif]

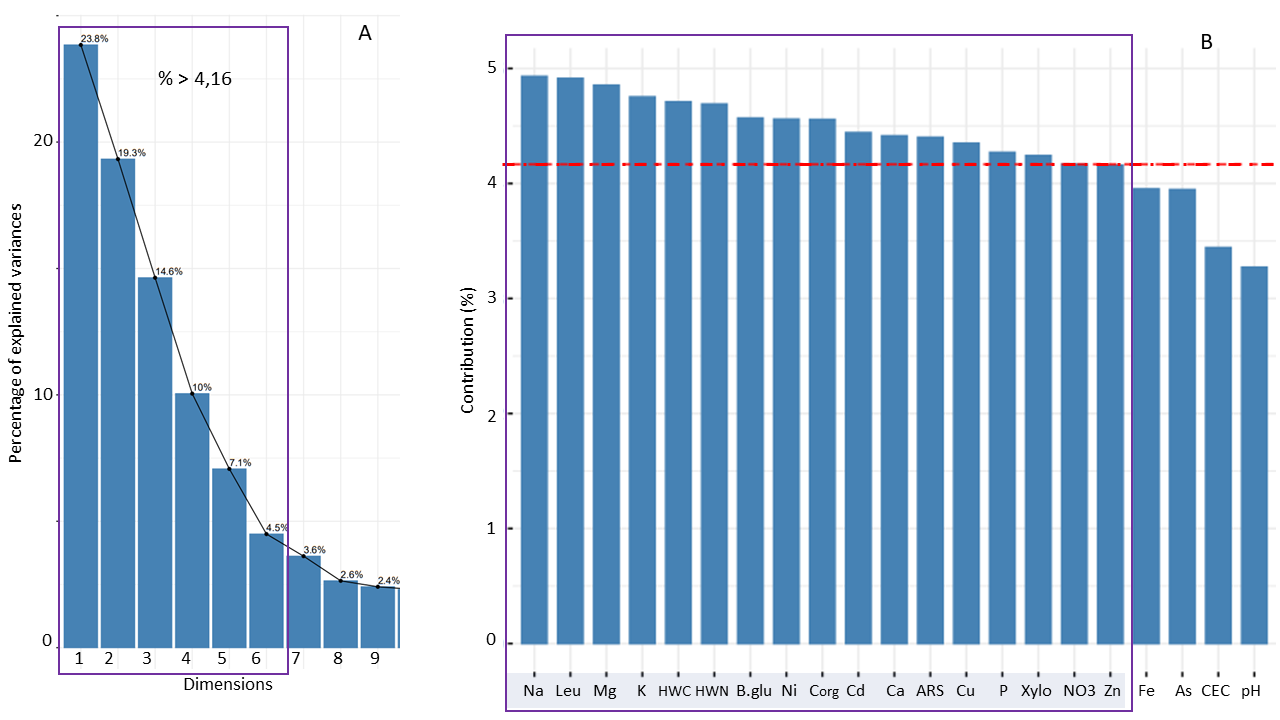

Supplement: S11 Fig — (TIF) [file pone.0338385.s017.tif]

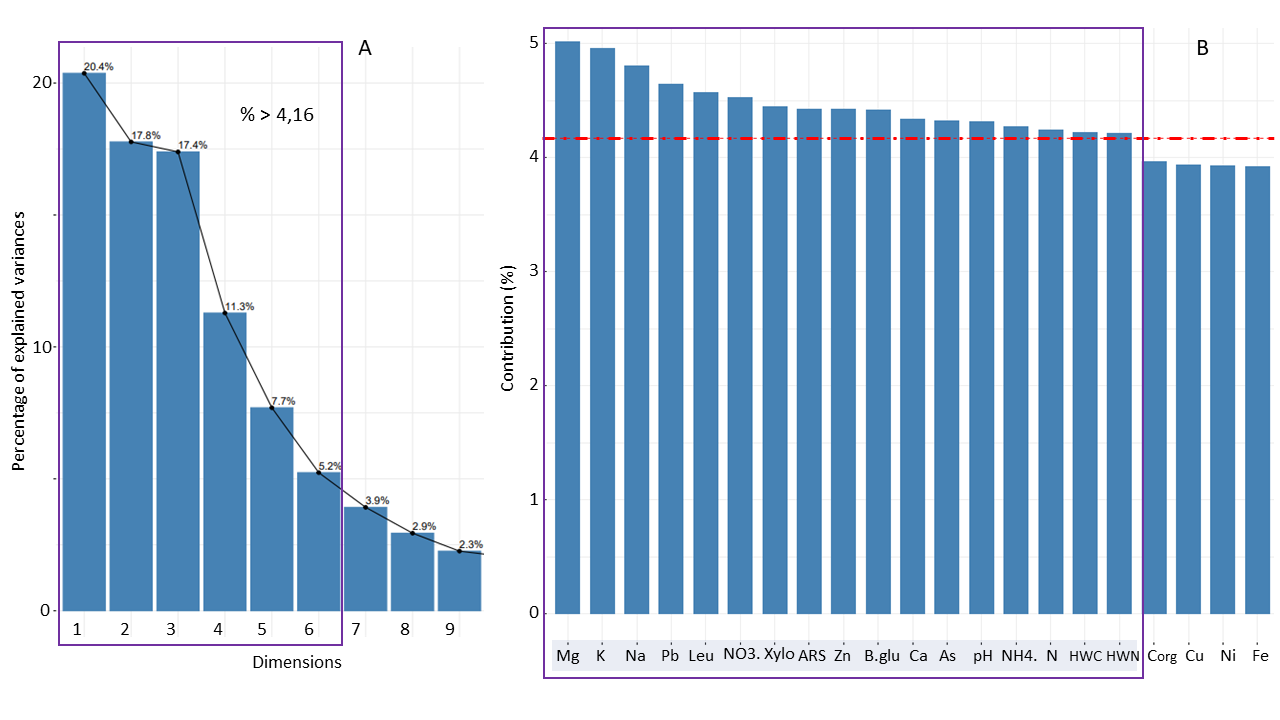

Supplement: S12 Fig — TN: Labile nitrogen (HWN), TC: Labile carbon (HWC). (TIF) [file pone.0338385.s018.tif]

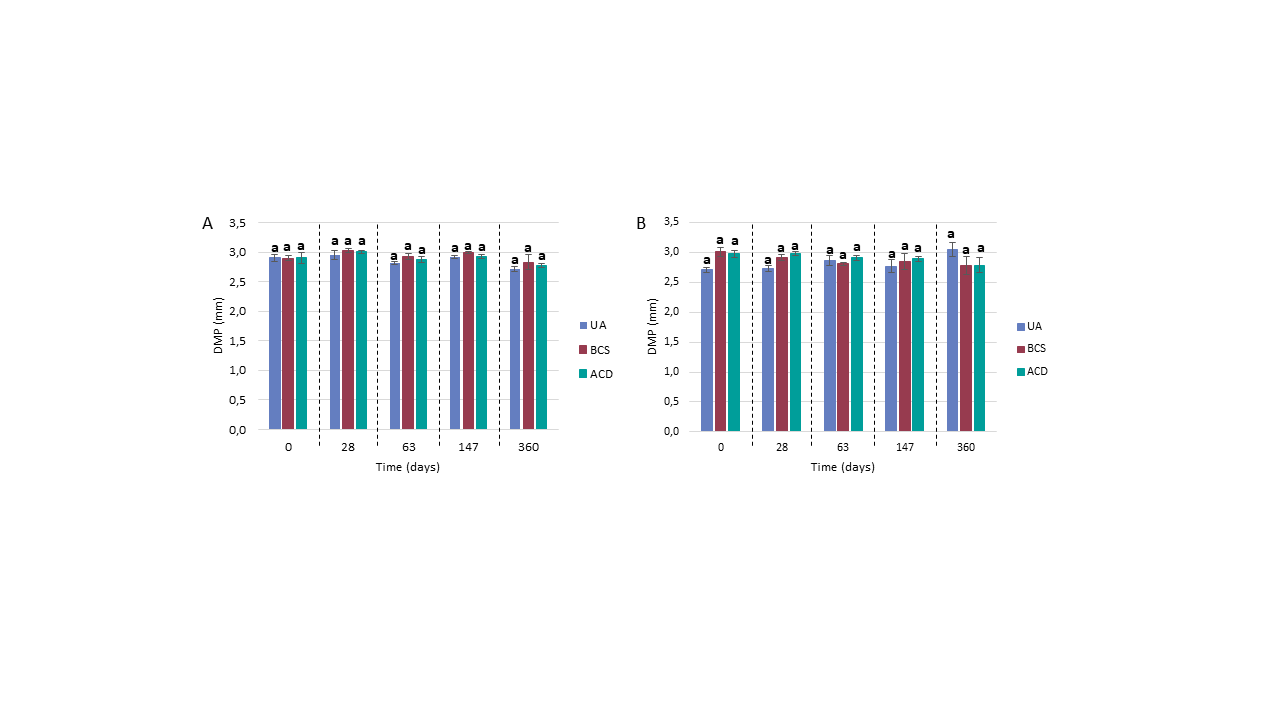

Supplement: S13 Fig — UA: unamended soil. BCS: Biochar of Sargassum spp. ACD: Activated carbon DARCO®. The values correspond to the mean ± SE (n = 4). Mean values with different superscripted letters for the same stage are statistically different (p-value< 0.05) between modalities (ANOVA test). (TIF) [file pone.0338385.s019.tif]

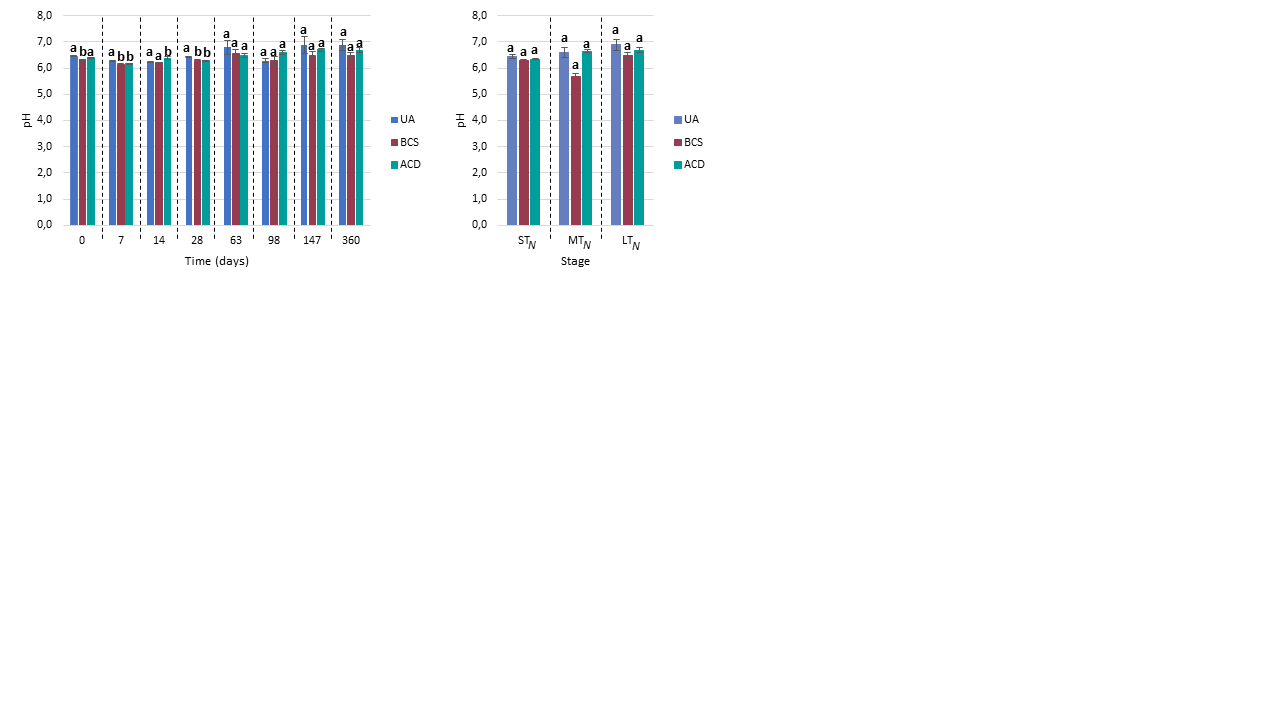

Supplement: S14 Fig — UA: unamended soil. BCS: Biochar of Sargassum spp. ACD: Activated carbon DARCO®. STN: Short-term Nitisol (Days 0, 7, 14, 28 and 63), MTN: Medium-term Nitisol (Days 98 and 147), LTN: Long term Nitisol (Day 360). The values correspond to the mean ± SE (n = 4). Mean values with different superscripted letters for the same day (a, b, c) are statistically different (P < 0.05) between modalities (according to the ANOVA test). (TIF) [file pone.0338385.s020.tif]

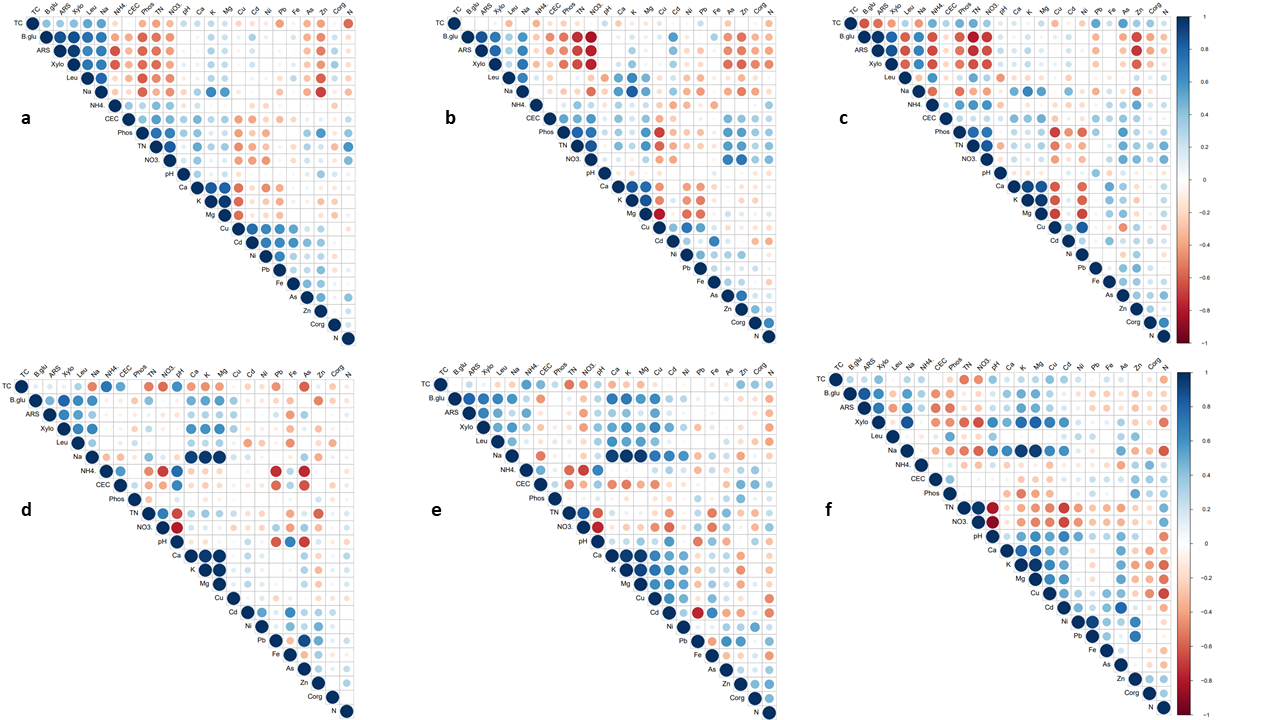

Supplement: S15 Fig — (a) Andosol without amendment, (b) Andosol + BCS, (c) Andosol + ACD, (d) Nitisol without amendment, (e) Nitisol + BCS and (f) Nitisol + ACD. TN = HWN, TC = HW. (TIF) [file pone.0338385.s021.tif]
